# Emerging Aluminum‐Ion Capacitors and Lessons from Battery Chemistry

**DOI:** 10.1002/smll.75042

**Published:** 2026-08-13

**Authors:** Smita Talande, Ievgen Obraztsov, Vishal Shrivastav, Mahima Khandelwal, Mansi, Michal Otyepka, Radek Zbořil, Aristides Bakandritsos

**Affiliations:** ^1^ Regional Centre of Advanced Technologies and Materials Czech Advanced Technology and Research Institute (CATRIN) Palacky University Olomouc Olomouc Czech Republic; ^2^ IT4Innovations VSB–Technical University of Ostrava Ostrava‐Poruba Czech Republic; ^3^ Nanotechnology Centre Centre For Energy and Environmental Technologies VSB–Technical University of Ostrava Ostrava‐Poruba Czech Republic

**Keywords:** capacitor, charge carrier, electrolyte, energy storage, graphene, mxenes, nanotechnology, solvation, supercapacitor

## Abstract

Aluminum‐ion capacitors (AICs) attract significant research interest as multivalent energy‐storage devices that aim to increase energy density beyond that of conventional monovalent‐ion supercapacitors. Owing to the trivalent charge of Al^3+^ and the abundance, low cost, and relative chemical stability of aluminum, AICs hold strong potential for sustainable and scalable energy storage. This review provides a comprehensive overview of their fundamental charge‐storage mechanisms, recent advances in electrode materials, electrolyte engineering, and device architectures. Particular emphasis is placed on overcoming the intrinsic challenges associated with Al^3+^‐based charge carriers, such as intricate solvation chemistry, sluggish desolvation kinetics, and limited ion transport within host frameworks, through structural design, defect engineering, and interfacial optimization. Progress in aqueous, ionic‐liquid, deep‐eutectic, and quasi‐solid‐state electrolytes is critically assessed alongside the key role of 2D materials, such as graphene and MXenes. Finally, insights from operando characterization and computational modeling are discussed to guide future directions toward high‐rate, long‐life, and flexible Al^3+^‐based supercapacitors. By integrating these perspectives, this review provides a conceptual framework and roadmap for advancing AICs toward next‐generation energy storage.

## Graphical abstract

Al‐ion energy storage is gaining momentum as demand grows for safer, lower‐cost, and more sustainable alternatives to Li‐based technologies. Aluminum's abundance, recyclability, and trivalent charge offer high charge capacity and scalable device potential. The central challenge is translating this chemistry into fast, reversible storage despite strong Al^3+^ solvation, complex electrolytes, corrosion, and sluggish ion transport.

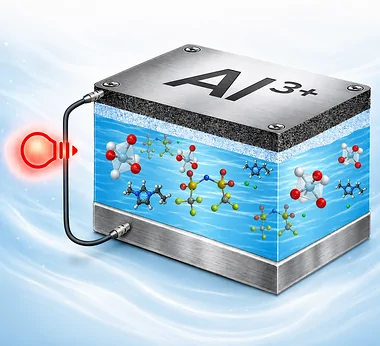

## 1 Introduction

### 1.1 Background on Supercapacitors and Their Classification

Over the decades, extensive reliance on fossil fuels has contributed to multiple global challenges, including deteriorating air quality, accelerating climate change, and intensifying geopolitical concerns over energy security, and over the availability of critical materials (e.g., lithium and cobalt). However, the growing demand for electrical energy to power vehicles, smartphones, computers, and numerous other electronic devices highlights the urgent need for environmentally friendly and sustainable electrochemical energy storage (EES) technologies. Compared with many battery chemistries, supercapacitors generally offer lower fire risk and simpler recycling, although overall safety depends on electrolyte and design. Unlike lithium‐based EES systems that rely on increasingly scarce Li resources, as well as critical transition metals such as Ni and Co, aluminum‐based systems can in principle use more earth‐abundant materials, which could help reduce costs and supply‐chain risks [1, 2, 3]. Supercapacitors have therefore attracted extensive research interest because they combine high power density (fast charge–discharge capability), longer cycle life than batteries, and higher energy density relative to conventional dielectric capacitors [4, 5, 6]. Recent advances in flexible and wearable device architectures, environmentally sustainable electrode materials, and integration into hybrid energy storage systems further highlight their potential for next‐generation electronics and electric vehicles [7, 8, 9, 10, 11].

Electric double‐layer capacitors (EDLCs) are recognized for their ability to deliver high power densities, enable rapid charge–discharge processes, and sustain outstanding cycle lifetimes, often surpassing one million cycles [12, 13]. Their electrostatic storage mechanism, which relies on ion adsorption at the electrode–electrolyte interface, minimizes chemical degradation and contributes to excellent reversibility, safety, and energy efficiency [14, 15]. These properties make EDLCs particularly attractive for applications requiring quick energy delivery and long operational lifetimes. Despite these advantages, EDLCs continue to face critical challenges, particularly low energy density and restricted cell voltage. These drawbacks limit their suitability for applications requiring substantial energy storage. However, ongoing research into advanced porous carbons, novel electrolytes, and hybrid capacitor (HC) systems offers promising avenues to overcome these limitations [16]. For example, multiscale graphene architectures [17], created through rapid thermal annealing, introduce curved turbostratic crystallites interwoven with disordered domains, which facilitate efficient ion transport while maintaining high packing density. Such tailored structures enable precise pore ion matching and partial charge transfer, with reported exceptional volumetric energy densities in both organic (∼49 Wh L^−1^) and ionic liquid electrolytes (∼99 Wh L^−1^), along with high power densities at the stack level. In another example, nitrogen‐doped graphene with diamond‐like interlayer bonds achieved unprecedented volumetric energy densities of 200 Wh L^−1^ at 2.6 kW L^−1^ and ∼143 Wh L^−1^ at 52 kW L^−1^ in an 1‐ethyl‐3‐methylimidazolium tetrafluoroborate/1,1,2,2‐tetrafluoroethyl‐2,2,3,3‐tetrafluoropropyl ether ionic‐liquid electrolyte [18]. These breakthroughs demonstrate how nanoscale and mesoscale engineering of carbon frameworks can overcome traditional trade‐offs between energy density, power density, and compact device design. Beyond pure EDLCs, which store charge via electrostatic interactions, pseudocapacitors also store charge through fast and reversible faradaic reactions occurring at or near the electrode surface, often involving transition‐metal oxides, conductive polymers, or heteroatom‐doped carbons [12, 19, 20]. In such systems, ions often intercalate or adsorb over a continuous potential range, rather than at a sharp battery‐like redox plateau, without triggering bulk phase transformations [20, 21]. Metal‐ion hybrid capacitors are another key emerging category. They combine a battery‑type (faradaic) electrode, usually the anode, with a capacitor‑type electrode, usually the cathode, to bridge energy and power [22, 23, 24].

**FIGURE 1:**
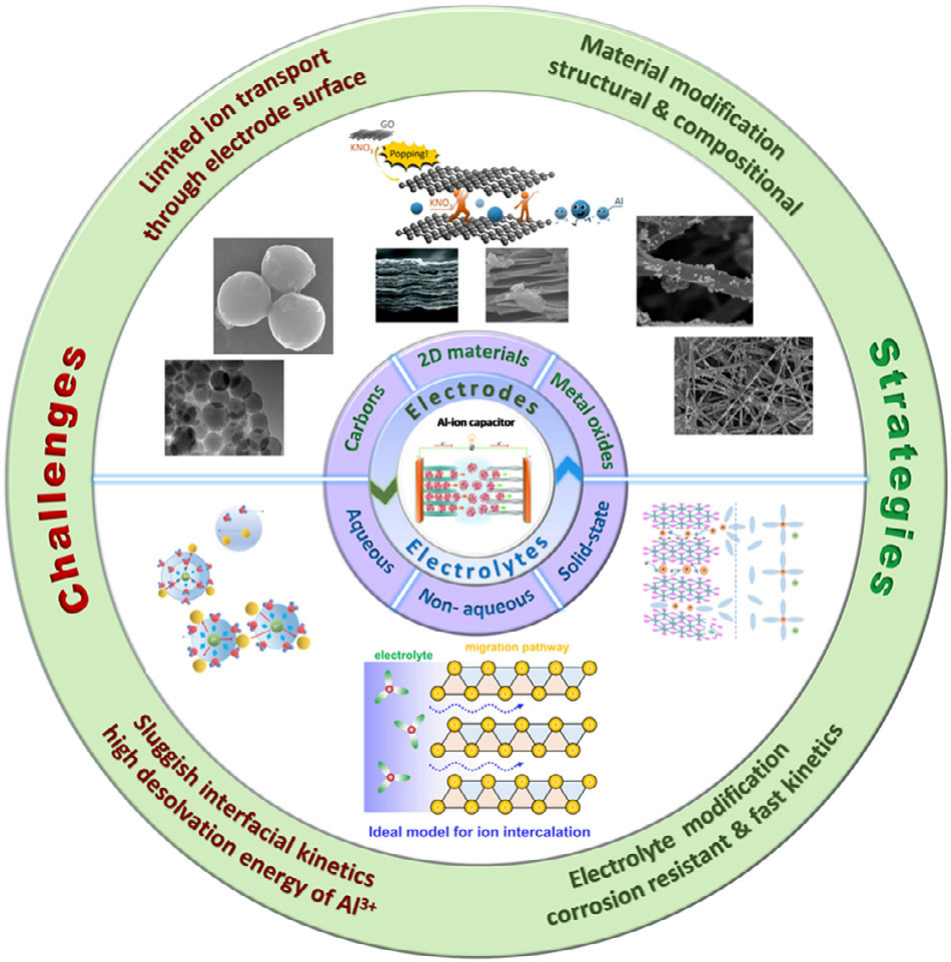
Overview of mechanisms, functions, features, materials and electrolytes studied and developed for AICs [27, 28, 29, 30, 31, 32, 33, 34].

Reported aluminum‐ion capacitor (AIC) systems span several device categories, where charge storage relies on adsorption (i.e., electrical double‐layer (EDL) formation), pseudocapacitive contributions, fast intercalation, and/or metal deposition/stripping (i.e., hybrid charge‐storage mechanisms). Hybrid charge storage can improve capacitance and energy density and, in favorable cases, preserve fast charge–discharge kinetics, thereby narrowing the performance gap between EDLCs and batteries [13, 25]. Nevertheless, they often face challenges such as limited electrode stability, degradation over cycling, and, in metal‐ion HC systems involving metal plating/stripping, dendrite formation, as well as higher production costs than carbon‐based EDLCs [20, 26]. To address these issues, ongoing research on nanostructuring, integration with conductive carbon matrices, and electrolyte engineering has shown considerable promise in improving both the rate capability and long‐term durability of AICs (Figure 1) [22].

Intercalation pseudocapacitance arises when ions from the electrolyte reversibly insert into the crystal lattice of an electrode material without causing significant structural changes, thereby enabling rapid and reversible charge storage [12, 19, 22]. Unlike conventional battery intercalation, which often involves diffusion‐limited processes and phase transformations, an intercalation‐type charge‐storage mechanism occurs through reversible ion accommodation within available lattice sites, thereby supporting rapid kinetics [25]. This mechanism allows materials such as transition‐metal oxides (e.g., Nb_2_O_5_ [35, 36], MoO_3_ [37], Fe_2_O_3_ [38]) and MXenes [39] to combine high charge storage with rapid ion kinetics, bridging the gap between supercapacitors and batteries. Devices employing such electrodes may, depending on the full‐cell configuration, be classified as HCs, which can deliver higher energy densities than EDLCs while retaining comparatively high power output and long cycle life. Furthermore, metal‐ion HCs can be categorized according to the charge carrier; monovalent‐ion hybrid capacitors (Li^+^, Na^+^, and K^+^) [40, 41, 42, 43, 44] and multivalent‐ion hybrid capacitors (Mg^2+^, Zn^2+^, Ca^2+^, Al^3+^) [27, 45, 46, 47]. Multivalent chemistries are attractive because each charge carrier can, in principle, transfer more than one electron, which may increase theoretical volumetric capacity and energy density. However, the higher charge density of multivalent ions also strengthens ion–solvent and ion–host interactions, which commonly results in larger desolvation barriers and slower solid‐state diffusion than in monovalent systems.

**SCHEME 1:**
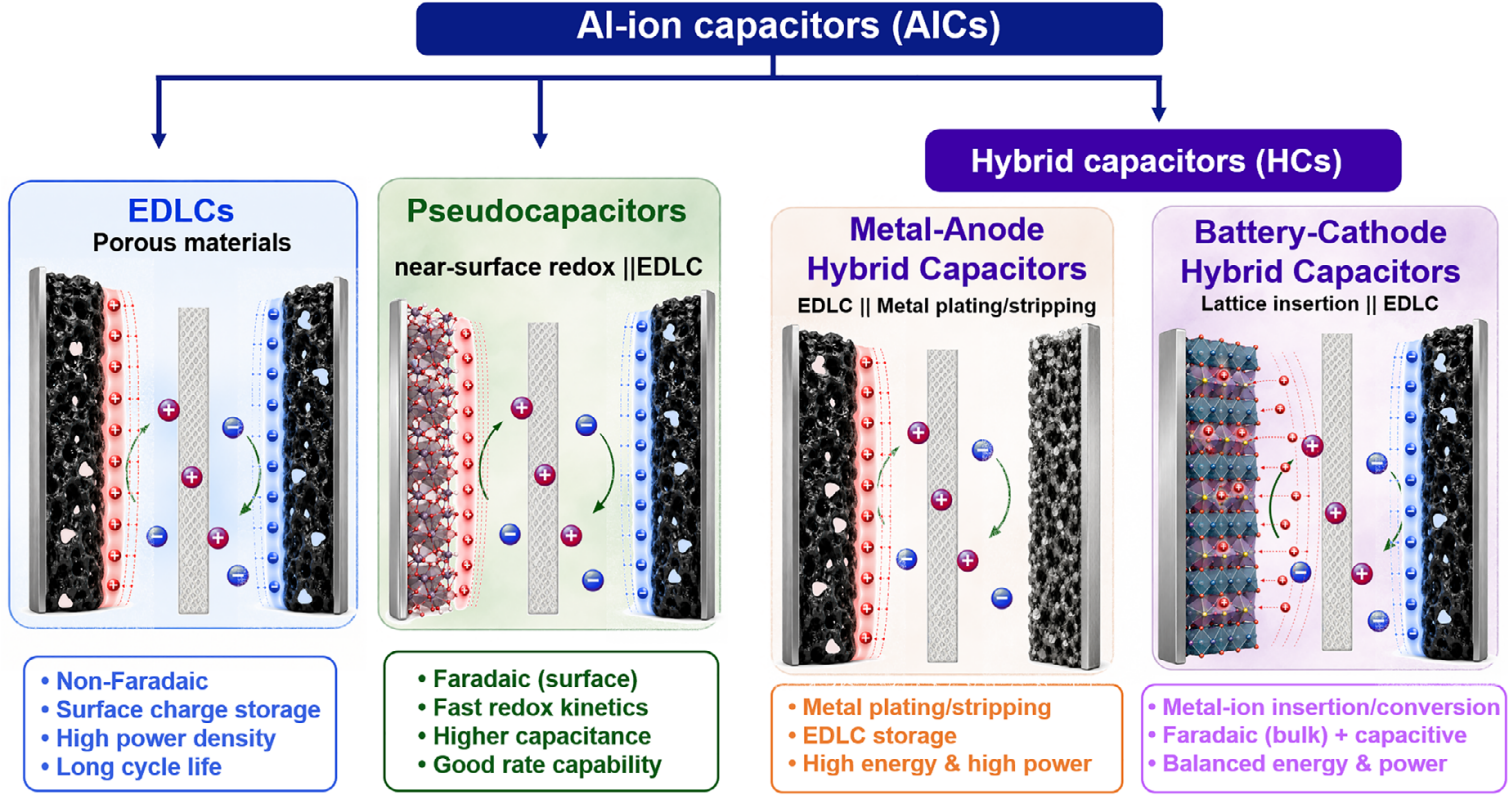
Classification of AICs (and of supercapacitors in general) based on charge storage mechanism.

In this review, AICs are classified according to the underlying electrochemical mechanism of the device, using galvanostatic charge–discharge (GCD) characteristics as an initial descriptor together with complementary electrochemical and structural evidence (Scheme 1). Materials exhibiting nearly ideal triangular and highly symmetric GCD profiles are categorized as electric double‐layer capacitors (EDLCs), indicating predominantly non‐faradaic, surface‐controlled charge storage. Systems showing deviations from linearity, without pronounced voltage plateaus, are classified as pseudocapacitive, where fast and reversible surface or near‐surface faradaic reactions contribute to capacitance while maintaining capacitive‐like kinetics [13]. Finally, systems which utilize battery‐type materials on one electrode and a capacitive‐type material on the other electrode are also termed HCs. They may display varying degrees of non‐linearity in the charge–discharge profiles. In particular, such HCs are full cells with i) a battery‐type cathode material (i.e., where metal cations enter into the crystal lattice), and a supercapacitor‐type counter electrode (i.e. a porous material) or ii) a battery‐type anode process involving reduction/plating and oxidation/stripping of the metal (with or without the presence of a host such as graphite in lithium‐ion batteries) and a capacitive‐type material on the counter electrode [25]. This classification framework enables clearer differentiation between capacitive and faradaic‐dominated mechanisms beyond simple performance metrics. For metal‐ion hybrid capacitors (MIHCs), the term X‐HCs is used, where X is the corresponding metal, for example Al, Li, Na, or Mg.

### 1.2 Limitations of Monovalent‐Ion Capacitors

Monovalent‐ion capacitors have attracted extensive interest because Li^+^, Na^+^, and K^+^ offer relatively favorable solvated‐ion transport, well‐established intercalation/redox chemistry, and compatibility with carbonaceous and pseudocapacitive electrodes [23]. Yet, these systems are often limited by fundamental trade‐offs between energy density and fast kinetics. Although Li‐ion hybrid capacitors can achieve remarkably high gravimetric and volumetric energy densities, their broader deployment is hindered by resource scarcity, elevated material costs, and electrode–electrolyte interfacial instabilities at high current densities [44]. Economically recoverable lithium reserves are unevenly distributed across the globe and concentrated in a limited number of countries, which raises concerns about long‐term supply security and price volatility. Sodium and potassium metal‐ion capacitors, although more abundant, present processing barriers due to high reactivity [48, 49]. In addition, safety challenges such as dendrite growth and electrolyte reactivity further compromise operational stability [50].

Among the monovalent ions, Na^+^ and K^+^ exhibit slower diffusion kinetics arising from their larger ionic radii, which increases ion transport resistance and limits high‐rate performance [44, 51, 52]. Repeated cycling further induces structural stress and mechanical fatigue in electrode frameworks, ultimately compromising long‐term stability. Consequently, monovalent‐ion capacitors often struggle to achieve a balance between high energy density and durable cycling performance. These challenges have propelled increasing attention toward multivalent‐ion systems (Mg^2+^, Zn^2+^, Ca^2+^, Al^3+^), where the ability to transfer multiple charges per ion offers a pathway to significantly boost storage capacity and bridge the performance gap between conventional supercapacitors and batteries [24, 53]. Moreover, such strategies align with the ongoing search for alternative charge carriers that integrate high per‐ion capacity with improved sustainability.

### 1.3 Perspective on the Development of AICs

Multivalent ions, such as Al^3+^, Mg^2+^, Zn^2+^, and Ca^2+^, can transfer multiple charges per ion, offering the possibility of higher capacity and energy density if reversibility, voltage, and kinetics are maintained [24, 54]. Among these, aluminum ions (Al^3+^) have received particular attention due to their unique combination of high theoretical capacity (as a metal anode), natural abundance, cost‐effectiveness, and environmental compatibility [23, 55]. Furthermore, Al‐based energy‐storage chemistries are attractive for sustainability because aluminum combines particularly high natural abundance (Al: 8.23% > Ca: 4.15% > Na: 2.36% > Mg: 2.33% > K: 2.09% > Zn: 0.007% > Li: 0.002%), high ionic charge density (Table 1), passivating surface chemistry that enables cost‐effective handling and manufacturing, and a favorable safety profile [48], which simplifies processing and storage under ambient conditions. These features underscore the potential of Al‐based energy‐storage systems for practical applications (Table 1).

**TABLE 1:** Comparison of monovalent vs. multivalent‐ion EES parameters.

Ions	Li^+^	Na^+^	K^+^	Zn^2+^	Mg^2+^	Ca^2+^	Al^3+^
Ionic radius^a^	0.76 Å	1.02 Å	1.33 Å	0.74 Å	0.72 Å	1.00 Å	0.53 Å
Ionic charge density (C mm^−3^)	52	24	11	112	120	52	364
Approximate Cost (USD kg^−1^)	$11	$0.65	$1.04	$3.1	$3.5	$2.5	$2.5^b^
Abundance (Percent of Earth's crust)	0.002%	2.3%	2.1%	0.0079%	2.3%	4.15%	8.23%

^a^ Effective ionic radii correspond to sixfold coordination and were taken from Shannon [229]. Ionic radii depend on oxidation state and coordination number.

^b^ Use of recycled Al could cost only ∼ $0.9.

Despite these advantages, practical AICs are limited by electrolyte‐dependent Al speciation, sluggish interfacial charge transfer, restricted access of Al‐bearing species to confined pores or host lattices, and interfacial instability [56, 57]. These coupled kinetic and stability limitations are discussed in detail in Section 2.4. In addition, achieving stable and conductive electrode–electrolyte interfaces is critical, as interfacial instability can lead to capacity fading and diminished cycling stability [58]. Finally, the complex electrochemical behavior of Al^3+^, which can involve pseudocapacitive surface redox/adsorption, fast intercalation‐type processes, and, depending on the electrolyte, complex‐ion insertion and/or side reactions (e.g., hydrolysis and passivation) requires deeper mechanistic understanding to optimize charge storage pathways.

**FIGURE 2:**
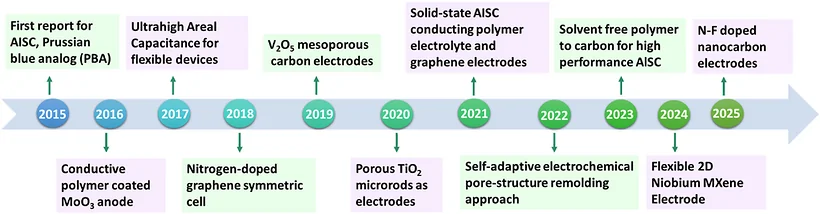
Brief development history of AICs [27, 33, 64, 65, 66, 67, 68, 69, 70, 71].

To overcome these limitations, research has focused on multiple strategies. Engineering electrode materials with expanded lattice structures, two‐dimensional (2D) layered structures, or hierarchical porosity can facilitate Al^3+^ diffusion and enhance charge accessibility (Figure 1) [27, 59]. Optimizing electrolytes to enable efficient and reversible Al^3+^ transport while maintaining interfacial stability is another key approach. Interface engineering, including surface modifications or protective coatings, has also been employed to prevent degradation and ensure long‐term reliability for aluminum‐ion batteries (AIBs) [60, 61, 62, 63], and represents an effective strategy for advancing the development of AICs. Collectively, these strategies aim to exploit the high theoretical capacity associated with aluminum chemistry, while mitigating the kinetic and stability challenges that currently hinder its broader application (Figure 2). Al^3+^‐based capacitors offer a compelling approach to bridging the performance gap between conventional capacitors and batteries. Li et al. provided one of the earliest demonstrations of an Al‐ion hybrid capacitor (Al‐HC), coupling a Prussian blue analogue cathode with reversible Al^3+^ intercalation in an asymmetric configuration in 2015 [64]. This work established the foundation for subsequent research on Al‐ion capacitive energy storage. While notable progress has been achieved, continued efforts in electrode design, electrolyte development, and interface optimization are essential to fully explore their potential for high‐performance capacitive energy storage.

### 1.4 Scope and Structure of the Review

In this review, we present a comprehensive analysis of AICs, focusing on the fundamental charge storage mechanisms, electrolyte evolution, and material design strategies that define their performance. Particular emphasis is placed on the interplay between Al^3+^ speciation, electrode architecture, and interfacial chemistry, which collectively govern energy density, rate capability, and long‐term stability. Both aqueous and non‐aqueous systems are critically examined, highlighting advances in water‐in‐salt electrolytes (WISE)/hydrated eutectic electrolyte (HEE) formulations, ionic liquid and deep eutectic solvent (DES) electrolytes, as well as emerging quasi‐solid‐state designs. Through a critical analysis of recent experimental, computational, and mechanistic studies, this review aims to establish a unified understanding of electrolyte–electrode interactions governing aluminum‐ion charge storage. Although the primary focus is on aluminum‐ion supercapacitors (AICs), selected studies on aluminum‐ion batteries (AIBs) are also discussed where they provide fundamental insights into Al‐species solvation, desolvation, ion transport, interfacial charge transfer, and electrode–electrolyte interactions that are directly relevant to AIC operation. By integrating knowledge across both fields, this review seeks to identify common mechanistic principles and outline future directions for the development of high‐energy, durable, and scalable AIC technologies.

## 2 Electrochemical Fundamentals of Al‐Ion Charge Storage

### 2.1 Intrinsic Properties of Al^3+^

**FIGURE 3:**
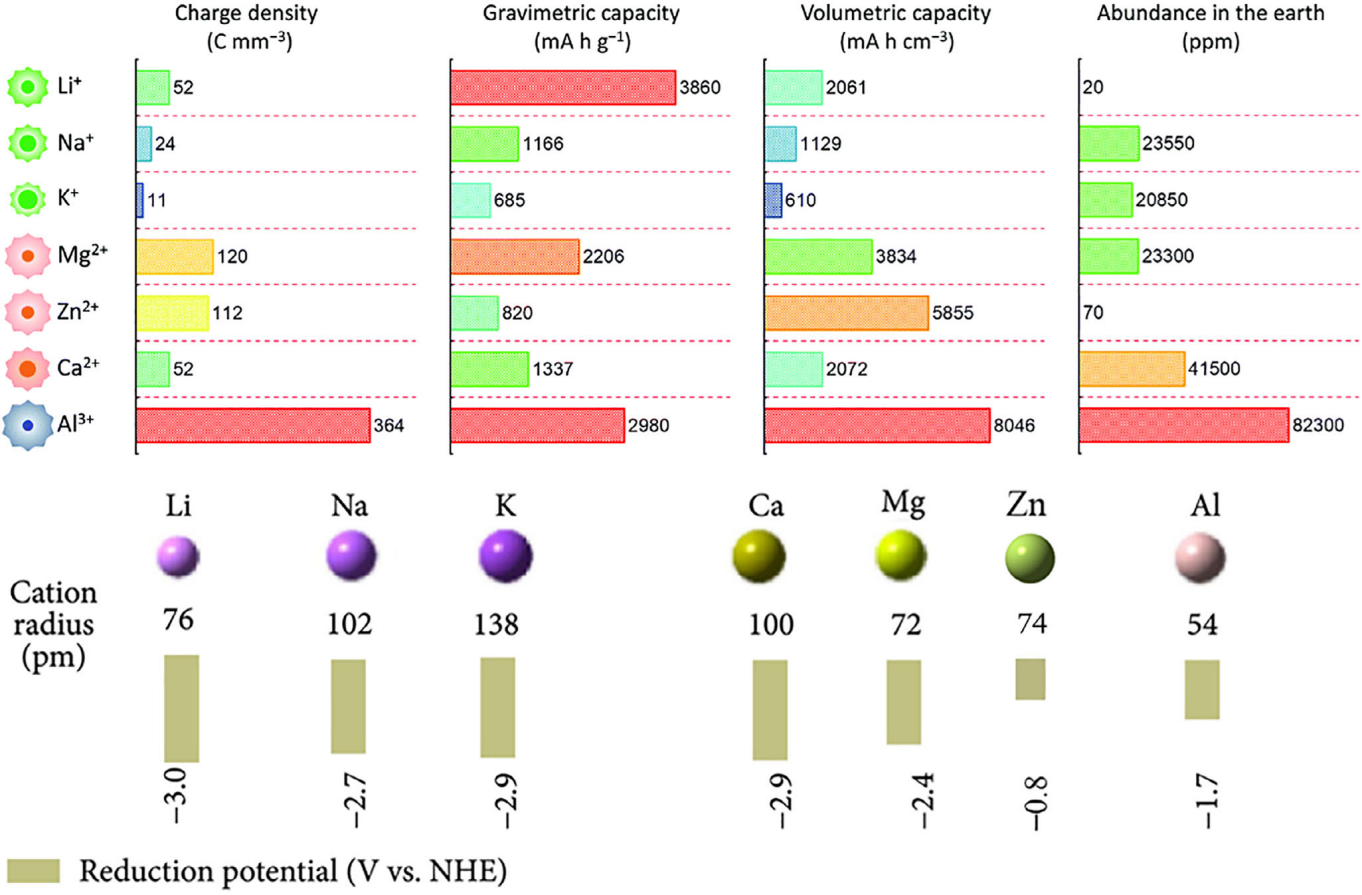
Comparison of the fundamental properties of Al^3+^ ions as charge carriers relative to other commonly used charge‐storage ions. Adapted from Ref. [27, 74] with permission from Royal Society of Chemistry, Copyright 2022 and AAAS, Copyright 2021.

Aluminum is one of Earth's most abundant metals, and its trivalent Al^3+^ ion is an attractive charge‐storage carrier because its high charge density enables a high theoretical charge per ion and can increase energy density (Figure 3) [27]. As a metal anode, aluminum offers high theoretical volumetric (∼8046 mAh cm^−3^) and gravimetric (2980 mAh g^−1^) capacities [72, 73]. Additionally, the Al–O distance in the primary hydration shell of aqueous [Al(H_2_O)_6_]^3+^ is about 1.87–1.90 Å, but this value describes the first‐shell metal‐oxygen bond distance rather than the hydrated ionic radius. The high charge density of Al^3+^ results in a high theoretical charge per carrier but also makes the identity and size of the active Al‐bearing species strongly dependent on the electrolyte. The consequences for desolvation, interfacial charge transfer, and host accessibility are further discussed in Section 2.4 [64]. These features can improve the performance of Al‐based energy storage systems, particularly in pseudocapacitors, where near‐surface diffusion is critical.

### 2.2 Electrolyte–Electrode Interactions for High‐Performance AICs

Electrolyte–electrode interactions strongly influence AIC performance by controlling ion transport, desolvation, and interfacial stability. Careful design of aqueous or non‐aqueous electrolytes is therefore essential to fully exploit Al^3+^ charge storage. Optimized electrolytes play a pivotal role in improving the reversibility and kinetics of ion insertion or adsorption, extending the usable voltage window, and, in selected systems, enhancing energy density, rate capability, and long‐term cycling stability. For instance, Al(TFSI)_3_ with 3‐cyanopropionic acid methyl ester (CPAME) showed improved voltage stability and charge storage in aluminum‐ion systems [75]. The bulky TFSI^−^ anions suppress side reactions and stabilize the electrode interface, while CPAME broadens the electrochemical window and mitigates solvent decomposition. These interactions promote more reversible Al^3+^ insertion/extraction and lead to uniform electrode surfaces with enhanced cycling stability. Such results highlight the importance of tailoring electrode–electrolyte interactions through coordinated salt–solvent design. Suitable ion–solvent interactions may boost conductivity and minimize electrolyte degradation, ensuring improved stability of the electrochemical cell [76]. Conventional aqueous aluminum‐salt electrolytes, such as concentrated Al(OTf)_3_ and aqueous AlCl_3_ or Al(NO_3_)_3_ solutions, often suffer from hydrolysis‐driven acidity, hydrogen evolution at sufficiently negative potentials, and interfacial instability; in chloride‐containing systems, corrosion can be particularly severe. These processes hinder reversible Al electrochemistry, promote passivation or parasitic reactions, and thereby limit the practical voltage window, Coulombic efficiency (CE), and cycling stability of Al‐based energy‐storage devices [48]. For example, highly reactive AlCl_3_ in aqueous solutions tends to hydrolyze, leading to the formation of acidic species that accelerate electrode degradation and promote unwanted side reactions such as hydrogen evolution [48, 77]. By mixing AlCl_3_ with LiTFSI to create a “water‐in‐salt” electrolyte, the interaction between AlCl_3_ and water is significantly reduced, preventing excessive hydrolysis (a common issue of AlCl_3_ electrolytes) and mitigating corrosiveness [78]. The hybrid electrolyte 1 M AlCl_3_/9 M LiTFSI modifies ion–solvent interactions and reduces AlCl_3_ hydrolysis, stabilizing the electrolyte environment, improving Al‐ion transport, and suppressing side reactions [79]. Furthermore, the combination of Al_2_(SO_4_)_3_ electrolyte and 2D h‐MoO_3_ electrodes significantly boosts Al‐ion storage performance by enhancing ionic conductivity and reducing charge transfer resistance [80]. Here the synergy between the electrolyte and electrode material, facilitated by oxygen vacancies and optimized ion transport, ensured efficient charge storage and fast kinetics. This highlights the critical role of selecting appropriate electrolyte–electrode pairs in designing high‐performance AICs.

In addition, enhanced electrolyte wetting improves charge–discharge kinetics and facilitates higher specific capacitance [81, 82]. Surface defects in carbon/graphene electrodes interact with the solvation shells of electrolyte ions, reducing the carbon‐surface‐ion separation distance and thereby increasing capacitance [83]. Research has shown that the distortion of ion solvation shells within small micropores significantly boosts capacitance by minimizing charge‐separation distance [84]. However, when pore sizes fall below the critical ion dimension, ion accessibility is restricted, and transport becomes sluggish. The high pore complexity impedes ion mobility, whereas aligned micropores enhance ion transport by up to three orders of magnitude [83].

In summary, to enhance energy storage performance, electrolytes must possess key properties such as strong ionic conductivity, high voltage stability, and corrosion resistance [21, 48, 79, 80]. Moreover, electrolyte‐electrode compatibility determines whether Al‐bearing species can reach active sites, undergo interfacial charge transfer, and cycle without parasitic reactions [81]. Detailed comparisons of aqueous, ionic‐liquid, DES, and gel/solid‐state electrolytes are provided in Section 4.

### 2.3 Mechanistic Insights from Al‐Ion Batteries: Suitable Storage Pathways for AICs

Advances in aluminum‐ion batteries (AIBs) may provide valuable understanding of the electrochemical processes that also govern AICs. Several charge‐storage pathways are relevant to Al‐based systems, including electrostatic adsorption, pseudocapacitive surface redox, intercalation of Al‐bearing species, and conversion reactions, which involve deeper structural rearrangements, or plating/stripping reactions. Each mechanism offers distinct advantages and challenges in terms of energy density, reversibility, and charge–discharge kinetics.

**FIGURE 4:**
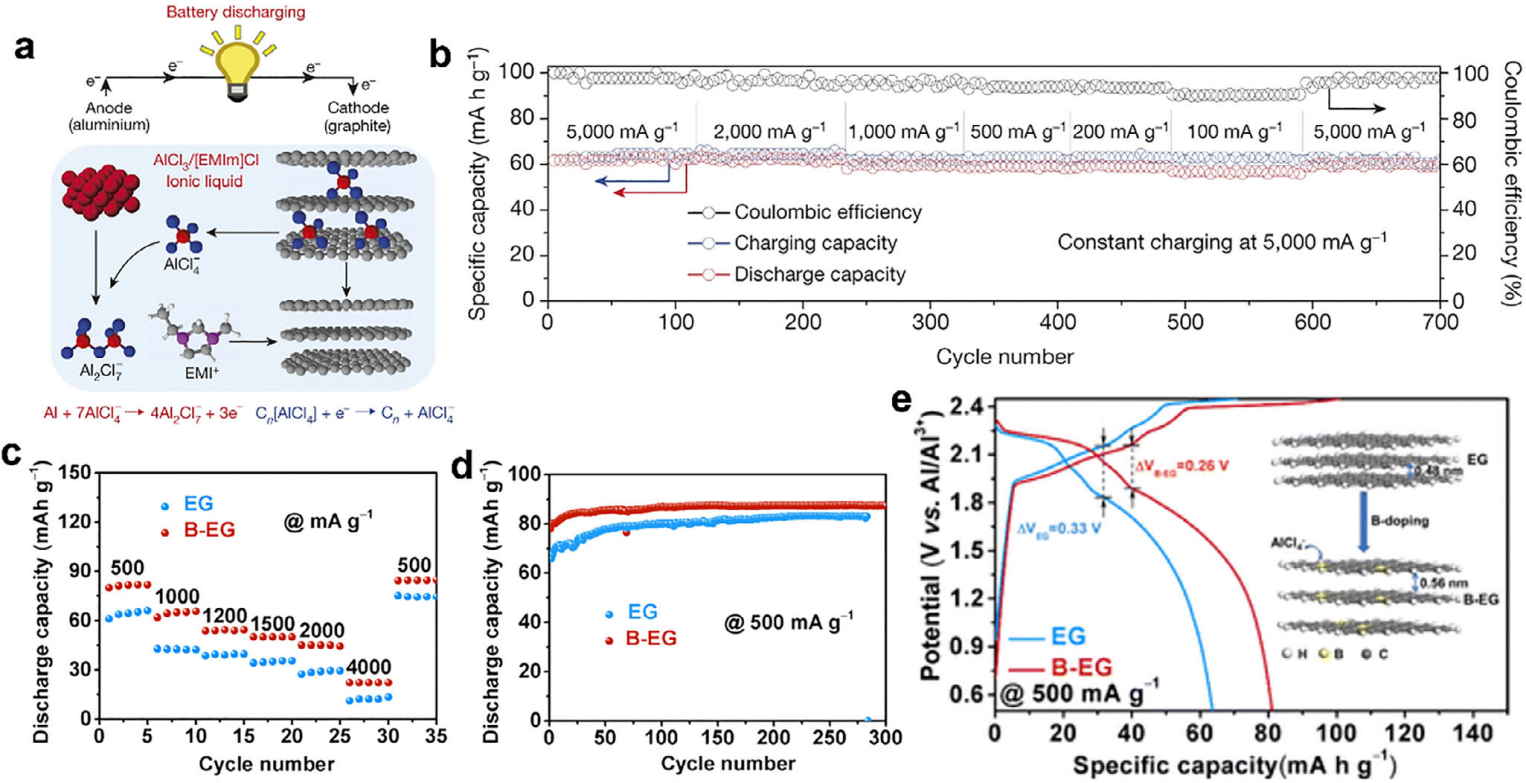
(a) Schematic drawing of the Al/graphite cell during discharge, using the optimal composition of the AlCl_3_/[EMIm]Cl ionic liquid electrolyte. On the anode side, metallic Al and ${\mathrm{AlCl}}_{4}^{-}$ were transformed into $Al_{2}{\mathrm{Cl}}_{7}^{-}$ during discharging, and the reverse reaction took place during charging. On the cathode side, predominantly ${\mathrm{AlCl}}_{4}^{-}$ was intercalated and deintercalated between graphite layers during charge and discharge reactions, respectively. (b) An Al/graphitic‐foam pouch cell charging at 5000 mA g^−1^ and discharging at current densities ranging from 100 to 5000 mA g^−1^. Reproduced from Ref. [85] with permission from Springer Nature, Copyright 2015. The electrochemical properties of EG and B‐EG: (c) rate capability, (d) cycle stability, and (e) charge–discharge curves of the second cycle at a current density of 500 mA g^−1^. Reproduced from Ref. [59] with permission from Royal Society of Chemistry, Copyright 2024.

In Al‐ion batteries, intercalation refers to the reversible insertion of chloroaluminate anions (*e.g*., $AlCl_{4}^{-}$) or other solvated Al^3+^ species into layered or open‐framework electrode hosts. Lin et al. showed that $AlCl_{4}^{-}$ can intercalate into graphite in an AlCl_3_/1‐ethyl‐3‐methylimidazolium chloride [EMIm]Cl ionic liquid electrolyte, achieving stable cycling and capacities around 66 mAh g^−1^ with good rate capability due to facile ion transport in the graphite lamellar channels (Figure 4a,b) [85]. However, such capacities are very low compared with Li‐ion intercalation into graphite. Building on this concept, boron‐doped expanded graphite (B‐EG) [59] enhances aluminum‐ion storage by increasing interlayer spacing and creating mesopores, which facilitate ion intercalation and adsorption. This structural modification reduces electrode pulverization and boosts pseudocapacitive contributions, leading to high specific capacity (84.9 mAh g^−1^ at 500 mA g^−1^) and excellent cycle stability (87.7 mAh g^−1^ after 300 cycles, Figure 4c–e).

**FIGURE 5:**
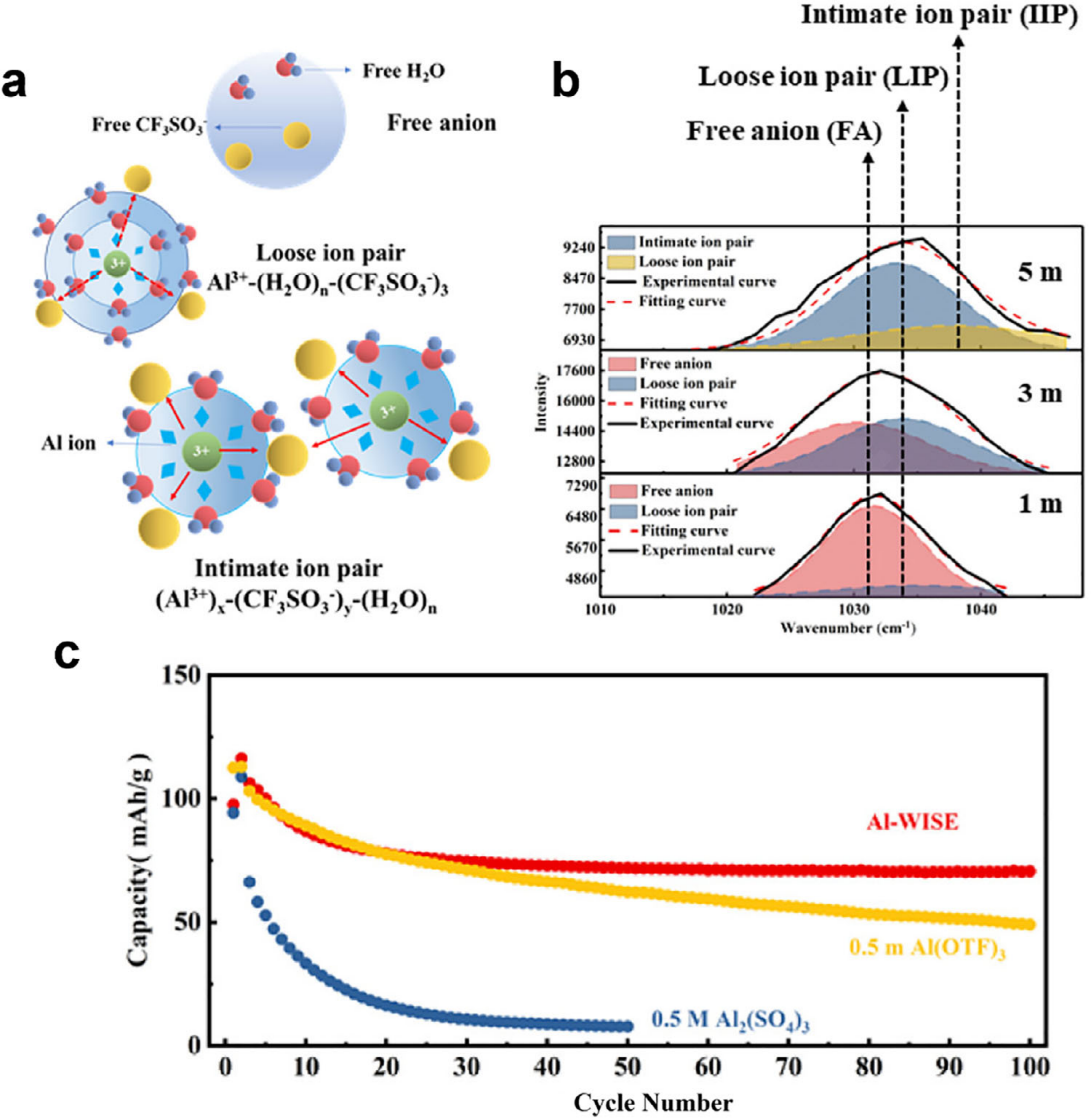
(a) The schematic diagram of the solvation structure. (b) Raman spectra of 1, 3, and 5 m Al(OTf)_3_ solution with the fitting of free anion, loose ion pair, and intimate ion pair. (c) Cycling life of the FeFe(CN)₆ Prussian blue analogue cathode cathode in Al‐WISE, 0.5 M Al_2_(SO_4_)_3_, and 0.5 m Al(OTf)_3_ electrolytes. Reproduced from Ref. [28] with permission from American Chemical Society, Copyright 2019.

In aqueous Al‐ion batteries, a FeFe(CN)_6_ cathode [28] paired with WISE delivered high capacity (116 mAh g*^−^* ^1^) together with moderate cycling stability (∼0.39% fading per cycle, Figure 5c). These improvements arise from the formation of a stable solid–electrolyte interphase (SEI), which enables reversible insertion of Al^3+^ species, while the WISE medium improves ionic conductivity and suppresses parasitic water decomposition and hydrogen evolution during Al^3+^ reduction at negative potentials. This study highlighted the critical role of WISE in enabling stable and efficient aqueous Al‐ion storage. Unlike dilute aqueous systems, where Al^3+^ has a strongly coordinated primary hydration shell and additional outer‐sphere water molecules [86] (Figure 5a), the limited number of free water molecules in concentrated electrolytes disrupts complete hydration. In highly concentrated Al(OTf)_3_ WISE (e.g., 5 M), Al^3+^ instead forms loose or intimate ion pairs with anions, creating a quasi‐salt‐like liquid environment often described as liquefied salt [28, 87, 88]. This restructuring of the solvation shell significantly impacts interfacial behavior. The reduced water activity suppresses side reactions such as hydrogen evolution and electrolyte decomposition, while stabilizing the electrode–electrolyte interface. Raman spectroscopy provides evidence for these changes, showing progressive blue shifts in SO_3_ vibrational modes with increasing concentrations (1, 3, and 5 m Al(OTf)_3_ in H_2_O), consistent with stronger cation–anion association and weakened hydration (Figure 5b).

Overall, these studies highlight that both structural engineering of electrode materials (e.g., boron doping, mesopores) and optimized electrode–electrolyte interactions (e.g., WISE) are crucial strategies to boost reversible intercalation, charge storage capacity, and cycling stability (Figure 5c). These intercalation systems are generally marked by sloping voltage profiles rather than sharp plateaus (Figure 4e), moderate but stable specific capacities, and relatively minor structural disruptions. Such features make intercalation hosts highly relevant to AICs, where rapid kinetics, good cyclability, and interface stability are essential. These characteristics indicate that the materials supporting reversible $AlCl_{4}^{-}$ intercalation (or similar anionic/solvated‐ion mechanisms) can serve as useful templates for designing AICs that retain high power and durability.

Pure conversion mechanisms are generally unfavorable for aqueous AICs due to several inherent limitations. These reactions involve significant structural changes, leading to large volume expansion and contraction during cycling, which can cause electrode pulverization and capacity fading. Moreover, conversion reactions often exhibit slow kinetics and large polarization, resulting in poor rate capability and limited cycling stability, which are critical drawbacks for high‐performance AICs. In contrast, intercalation‐based mechanisms offer a more favorable approach as discussed above, enabling reversible insertion and extraction of ions without extensive structural changes, thereby maintaining electrode integrity and enhancing cycling stability. Additionally, hybrid intercalation‐pseudocapacitive mechanisms, which combine the benefits of intercalation with surface‐controlled redox reactions, can further improve energy density and power delivery [89]. These mechanisms provide a balanced solution, bridging the gap between the high‐rate performance of supercapacitors and the high energy density of batteries, making them more suitable for AICs.

### 2.4 Challenges in Using Al^3+^ in Fast Kinetics Systems (Capacitors)

Despite aluminum's abundance, low cost, and high theoretical charge capacity, the application of Al^3+^ in AICs faces intrinsic barriers that are distinct from monovalent‐ion systems. These challenges arise from the trivalent ion's physicochemical properties, its solvation behavior, and the instability of electrode–electrolyte interfaces under high‐rate operation. The strong solvation of Al^3+^ leads to high desolvation energies and sluggish interfacial charge‐transfer kinetics.

**FIGURE 6:**
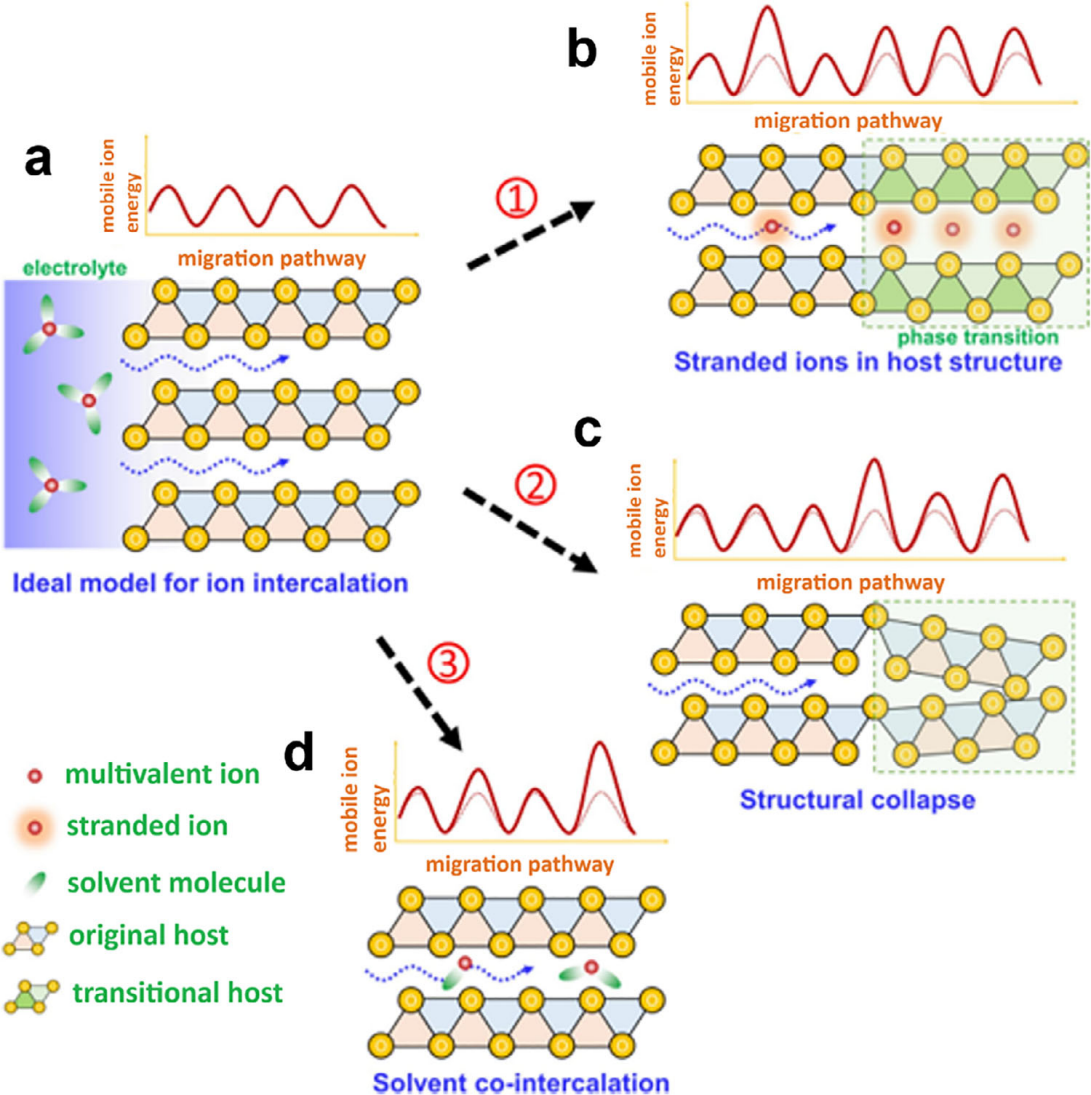
Illustration of challenges for multivalent‐ion diffusion in materials and electrodes. (a) Ideal model for ion intercalation into host structure. (b) Stranded ions in host structure that may occupy the original active sites and cause the phase transition. (c) Structural collapse caused by the multivalent‐ion diffusion. (d) Solvent co‐intercalation accompanied by the multivalent ions. Reproduced from Ref. [30] with permission from Elsevier, Copyright 2022.

In general, despite the small bare ionic radii, the high charge densities of multivalent ions often display in practice sluggish diffusion due to their large solvated or complexed forms. Strong electrostatic interactions between ions such as Al^3+^, Mg^2+^, and Zn^2+^ and the host lattice increase the diffusion barrier, causing partial ion trapping, structural distortion, or even irreversible phase transitions (Figure 6). These effects hinder charge transport, reduce active sites, and may trigger transition‐metal dissolution, ultimately compromising electrode stability and performance.

Furthermore, multivalent ions exhibit strong solvation in electrolytes, forming stable complexes (e.g., $AlCl_{4}^{-}$, MgCl^+^, $Zn{\left(H_{2}O\right)}_{x}^{2+}$) with solvent molecules [30, 90]. The tight binding in these solvated structures results in high desolvation energy and sluggish ion transfer at the electrode–electrolyte interface. The extent of solvation and ion‐pair formation differs significantly among aqueous, ionic‐liquid, and deep eutectic electrolytes, leading to distinct charge‐transfer and transport behaviors. In aqueous electrolytes, Al^3+^ predominantly exists as strongly hydrated complexes, such as [Al(H_2_O)_6_]^3+^, where the high hydration energy creates a substantial desolvation barrier before ion adsorption or insertion can occur. Although aqueous electrolytes generally possess high ionic conductivity and low viscosity, the strong coordination between Al^3+^ and water molecules can limit interfacial charge‐transfer kinetics. In contrast, chloroaluminate ionic liquids contain complex ions such as $AlCl_{4}^{-}$ and $Al_{2}{\mathrm{Cl}}_{7}^{-}$ rather than free Al^3+^, and charge storage is governed by the transport, adsorption, or intercalation of these species. The weaker coordination environment reduces desolvation constraints, but the higher viscosity of ionic liquids often results in slower mass transport and lower ionic conductivity. Deep eutectic electrolytes exhibit intermediate behavior, where extensive hydrogen‐bond networks and ion association modify both speciation and transport properties. Consequently, the electrochemical performance of AICs is determined by a balance among desolvation energetics, ion‐pair formation, electrolyte viscosity, and ionic mobility, highlighting the critical role of electrolyte engineering in optimizing Al‐ion storage. Although solvation can mitigate electrostatic repulsion, the enlarged ionic complexes experience substantial diffusion resistance within confined lattice structures, necessitating open frameworks and wider ion channels.

Overall, the disparity between theoretical capacity and experimental performance in multivalent‐ion storage mainly stems from factors such as strong ion‐lattice coupling, structural instability, and interfacial desolvation barriers. Addressing these issues requires engineering of diffusion environments, lattice stabilization, and interface optimization to enable efficient and reversible multivalent‐ion transport. Thus, one of the most critical challenges lies in the strong solvation and hydrolysis of Al^3+^ ions, which result in high desolvation energy and sluggish interfacial kinetics, as discussed in the following sub‐sections.

#### 2.4.1 Strong Solvation and Interfacial Kinetic Barriers of Al^3^ *^+^*

**FIGURE 7:**
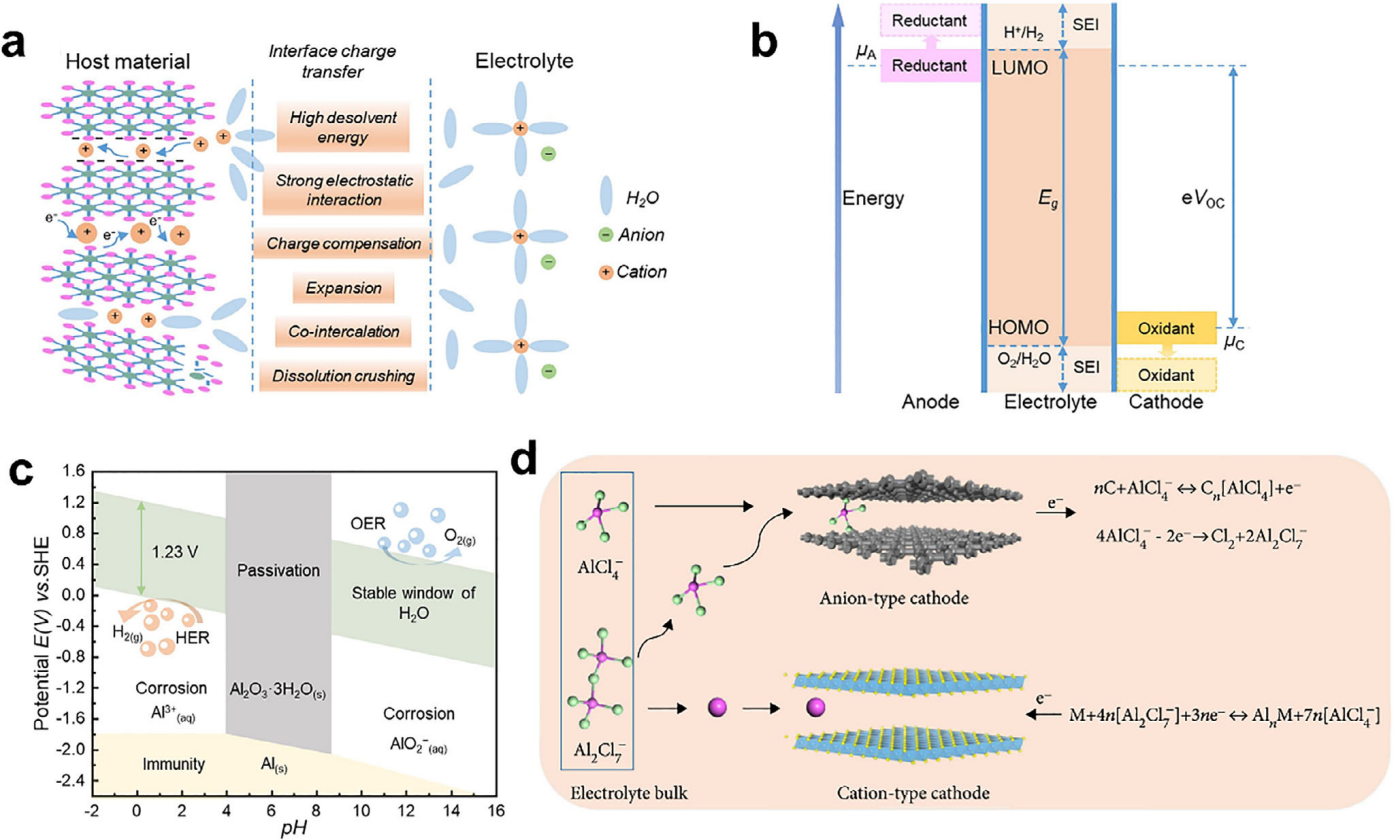
(a) A schematic diagram of the possible cathodic/anodic reaction process in an aqueous electrolyte. (b) Open circuit energy diagram of aqueous electrolyte. E_g_ is the window for thermodynamic stability of electrolytes. (c) Pourbaix diagram of Al in water at 25°C describes the basic oxidation/reduction reaction of Al in the water system. Reproduced from Ref. [29] with permission from Elsevier, Copyright 2024. (d) Schematic illustration of a possible cathode reaction process in the ionic liquid‐based electrolyte. Reproduced from Ref. [74] with permission from AAAS, Copyright 2021.

Building upon the transport limitations discussed above, the interfacial desolvation process represents one of the major kinetic bottlenecks for Al^3+^ storage. The trivalent cation exhibits strong coulombic interactions with surrounding solvent molecules, typically forming highly stable octahedral hydration shells in aqueous media or strongly coordinated complexes in non‐aqueous electrolytes [29, 91]. In addition, Al^3+^ readily undergoes hydrolysis, producing hydrolyzed species that further complicate the solvation environment [91]. As introduced above, the exceptionally high polarizing power of Al^3+^ (10.68 e Å^−2^) strengthens its electrostatic interactions with surrounding solvent molecules and anions, resulting in higher desolvation energies than those of monovalent and divalent ions and consequently slowing interfacial charge‐transfer kinetics [29, 92]. As a result, interfacial charge‐transfer kinetics are intrinsically slow, and the activation overpotentials become non‐negligible even at modest current densities (Figure 7a–c).

For supercapacitors, where ultrafast ion adsorption/desorption or surface/near surface redox reactions govern charge storage, the high desolvation energy of Al^3+^ severely impedes interfacial kinetics. This prolongs the characteristic time constants of charging and discharging, thereby reducing accessible capacitance at high rates and undermining the high‐power performance that distinguishes supercapacitors from batteries. This challenge underscores the importance of electrolyte design strategies aimed at tuning solvation structures, mitigating hydrolysis, or stabilizing more weakly coordinated Al species to lower the interfacial kinetic barrier.

#### 2.4.2 Ion Transport and Structural Compatibility of Electrode Materials

Beyond the interfacial desolvation process discussed above, the transport of Al‐bearing species within electrode materials presents an additional kinetic limitation. Even after partial desolvation, Al^3+^‐bearing charge carriers often exhibit intrinsically slow diffusion in both electrolytic media and solid‐state electrode hosts, primarily due to strong Coulombic interactions with counter‐ions, solvent molecules, and the ions of the lattice framework (Figure 6) [27, 29]. The intercalation of trivalent ions often induces significant lattice strain or irreversible structural modifications, thereby limiting the range of electrode materials that can sustain repeated cycling [29, 48]. In non‐aqueous systems, the large size of solvated Al^3+^ species and associated complex anions can impede their access to the microporous regions of carbon‐based electrodes, which are essential for achieving high double‐layer capacitance (Figure 7d) [61, 93]. In addition, side reactions and the limited compatibility of electrolytes with the cathode and binder materials are additional obstacles that hinder Al‐ion storage capability [74]. Collectively, these constraints result in slow ion dynamics, diminished capacitance retention at elevated current densities, and accelerated performance decay under rapid charge–discharge conditions.

Thus, AIC electrodes should have hierarchical porosity, expanded galleries, conductive scaffolds, and chemically stable interfaces. Electrolytes should be selected based not only on conductivity and voltage window, but also on their ability to form Al‐bearing species that are compatible with the pore size, surface chemistry, and redox mechanism of the electrode.

## 3 Electrode Materials for Advanced AICs

The electrochemical behavior of AICs is influenced by the structural and compositional design of electrode materials, which together with the electrolytes govern charge storage capability, ion transport dynamics, and cycling stability (as discussed in Section 2.4.2). The high charge density and relatively large hydrated radius of Al^3+^ demand materials with tailored porosity, robust electronic conductivity, and stable surface chemistry. Developing electrodes that balance these requirements while enabling rapid and reversible redox reactions remains a critical research frontier in advancing AICs. Various material design strategies have been explored to enhance energy storage performance. For example, pore‐size tuning can ensure ion accessibility and thereby improve EDL capacitance and rate performance. Thus, various carbon‐based materials, metal oxides/sulfides, organic molecules and MOFs have been developed for Al^3+^‐species charge storage.

### 3.1 Carbon‐Based Materials for Advanced AICs

#### 3.1.1 Porous Carbons

**FIGURE 8:**
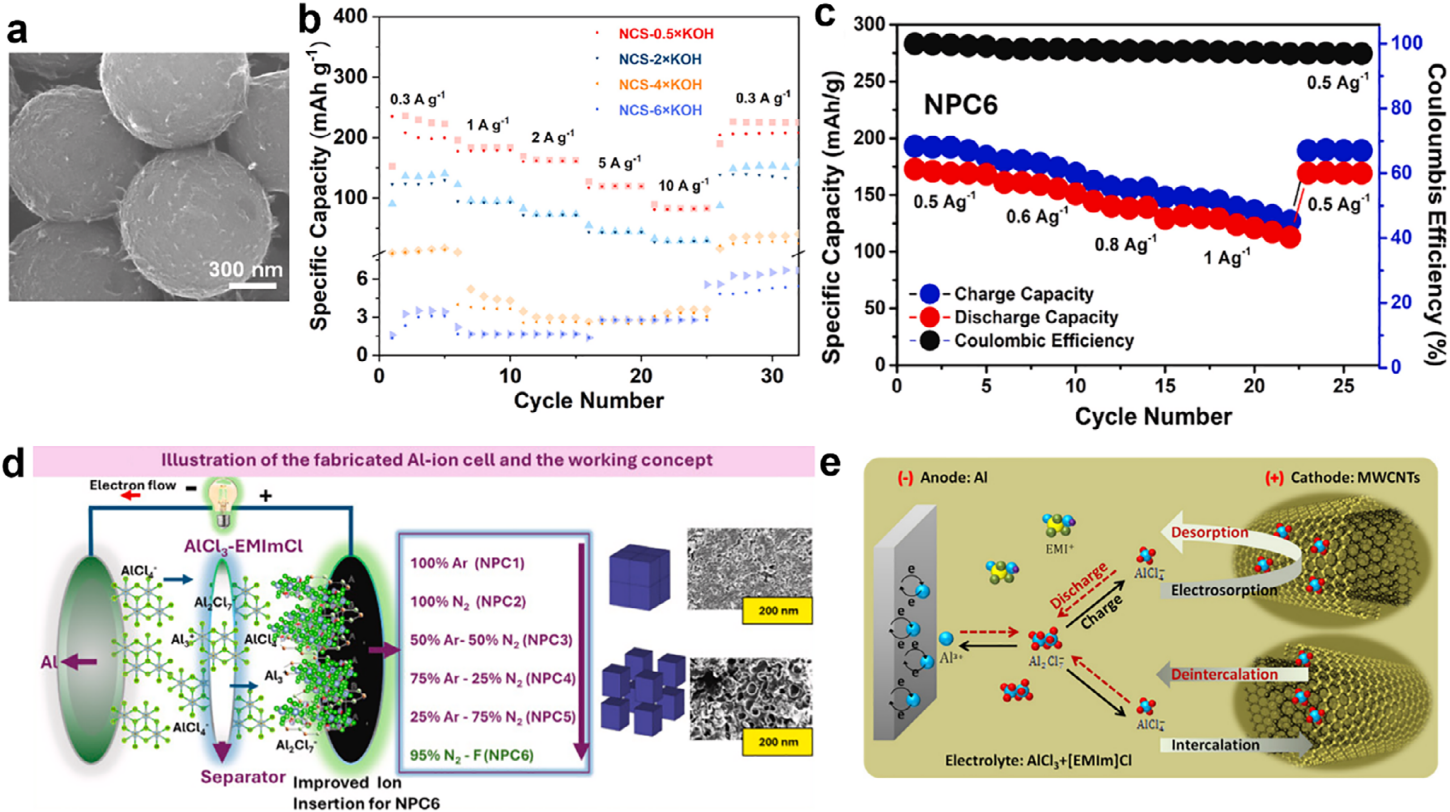
(a) Scanning electron microscopy (SEM) images of nitrogen‐doped micro‐mesoporous carbon sphere  (b) Rate capabilities of NCS with different activator content (a,b) (Reproduced from Ref. [55] with permission from Elsevier, Copyright 2023) (c) rate performance, and CE at 0.5 A g^−1^ (d) schematic representation of the fabricated Al‐ion cells, with the cell design structure, electrode compositions and highlights of the study (c,d) (Adapted from Ref. [69] with permission from Elsevier, Copyright 2025) (e) Energy‐storage mechanism of the MWCNTs in the ionic liquid electrolyte. Schematic illustration of the aluminum‐ion hybrid capacitor utilizing a MWCNTs electrode in an ionic liquid electrolyte. Reproduced from Ref. [94] with permission from Wiley‐VCH, Copyright 2016.

Porous carbons with large specific surface areas serve as pivotal components in supercapacitors, providing abundant ion‐accessible sites that enable ultrafast charge storage and accelerated electrochemical kinetics. Their intrinsic electrical conductivity, tunable pore architecture, and excellent compatibility with diverse aluminum‐based charge‐storage chemistries render them a versatile and effective platform for constructing high‐performance AICs. Consequently, nitrogen‐doped micro‐mesoporous carbon spheres (NCSs) [55] derived through KOH activation have demonstrated remarkable potential as cathode materials for AICs. The hierarchical micro/mesoporous framework enhances ion diffusion and charge accessibility, while nitrogen doping improves surface wettability and contributes additional pseudocapacitance. As a result, in a two‐electrode configuration these electrodes deliver high capacity (224 mAh g^−1^ at 0.3 A g^−1^), retain good rate capability (114 mAh g^−1^ at 10 A g^−1^, Figure 8a,b), and maintain nearly 100% capacity after 35,000 cycles, demonstrating how structural and chemical tuning can translate into exceptional performance in AICs. However, the absence of active‐material loading, areal capacity, and mass‐balancing data limits assessment of the device's practical scalability. Recent advances in heteroatom‐doped nanoporous carbons highlight both the promise and the persistent challenges of Al‐ion storage. N–F co‐doped carbons [69] derived from recycled polystyrene demonstrated remarkable structural properties (surface area ∼3048 m^2^ g^−1^, uniform mesopores) and the resulting Al|AlCl_3_‐[EMIm]Cl|nitrogen/fluorine co‐doped nanoporous carbon (NPC) device achieved high specific capacity (∼197 mAh g^−1^ at 0.5 A g^−1^) with ∼97% CE, underscoring the potential of rational pore and surface chemistry engineering to boost ion adsorption and pseudocapacitive contributions (Figure 8c,d). The reported active mass loading is 0.8–2 mg per electrode (∼1.7–2.2 mg cm^−2^) with cathode thickness 18 µm. However, cycling stability remains a major limitation, with capacity fading observed within 25–50 cycles. Post‐cycling field‐emission scanning electron microscopy and energy‐dispersive X‐ray spectroscopy (EDS) and Raman analyses reveal carbon dissolution in the electrolyte, peeling of carbon layers, and accumulation of Al‐, Cl‐, and O‐containing species, alongside chemical transformations such as C–Cl, C–F, and C = N bond degradation. These interfacial instabilities hinder ion transport and progressively deactivate active sites, ultimately shortening cycle life. Future advancement requires optimizing electrolytes with stabilizing additives, engineering robust pore architectures, and applying protective surface modifications to suppress side reactions and extend cycle life.

An early demonstration of an aluminum‐ion hybrid capacitor employed multi‐walled carbon nanotubes (MWCNTs) as the cathode material in a chloroaluminate [EMIm]Cl‐based ionic liquid electrolyte (Figure 8e) [94]. The device exhibited a dual charge‐storage mechanism: (i) electrochemical double‐layer capacitance arising from the reversible adsorption/desorption of $AlCl_{4}^{-}$ anions on the MWCNT surface, and (ii) a limited pseudocapacitive contribution from the intercalation/deintercalation of $AlCl_{4}^{-}$ into graphitic domains, particularly evident at low current densities. Three‐electrode GCD measurements confirmed the expected decline in specific capacitance with increasing current density, consistent with the gradual suppression of diffusion‐driven intercalation processes under high‐rate operation. Despite this trade‐off, the device maintained 82.4% capacitance retention after 3,500 cycles at 0.4 A g^−1^, highlighting its reasonable cycling stability. However, the reported performance was obtained at a relatively low active‐material loading (∼0.8 mg per electrode on a 6 mm diameter electrode), and validation under higher mass loadings is required to assess practical device performance and scalability.

An Al‐HC in a two‐electrode configuration using pore‐size‐controlled activated carbon (AC) cathode, Al foil anode, and an AlCl_3_‐based ionic liquid exhibits high gravimetric and volumetric energy densities of 51 Wh kg^−1^ and 28 mWh cm^−3^, and power densities of 91 W kg^−1^ and 50 mW cm^−3^ comparable to Li‐HCs tested in that study [95]. In this study, starch‐derived activated carbon (AC), optimized for Al complex ion transport, enables fast charge/discharge, long‐term stability (97.9% capacitance retention over 10 000 cycles), and high CE (97.6–99.9%). Notably, the Al‐HC was evaluated at practical electrode loadings of 3.9 mg (1.53 mg cm^−2^) for the AC cathode and 1.6 mg (0.63 mg cm^−2^) for the Al anode, with device metrics calculated using the total mass of both electrodes. Furthermore, a green, solvent‐free approach has been developed for the facile and scalable synthesis of m‐phenylenediamine‐formaldehyde resin spheres, which can be converted into porous carbon spheres (PCSs) via carbonization and activation [33]. This method achieves ultrahigh productivity and a high polymer yield of 98.9%, with PCSs retaining the spherical morphology and achieving a carbonization yield of 14.5%. When employed as cathodes in Al‐HC using two‐electrode configuration, these PCSs deliver exceptional capacity of ∼200 mAh g^−1^ (based on cathode mass with average loading ∼1.5 mg cm^−2^), among the highest reported for porous carbon in Al‐based energy storage devices and maintain a stable capacity of 150 mAh g^−1^ at 2000 mA g^−1^ over 1500 cycles. The resulting Al‐HC exhibits a maximum energy density of 220 Wh kg^−1^ and a power density of 9 kW kg^−1^, along with minute‐level charge/discharge capability. This high performance is attributed to the uniform porous structure, high surface area, and robust spherical morphology of the PCSs, which facilitate fast ion transport and efficient charge storage. This solvent‐free strategy not only enables mass production of high‐performance carbon electrodes but also holds promise for other metal‐ion HCs, including magnesium‐ and calcium‐ion systems. Overall, these studies establish porous carbon as a promising and foundational platform for AICs, emphasizing the critical role of rational structural and chemical tuning to achieve high charge storage capability, while addressing persistent cycling stability challenges.

#### 3.1.2 2D Materials for Advanced AICs

**FIGURE 9:**
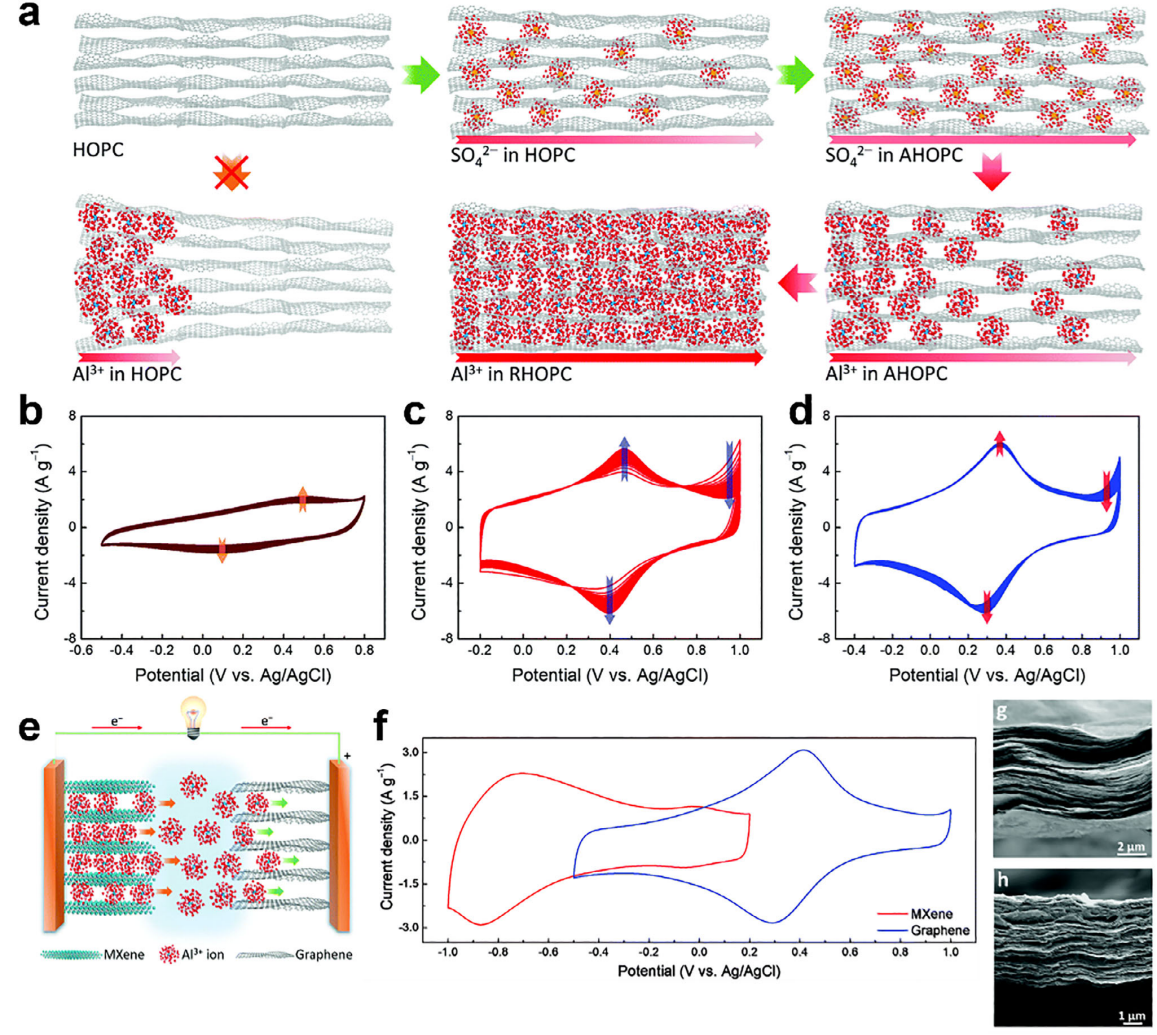
Pore‐structure remolding of the graphene‐based cathode. (a) Schematic illustration of the self‐adaptive electrochemical pore‐structure remolding process. (b) The first twenty cyclic voltametry (CV) curves of HOPC in the Al_2_(SO_4_)_3_ electrolyte. (c) The first twenty CV curves of HOPC in the H_2_SO_4_ electrolyte, producing electrochemically activated HOPC (AHOPC). (d) The first twenty CV curves of AHOPC in the Al_2_(SO_4_)_3_ electrolyte, producing completely remolded AHOPC (RHOPC). (e) Schematic illustration of the aqueous rocking‐chair aluminum‐ion capacitor. (f) Typical cyclic voltammetry curves of the graphene‐based cathode and the MXene‐based anode. (g) Ti_3_C_2_T_x_ film and (h) alkali‐treated Ti_3_C_2_T_x_ film. Reproduced from Ref. [27] with permission from Royal Society of Chemistry, Copyright 2022.

As discussed in Section 2.4, recent studies have demonstrated that the large effective size and very high hydration enthalpy of Al^3+^ (0.475 nm, 4525 kJ mol^−1^) severely limit the ability of hydrated Al‐bearing species to access small pores, which constrains charge storage in microporous electrodes [27]. To overcome this, a two‐step electrochemical activation strategy has been developed, wherein intermediate‐sized “pillar” ions, such as SO_4_^2−^, are first used to pre‐remodel the pore structure of highly ordered and compact porous carbon (HOPC) through a template effect. This pre‐remodeling enlarges characteristic pores under strong electric fields, creating a framework that can later accommodate hydrated Al^3+^ ions efficiently (Figure 9). Upon subsequent cycling in Al^3+^ containing electrolytes, these electrochemically activated HOPC electrodes (AHOPC) undergo further adaptive remolding, eventually achieving a fully optimized remolded activated HOPC (RHOPC) structure that maximizes pore accessibility for hydrated Al^3+^ ions. This approach simultaneously enhances specific surface area, enlarges pore size, and introduces electrochemically active >C═O functional groups, which contribute additional redox pseudocapacitance [27, 96]. Consequently, the charge storage mechanism of RHOPC transitions to a redox‐dominated pseudocapacitive process, as evidenced by the appearance of distinct redox peaks in cyclic voltammetry. This method enabled the construction of an aqueous asymmetric AIC employing remolded graphene and MXene electrodes (mass loading ∼2 mg cm^−2^) with Al_2_(SO_4_)_3_ electrolyte. The device achieved a wide operating voltage (2.0 V), high volumetric capacitance (up to 945 F cm^−3^), an energy density of 112 Wh L^−1^, and excellent cycling stability, underscoring the promise of pore‐engineered materials for high‐performance AICs.

**FIGURE 10:**
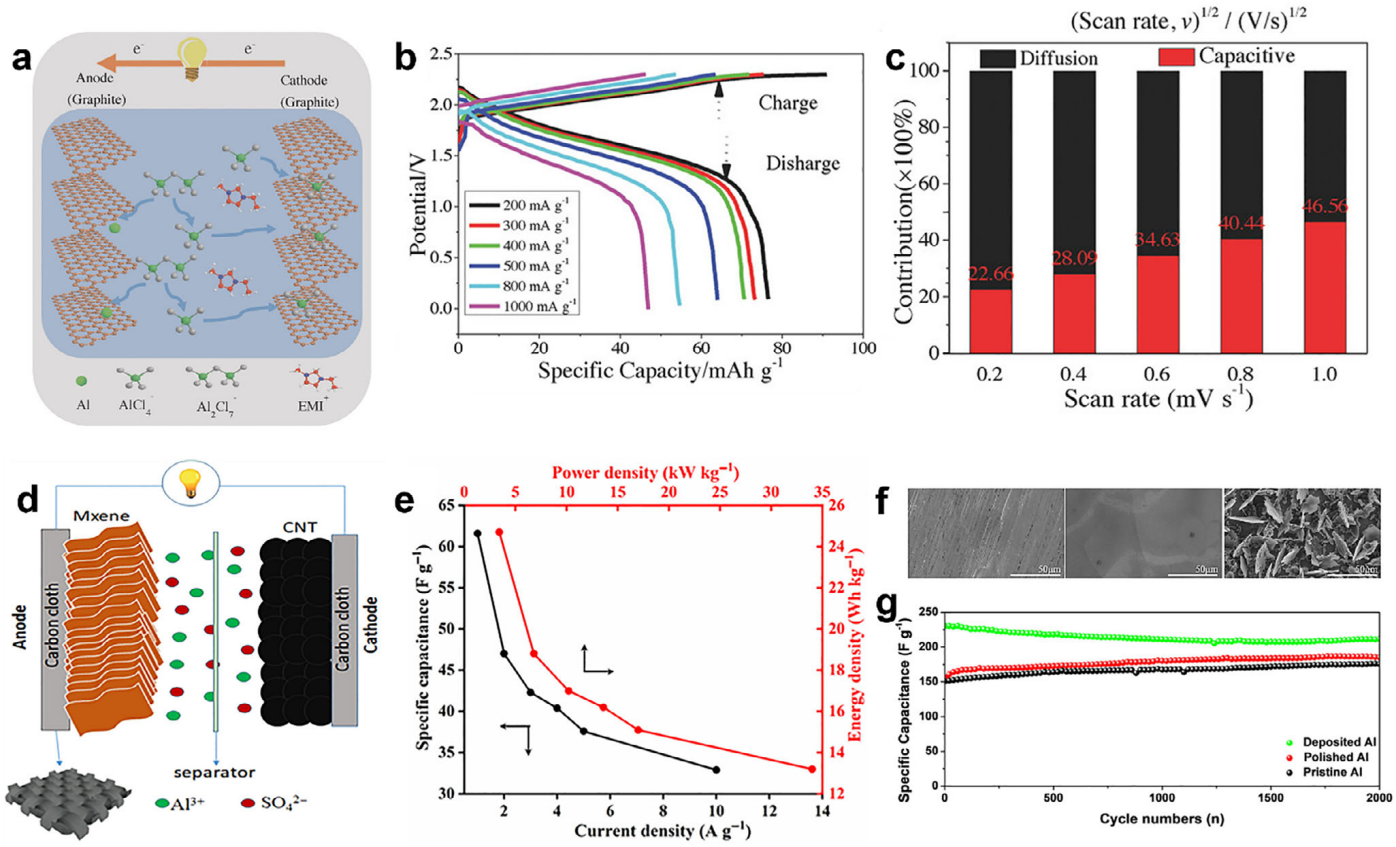
(a) Schematic illustration of the charging mechanism of the GGDIB based on AlCl_3_/[EMIm]Cl ionic liquid electrolyte. (b) Charging and discharging curves at different current densities (c) the relationship plots between I(V)/v^1/2^ and v^1/2^ at different voltages, the ratio of the pseudocapacitive contribution at different sweep rates. (a–c) Reproduced from Ref. [97] with permission from Wiley‐VCH, Copyright 2018. (d) A schematic representation of Nb@CC//CNT@CC (Nb_2_CT_x_ on carbon cloth (Nb@CC)//carbon nanotubes on carbon cloth (CNT@CC)). (e) C_s_ versus current density (black) and Ragone plot (E*_d_* vs. P*_d_*) of asymmetric Nb@CC//CNT@CC cell (red). (d,e) Reproduced from Ref. [32] with permission from John Wiley & Sons Ltd., Copyright 2024. (f) SEM images of (i) pristine, (ii) electropolished, and (iii) electrodeposited aluminum anodes (scale 50 µm) (g) Galvanostatic cycling stability at 3 A g^−1^ of pristine, electropolished, and electrodeposited asymmetric supercapacitors. (f,g) Reproduced from Ref. [98] with permission from American Chemical Society, Copyright 2022.

A promising development is the aluminum‐based graphite–graphite dual‐ion battery (GGDIB), which employs AlCl_3_/[EMIm]Cl as a room‐temperature ionic liquid electrolyte and conductive graphite paper as both electrodes (Figure 10a) [97]. Although this graphite–graphite system is classified as an aluminum‐based dual‐ion battery rather than an AIC, it provides valuable mechanistic insights into chloroaluminate‐ion transport, ${\mathrm{AlCl}}_{4}^{-}$ intercalation behavior, pseudocapacitive contributions, and electrolyte–electrode interactions in AlCl_3_/[EMIm]Cl electrolytes. These findings are highly relevant for the rational design of carbon‐based cathodes and the optimization of non‐aqueous aluminum‐ion capacitors. In this system, aluminum undergoes reversible plating/stripping at the anode, while chloroaluminate anions are intercalated into the graphite cathode. The ionic liquid medium provides a wide electrochemical stability window (ESW, 0.1–2.3 V), and suppresses side reactions, resulting in an initial discharge capacity of 76.5 mAh g^−1^ (based on the mass of graphite cathode) at 200 mA g^−1^ and 62.3 mAh g^−1^ after 1,000 cycles with 98.4% capacity retention. Interestingly, the galvanostatic charge–discharge profiles exhibit sloping characteristics rather than distinct voltage plateaus, suggesting that the charge storage behavior lies at the intersection of battery‐type intercalation and capacitive processes. Thus, while often described as a “dual‐ion battery,” the electrochemical response bears strong resemblance to hybrid capacitor systems, offering a balance of high‐rate capability and long cycle life under the reported test conditions. Electrochemical analysis of the graphite–graphite dual‐ion configuration reveals a significant pseudocapacitive contribution to its charge storage behavior. At a scan rate of 0.8 mV s^−1^, pseudocapacitive processes account for ∼40% of the total capacity, increasing from ∼23% at 0.2 mV s^−1^ to nearly 47% at 1 mV s^−1^ (Figure 10b,c). This increasing fraction with sweep rate indicates that surface‐controlled redox reactions become more dominant under faster kinetics, while diffusion‐limited intercalation of $AlCl_{4}^{-}$ within the graphite lattice prevails at slower rates. Such mixed charge‐storage behavior highlights the hybrid nature of the system, bridging capacitive and intercalation mechanisms. Compared with conventional AICs based solely on porous carbon electrodes, the stronger pseudocapacitive response in the GGDIB enables superior rate performance and stable cycling, underscoring its potential as a high‐power energy storage device.

MXenes have recently gained attention as promising electrode materials for multivalent‐ion supercapacitors due to their high conductivity, tunable surface terminations, and layered structures that facilitate ion intercalation. Nb_2_CT_x_, derived from the selective etching of the Nb_2_AlC MAX phase and subsequent delamination, provides a porous, few‐layered morphology with large surface area and accessible electroactive sites [32]. When integrated onto carbon cloth substrates, Nb_2_CT_x_ was employed in both symmetric (Nb_2_CT_x_//Nb_2_CT_x_) and asymmetric (Nb_2_CT_x_//CNT) aluminum‐ion supercapacitor configurations with mass loadings of ∼1 mg cm^−2^. The Nb@CC//CNT@CC asymmetric AIC achieved a high energy density of 24.7 Wh kg^−1^ at 3.4 kW kg^−1^, retained 13.2 Wh kg^−1^ at 34.2 kW kg^−1^, and exhibited excellent cycling stability with ∼90% capacitance retention and 99% CE over 4000 cycles (Figure 10d,e). These devices demonstrated notable capacitance, favorable energy and power densities, and long‐term cycling stability, underscoring Nb_2_CT_x_ MXene as an efficient and versatile electrode candidate for Al‐ion storage.

In addition, a study demonstrated a high‐performance aluminum–graphene asymmetric AIC employing surface‐engineered aluminum anodes to enhance interfacial charge transfer and mitigate anode degradation [98]. Two surface‐modification methods, namely electropolishing and electrodeposition were compared (Figure 10f). Electropolishing effectively removed the passivating oxide layer (Al_2_O_3_), improving contact with the ionic liquid electrolyte (EMImCl–AlCl_3_) and lowering charge‐transfer resistance. However, the electrodeposited aluminum anode, characterized by a dendritic, leaf‐like morphology and strong adhesion to the molybdenum substrate, offered a substantially higher electrochemically active surface area and a more reactive interfacial surface for chloroaluminate‐mediated deposition/dissolution. These structural and interfacial features facilitated faster chloroaluminate‑complex ion transport and reversible Al deposition/dissolution, resulting in an outstanding specific capacitance of 211 F g^−1^, an energy density of 151 Wh kg^−1^, and a power density up to 11 104 W kg^−1^. Furthermore, the electrodeposited asymmetric AIC retained 87% capacitance after 5000 cycles, surpassing both pristine and electropolished counterparts (Figure 10g). The superior performance was mainly attributed to the enhanced surface roughness, reduced interfacial resistance, and high‐density active sites provided by the electrodeposited aluminum, which collectively improved ion accessibility and charge‐transfer kinetics. However, very low mass loading of the graphene cathode (0.17–0.24 mg cm^−2^) hinders the practical acceptance of the device.

Overall, the rapid evolution of aluminum‐based electrochemical energy storage highlights a clear shift toward mechanism‐driven electrode design. Strategic pore remolding, surface chemistry tuning, and the incorporation of conductive 2D materials such as MXenes have enabled enhanced ion transport, increased pseudocapacitive contributions, and improved structural stability. Meanwhile, dual‐ion configurations employing ionic liquid electrolytes demonstrate that aluminum systems can bridge the conventional boundaries between batteries and supercapacitors by combining intercalation chemistry with fast surface‐controlled kinetics. Moving forward, deeper mechanistic understanding, precise control over electrode/electrolyte interfaces, and scalable high‐mass‐loading architectures will be crucial for translating laboratory‐scale breakthroughs into commercially viable, high‐energy, and high‐power aluminum‐based storage technologies.

### 3.2 Metal Oxides and Composites

Metal oxides and hydroxides are widely explored as electrode materials for supercapacitors due to their high theoretical capacitance and multiple accessible redox states, which enable reversible faradaic reactions and pseudocapacitive energy storage [19]. Two‐dimensional oxides, in particular, offer atomically thin layers that maximize electrolyte contact and shorten ion diffusion pathways, enhancing charge storage kinetics.

**FIGURE 11:**
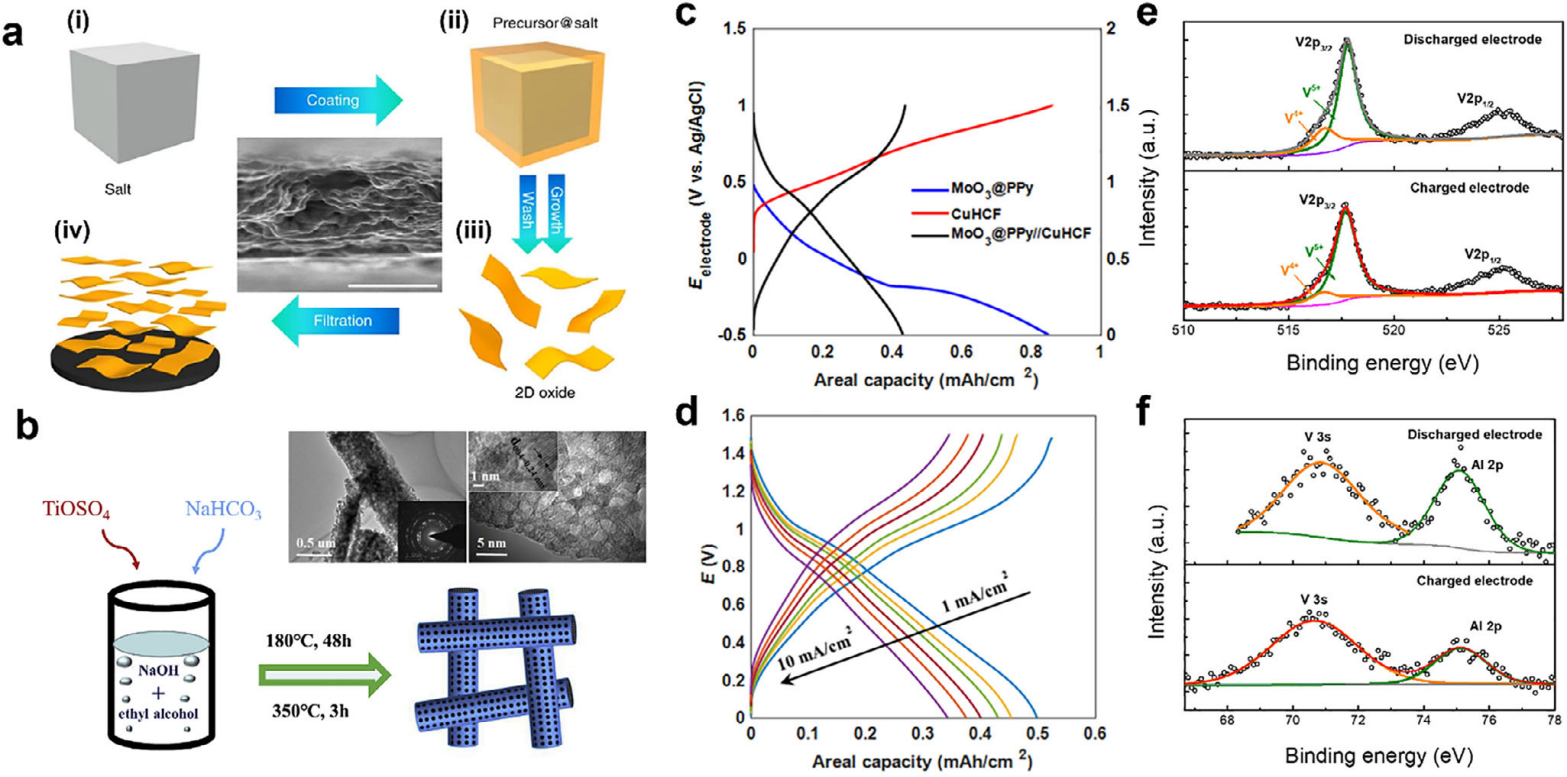
(a‐i, ii) Schematic representation of the fabrication process. The precursor solution was coated on the salt by mixing the solution with a large amount of salt microcrystals. (iii) After thermal annealing and rinsing in water, 2D oxide flakes were obtained. (iv) Pseudocapacitor electrodes were fabricated by filtering the 2D oxide/ethanol dispersion using a Celgard separator. (v) Multilayered 2D oxide is shown in the cross sectional SEM image. Scale bar, 1 µm. (Adapted from Ref. [80] with permission from Nature Portfolio, Copyright 2016), (b) Schematic synthesis process of the P‐TiO_2_‐MRs, transmission electron microscopy (TEM) and high‐resolution TEM images of P‐TiO_2_‐MRs. The insets are SAED pattern and enlarged HRTEM image. (Adapted from Ref. [70] with permission from Elsevier, Copyright 2020.) Electrochemical assessment of α‐MoO_3_@PPy//CuHCF Al^3+^‐ion pseudocapacitor: (c) *E* vs. capacity plots of individual α‐MoO_3_@PPy and CuHCF electrodes and α‐MoO_3_@PPy//CuHCF full cell recorded at a current density of 3 mA cm^−2^, (d) GCD profiles recorded at different current densities (Reproduced from Ref. [31] with permission from Elsevier, Copyright 2022.) (e) V 2p and (f) Al 2p X‐ray photoelectron spectroscopy (XPS) analyses of discharged and charged positive electrode of MCM/V_2_O_5%_–40%. The transformation between V^5+^ and V^4+^ and the intensity variation of the Al 2p peak indicate redox reactions during the charging/discharging process. (Reproduced from Ref. [71] with permission from American Chemical Society, Copyright 2019).

Hexagonal MoO_3_ (h‐MoO_3_) [80] synthesized using a combined vacuum‐based and solution‐based salt‐template strategy forms large nanosheets that can be assembled into binder‐free films (Figure 11a). This restacked architecture ensures rapid transport of hydrated or partially desolvated Al‐bearing species and maximizes the utilization of pseudocapacitive sites. As a result, these films achieve volumetric capacitances of approximately 300 F cm^−3^ at 5 mV s^−1^ in Al_2_(SO_4_)_3_ electrolyte and approach the theoretical capacitance predicted for h‐MoO_3_ in organic electrolytes at lower scan rates. The reported pseudocapacitive performance was obtained in a three‐electrode configuration using ultrathin (∼1 µm) h‐MoO_3_ electrodes (∼1.1 g cm^−3^), corresponding to an estimated active‐material loading of only ∼0.11 mg cm^−2^. Therefore, further validation in practical two‐electrode configuration with higher mass loadings is required to assess device‐level applicability. In another approach, porous TiO_2_ microrods (P‐TiO_2_‐MRs) [70] with uniform, large mesopores have been synthesized via a simple hydrothermal route and thermal treatment, yielding micro/nano‐structured anodes with high tap density and structural stability (Figure 11b). The porous architecture enhances electrode–electrolyte contact, facilitating fast Al^3+^ ion transport and intercalation, which results in a high discharge capacity of 111.1 mAh g^−1^ at 1 A g^−1^ and excellent rate capability of 49.8 mAh g^−1^ at 5 A g^−1^ observed in a three‐electrode configuration. Notably, an asymmetric AIC consisting of an insertion‐type P‐TiO_2_‐MRs anode and an activated carbon cathode (P‐TiO_2_‐MRs//AC, with a cathode/anode weight ratio of ∼1.3:1) was demonstrated as an aqueous asymmetric AIC. The device delivered an energy density of 26.3 Wh kg^−1^ at a power density of 627 W kg^−1^, retained 10.9 Wh kg^−1^ at a high power density of 5.45 kW kg^−1^, and maintained 63% capacity after 1,000 cycles. This work highlights the potential of mesoporous TiO_2_ microrods for high‐performance Al‐ion energy storage devices with both battery‐type and capacitive characteristics. A binder‐free, polypyrrole‐stabilized α‐MoO_3_ electrode (α‐MoO_3_@PPy) [31] fabricated via electrodeposition demonstrated high performance as an Al‐ion pseudocapacitor. The α‐MoO_3_@PPy//copper hexacyanoferrate (CuHCF) full cell, assembled with charge‐balanced electrodes (q^+^ = q^−^), delivered non‐linear galvanostatic charge–discharge (GCD) curves characteristic of pseudocapacitors and exhibited a high CE (∼98%). The device achieved areal discharge capacities of 0.50 and 0.34 mAh cm^−2^ at 1 mA cm^−2^ and 10 mA cm^−2^, retaining 68% capacity at high rates, with an energy efficiency of ∼71% at 3 mA cm^−2^ (Figure 11c,d). With a high mass loading (∼16 mg cm^−2^), the electrode retains 88% of its capacity after 1,000 cycles in 1 M AlCl_3_ electrolyte. A full cell with copper hexacyanoferrate as the cathode delivers energy densities of 0.33 and 0.20 mWh cm^−2^ at 1 and 10 mA cm^−2^, respectively, while maintaining 70% capacity over 1800 cycles, with performance normalized to the total surface area of both the anode and cathode. The combination of a binder‐free design and PPy‐mediated structural stabilization enables rapid Al^3+^ transport and long‐term cycling stability.

Mesoporous carbons have proven highly effective as scaffolds for hosting active species in multivalent‐ion supercapacitors, owing to their large surface areas and interconnected pore networks that facilitate ion transport. An illustrative example is the incorporation of V_2_O_5_ into mesoporous carbon microspheres (MCMs) [71], where the uniform pore channels allowed homogeneous V_2_O_5_ distribution and minimized particle agglomeration. This architecture combined the rapid charge storage of electric double‐layer capacitance from the carbon host with the redox‐driven pseudocapacitance of V_2_O_5_, thereby enhancing overall storage capability for Al^3+^ ions. The resulting symmetric two‐electrode configuration of MCM/V_2_O_5_ electrodes (mass loading 6.1 mg cm^−2^) exhibited an energy density of ∼18 Wh kg^−1^ at a power density of 147 W kg^−1^ and retained 88% of their capacity after 10 000 charge–discharge cycles in Al_2_(SO_4_)_3_ electrolyte. Electrochemical and spectroscopic analyses of this pseudocapacitive AIC further verified the reversible intercalation of Al^3+^ within the composite (Figure 11e,f).

An aqueous asymmetric AIC has been reported using polypyrrole‐coated MoO_3_ nanotubes (PPy@MoO_3_) as the anode and activated carbon (AC) as the cathode in Al_2_(SO_4_)_3_ electrolyte (mass ratio of the anode to the cathode was designed to be 3.1 : 1) [67]. The device operates via redox‐driven intercalation/deintercalation of Al^3+^ ions into MoO_3_, as confirmed by cyclic voltammetry, ex situ X‐ray diffraction, X‐ray photoelectron spectroscopy, elemental mapping, and first‐principles calculations. The PPy coating enhances electrical conductivity and structural stability, resulting in a high specific capacitance of 693 F g^−1^, approximately three times higher than that of typical sodium‐ion capacitor electrodes in an aqueous electrolyte. The AIC delivers an energy density of 30 W h kg^−1^ and maintains 93% of its capacitance after 1,800 cycles, demonstrating excellent cycling stability and rate capability. Similarly, a freestanding composite electrode composed of W_18_O_49_ nanowires (W_18_O_49_NWs) [66] and single‐walled carbon nanotubes (SWCNTs) demonstrated outstanding Al^3+^ storage performance. The W_18_O_49_NWs possess a layered single‐crystal structure, high aspect ratio, and wide lattice spacing, facilitating efficient Al^3+^ intercalation. The interpenetrating nanonetwork with SWCNTs ensures fast ion transport and high electrical conductivity, thereby enhancing the overall electrochemical performance and cycling stability of the electrode. The resulting asymmetric AIC exhibited a high volumetric energy density of 19.0 mWh cm^−3^ at 295 mW cm^−3^, with relatively balanced electrode masses of 5.93 mg for the positive electrode and 6.26 mg for the negative electrode.

In summary, metal oxides and their carbon‐based composites have demonstrated great promise as active materials for AICs, offering a balanced combination of high energy density, fast ion transport, and long‐term cycling stability. The integration of redox‐active oxides with conductive porous carbons or nanotube networks effectively mitigates the intrinsic conductivity and structural limitations of pure oxides. These synergistic architectures enable efficient Al^3+^ intercalation and robust charge storage behavior, underscoring their potential as key building blocks for next‐generation, high‐performance AICs.

### 3.3 Other Electrode Materials

Emerging electrode families such as conductive polymers, MOFs, covalent organic frameworks (COFs), and organic redox‐active materials represent promising yet underexplored directions for AIC development. Their highly tunable chemical structures, abundant redox‐active sites, and potential for flexible device integration may offer new opportunities to overcome the diffusion limitations associated with multivalent charge carriers. In particular, MOF‐ and COF‐derived porous architectures provide controllable pore networks and surface functionalities that could facilitate Al‐species transport, while conductive polymers and organic redox systems may enable lightweight and mechanically flexible energy‐storage devices. Nevertheless, the long‐term stability, electrical conductivity, and practical charge‐storage mechanisms of these materials in Al‐based electrolytes remain insufficiently understood, highlighting the need for further systematic investigation.

Beyond carbon‐based materials (such as porous carbon, graphene, and MXenes) and transition‐metal oxides/hydroxides, Prussian blue analogues (PBAs) have emerged as promising electrode materials for Al‐HCs. Owing to their three‐dimensional open framework, high structural stability, large specific surface area, and uniformly distributed ion channels, PBAs facilitate efficient Al^3+^ ion intercalation/deintercalation during electrochemical cycling. Chiang et al. synthesized K_0.02_Cu[Fe(CN)_6_]_0.7_·3.7H_2_O PBA (CuFe‐PBA) via a simple room‐temperature co‐precipitation method, in which aqueous Cu^2+^ and [Fe(CN)_6_]^3−^ precursors were simultaneously combined to induce immediate formation of CuFe‐PBA precipitate. The as‐prepared CuFe‐PBA was then employed as a cathode material in a three‐electrode open cell configuration using 1 M Al(NO_3_)_3_ as an electrolyte [64]. The CuFe‐PBA electrode demonstrated reversible Al^3+^ ion intercalation, delivering a specific capacity of 55 mAh g^−1^ at ∼1C, corresponding to a ∼0.6 e^−^ transfer or 0.2 Al^3+^ intercalation/deintercalation per formula unit (Al_0.2_CuFe‐PBA). Ex situ X‐ray diffraction (XRD) analysis revealed systematic shifts of the (400), (420), and (422) diffraction peaks toward higher angles with increasing Al content and lower angles upon deintercalation, confirming the reversible intercalation of Al^3+^ ions within the CuFe‐PBA framework. Furthermore, a full‐cell device was assembled with Al_0.2_CuFe‐PBA as the cathode and activated carbon (AC) as the anode, with active‐material loadings of 2.45 and 10.28 mg, respectively, over an electrode area of 0.5 cm^2^. The device achieved an energy density of 13 Wh kg^−1^, excellent cycling stability (90% capacity retention), and a high CE of 99.1% after 1,000 cycles, underscoring the potential of PBAs as robust cathode materials for next‐generation Al‐HCs.

**FIGURE 12:**
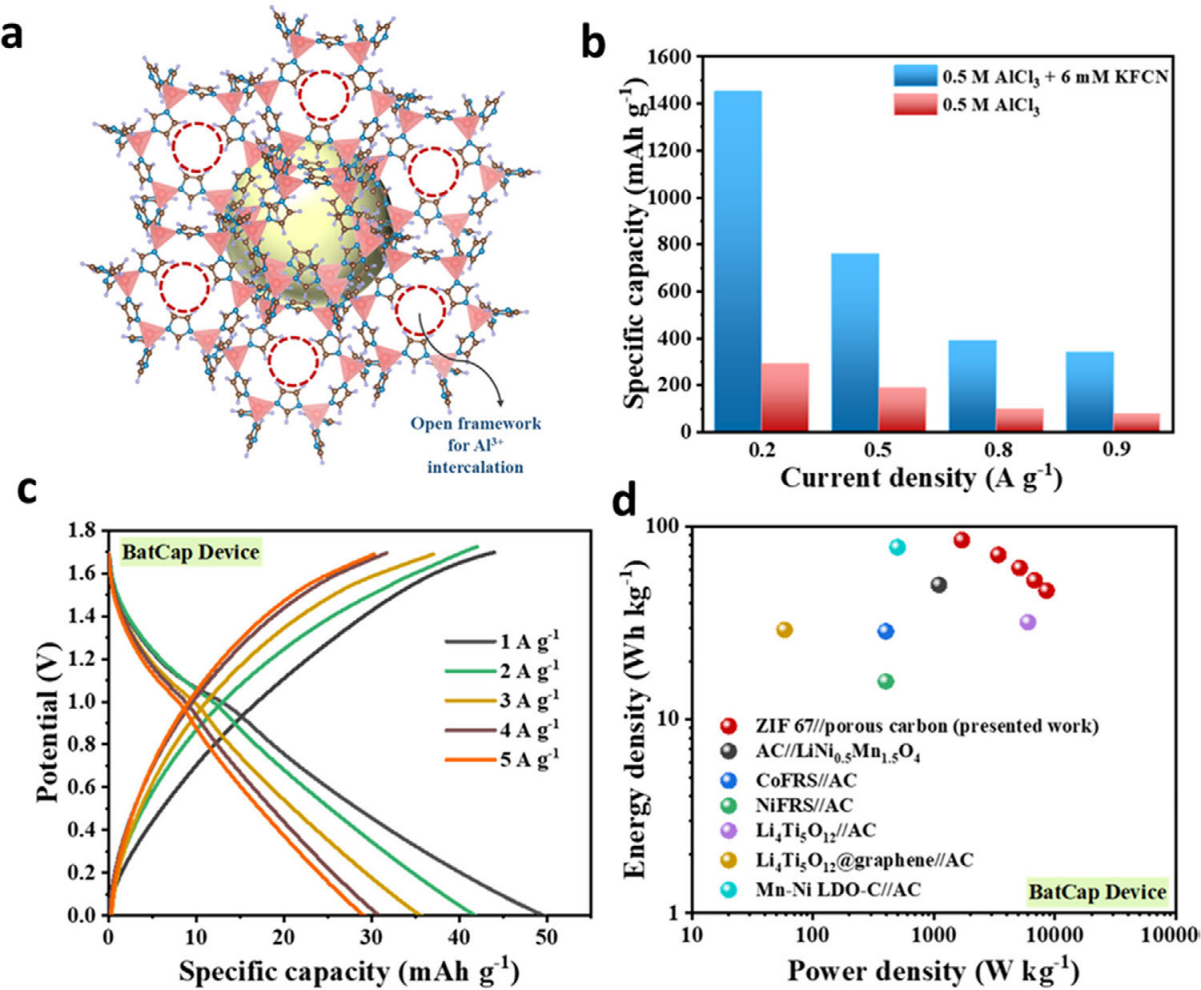
(a) Schematic illustration for the synthesis of ZIF‐67, (b) open crystal structure of ZIF‐67 for Al^3+^ ion intercalation and deintercalation, (c) comparative specific capacity of ZIF‐67 MOF with and without the addition of 6 mM KFCN + 0.5 M AlCl_3_ electrolyte, (d) Comparative CV curves at 1 mV s^−1^, (e) GCD curves of the fabricated ZIF‐67//porous carbon AICs, and (f) Ragone plot of the device compared with the previously reported hybrid capacitor devices. Reproduced with permission [99].

Metal‐organic frameworks (MOFs) have recently gained significant attention as promising electrode materials for Al‐HCs due to their tunable open frameworks, high porosity, structural stability, and abundant redox‐active sites. Recently, Chandra et al. [99] synthesized zeolite imidazole‐based open framework (ZIF‐67), a cobalt‐based imidazolate framework, via a methanol−mediated solvothermal process by reacting Co(NO_3_)_2_·6H_2_O with 2‐methylimidazole (organic linker) in the presence of polyvinylpyrrolidone, and used it as a cathode material for Al‐HCs (Figure 12a). In a three‐electrode system using 0.5 M AlCl_3_ as an electrolyte, ZIF‐67 delivered a discharge capacity of 288 mAh g^−1^ at 0.2 A g^−1^ (Figure 12b). Remarkably, the capacity was increased nearly fivefold (1452 mAh g^−1^ at 0.2 A g^−1^) when a redox‐modified electrolyte containing 6 mM potassium ferricyanide (KFCN) + 0.5 M AlCl_3_ was employed. The active‐material mass loading was maintained at ∼1 mg cm^−2^. However, this value should be interpreted as a combined electrode–electrolyte response rather than intrinsic Al^3+^ storage by ZIF‐67 alone. The CV curves in the redox‐modified electrolyte showed distinct cathodic and anodic peaks, indicating the possible intercalation of Al^3+^ ions compared with the unmodified electrolyte (0.5 M AlCl_3_). However, in the presence of ferricyanide these peaks cannot be assigned solely to Al^3+^ intercalation without additional mechanistic evidence. Instead, the overall charge‐storage process likely involves multiple contributions: (a) Al^3+^ intercalation/deintercalation within the ZIF‐67 electrode, (b) electrical double‐layer formation at the electrode/electrolyte interface, (c) surface‐controlled pseudocapacitive redox conversion between ferrocyanide and ferricyanide, and (d) charge storage in the bulk electrolyte through diffusion of ferricyanide species away from the interface after electron transfer. The presence of KFCN acted as a redox mediator in AlCl_3_ electrolyte, providing an additional reversible electrolyte redox couple that can accelerate charge‐transfer kinetics, rather than generating excess electrons. SEM‐EDS and ex situ XPS analyses further validated the successful intercalation of Al^3+^ ions into the ZIF‐67 crystal structure. A full‐cell Al‐HC device was constructed using ZIF‐67 as the positive electrode and ZIF‐67‐derived highly porous carbon as the negative electrode, operated within an extended potential window of 1.7 V using an electrode mass balance of m^+^ = 0.4m^−^. It delivered a specific capacity of 50 mAh g^−1^ at 1 A g^−1^ and achieved a high energy density of 86 Wh kg^−1^ at 2 kW kg^−1^ in redox‐modified electrolyte (Figure 12c,d). The device demonstrated a capacitance retention of 88% after 500 cycles at 2 A g^−1^. This study highlights the synergistic effects of the MOF architecture and redox‐additive‐modified Al‐based electrolyte in boosting Al‐HCs performance. Subsequently, the same research group reported a ZIF‐67/reduced graphene oxide hybrid (ZIF‐67_rGO), prepared via an in situ solvothermal growth of ZIF‐67 in the presence of rGO (where rGO was separately prepared by the reduction of graphene oxide using sodium borohydride), to overcome the poor intrinsic electrical conductivity of pristine ZIF‐67 MOF and explored its potential as an electrode material for Al‐HCs [100]. The introduction of rGO nanosheets within the ZIF‐67 framework formed an interconnected conductive network, which significantly enhanced electron transport and stability. As a result, ZIF‐67_rGO composite delivered a specific capacitance of 349 F g^−1^ at 1 A g^−1^, retaining 93% of its capacitance after 1000 cycles in the redox‐additive‐modified electrolyte (6 mM KFCN + 0.5 M AlCl_3_). However, despite this improved capacitive behavior, the assembled Al‐HC device (ZIF‐67_rGO as the positive and activated carbon as the negative electrode) operated within a narrower potential window of 1.2 V, resulting in a comparatively lower energy density of 18 Wh kg^−1^ at 0.530 kW kg^−1^, much smaller than that reported for the pristine ZIF‐67//porous carbon device (86 Wh kg^−1^ at 2 kW kg^−1^). The lower energy density can be attributed to the limited voltage window of the device configuration and the influence of rGO incorporation, which, while improving conductivity, dilutes the redox‐active content of the MOF and thus reduces the overall faradaic contribution.

Beyond the materials of PBAs and MOFs that were discussed above, 2D layered transition‐metal sulfides have attracted considerable attention as promising intercalation hosts for Al‐HCs due to their large interlayer spacing, abundant redox‐active sites, and improved structural stability, which enable efficient charge storage capability and long‐term cycling performance. For example, Chen et al. [101] hydrothermally synthesized Mo_15_S_19_ nanosheets by reacting ammonium molybdate tetrahydrate, thioacetamide, and urea on conductive carbon cloth, yielding nanosheets with an enlarged interlayer spacing of 0.80 nm. In a three‐electrode cell configuration using 1 M AlCl_3_ electrolyte, the Mo_15_S_19_ electrode achieved a specific capacitance of 400 F g^−1^ at 2 A g^−1^ with an active‐material mass loading of 0.6 mg cm^−2^, which was attributed to fast and reversible Al^3+^‐ion redox reactions facilitated by short diffusion pathways within the layered framework. Furthermore, ex situ Raman and XPS analyses verified the intercalation of Al^3+^ ions into the Mo_15_S_19_ structure, as evidenced by a decrease in the A*_1g_*/E^1^ *_2 g_* relative peak intensity ratio, and the reversible reduction of Mo^4+^ to Mo^3+^ during the charge/discharge process, respectively. A symmetrical Al‐ion supercapacitor (SC) device constructed from Mo_15_S_19_ electrodes delivered an energy density of 10.4 Wh kg^−1^ at a power density of 0.736 kW kg^−1^, while retaining 90.1% of its capacitance after 10 500 cycles, underscoring the material's excellent cycling stability. In contrast to the hydrothermally grown Mo_15_S_19_ nanosheets, Chatterjee and Das [102] prepared Cu‐doped SnS_2_ nanoflakes via a solid‐state method by grinding SnCl_2_·6H_2_O, CuSO_4_·5H_2_O, and thiourea, followed by annealing the mixture at 280°C under flowing N_2_. The SC performance of Cu‐doped SnS_2_ nanoflakes in a three‐electrode system at an active‐material mass loading of ∼1 mg cm^−2^ is significantly lower in 1 M AlCl_3_ aqueous electrolyte within a potential window of −0.2 V to 1.0 V. Although the 5% Cu doping improved the conductivity and facilitated Al^3+^ ion diffusion within the SnS_2_ framework, it resulted in only a 40% increase in specific capacitance (140 F g^−1^) compared with pristine SnS_2_ (98 F g^−1^) at a current density of 1 A g^−1^. The 5% Cu‐doped SnS_2_ electrode material exhibited a 20% decrease in the specific capacitance after only 500 cycles.

In addition to 2D transition‐metal sulfides, conducting polymers have also been explored as electrode materials for AICs. Wang et al. [103] employed the organic conducting polymer poly(3,4‐ethylenedioxythiophene):poly(4‐styrenesulfonate) (PEDOT:PSS) as an electrode material in a 1.2 M Al_2_(SO4)_3_ aqueous electrolyte for Al‐ion SCs. The PEDOT:PSS electrode exhibited distinct redox peaks at slow scan rates (< 2 mV s^−1^), corresponding to faradaic charge‐transfer reactions between PEDOT:PSS and Al^3+^. It delivered a specific capacitance of 269 F g^−1^ (78 mAh g^−1^) at 0.2 A g^−1^. The active‐material loading was maintained at ∼1 mg cm^−2^ for three‐electrode measurements. Kinetic analysis indicated slow Al‐ion intercalation in the PEDOT:PSS framework, suggesting a bulk diffusion‐controlled charge‐storage mechanism. At higher scan rates (> 2 mV s^−1^), Al^3+^‐species diffusion became less significant, and charge storage became predominantly surface‐controlled with a more capacitive character. The ex situ XPS analysis of the discharged electrode revealed the presence of a strong Al 2p peak, confirming the intercalation of Al ions within the active channels of PEDOT:PSS conducting polymer, although XPS alone does not unambiguously prove bulk intercalation. Furthermore, an asymmetric AIC device was assembled using PEDOT:PSS as the cathode and activated carbon (AC) as the anode on carbon cloth; this device achieved a specific capacitance of 117 F g^−1^ at 0.3 A g^−1^, corresponding to an energy density of 41.6 Wh kg^−1^ at a power density of 0.24 kW kg^−1^ within a potential window of 1.6 V. The device also demonstrated excellent cycling stability, retaining 90% of its initial capacitance after 3000 cycles at 0.4 A g^−1^.

The charge storage behavior of the conductive polymer polyaniline emeraldine salt (PANI‐ES) has also been studied in aqueous Al(NO_3_)_3_ electrolyte [104]. The typical redox transitions of leucoemeraldine ↔ emeraldine and emeraldine ↔ pernigraniline redox processes were observed, indicating reversible redox activity. The charge storage mechanism was primarily governed by anion doping/dedoping along with partial participation of Al^3+^ ions. The interaction of Al^3+^ ions with PANI induced swelling/shrinking of its grain size structure. As a result, PANI‐ES delivered a specific capacitance of 317 F g^−1^ at 1 A g^−1^ in a three‐electrode open cell configuration at a mass loading of ∼2.5 mg cm^−2^. The symmetrical full cell device achieved a specific capacitance of 269 F g^−1^ at 10 A g^−1^ within a voltage window of 0.8 V, with capacitance retention of 88% after 1000 cycles.

Overall, beyond carbon‐based materials and transition metal oxide/hydroxide electrodes, PBAs, MOFs, layered transition‐metal sulfides, and conducting polymers have also emerged as promising electrode materials for AICs. PBAs and MOF‐based systems are particularly attractive because their well‐defined porous architectures and tunable chemistry enable efficient ion transport and enhanced charge‐transport kinetics, while conductive hybridization and addition of redox‐active electrolytes further improves their electrochemical performance in terms of specific capacitance and energy density. Their practical use is limited by constraints related to conductivity, structural stability, and the accessibility of ions to the electrolyte. This reinforces the need to match the architecture of the host with the dominant Al‐bearing species. Pure EDLC‐type AICs remain relatively scarce, as many carbon‐based electrodes, including nitrogen‐doped porous carbons and MWCNTs, exhibit mixed electrical double‐layer and pseudocapacitive behavior through heteroatom doping, surface functional groups, or limited ${\mathrm{AlCl}}_{4}^{-}\;$ intercalation. These systems generally provide excellent rate capability but are susceptible to interfacial degradation, including carbon dissolution and pore blockage. In contrast, pseudocapacitive electrodes based on h‐MoO_3_, PPy@MoO_3_, α‐MoO_3_@PPy, and V_2_O_5_/carbon composites achieve higher charge‐storage capability through reversible surface or near‐surface faradaic reactions, although long‐term performance may be limited by structural degradation and dissolution of active species. HCs, such as P‐TiO_2_‐MRs//AC, CuFe‐PBA//AC, and ZIF‐67//porous carbon, further enhance energy density by combining battery‐type and capacitive electrodes, but their performance is more strongly influenced by electrolyte chemistry, electrode balancing, and interfacial stability. Therefore, meaningful comparisons among AICs should also consider practical factors such as cell configuration, active‐material loading, and the basis used for calculating energy and power densities.

## 4 Electrolytes: The Core of Al^3+^ Storage in Supercapacitors and Batteries

**FIGURE 13:**
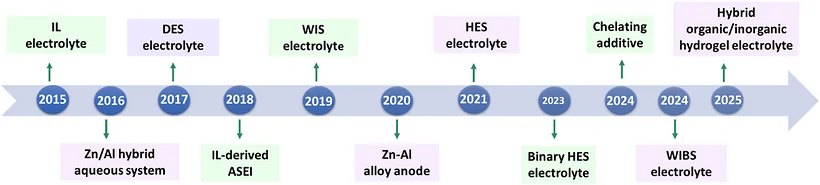
Highlights of electrolyte and electrode interfaces development for Al‐ion‐based systems: Al‐ion batteries with ionic‐liquid electrolytes (2015) [85]; Zn/Al hybrid systems with aqueous electrolytes (2016) [111]; DES‐based Al‐ion electrolytes (2017) [112]; ionic liquid‐derived ASEI formation (2018) [61]; water‐in‐salt Al‐ion electrolytes (2019) [113]; Zn‐Al alloy anodes (2020) [114]; hydrated eutectic electrolyte (2021) [115]; binary hydrated eutectic electrolytes (2023) [116]; chelating electrolyte additives (2024) [117]; water‐in‐bisalt electrolytes (2024) [118]; hybrid organic/inorganic hydrogel electrolytes (2025) [119].

The electrolyte determines which Al‐bearing species carry charge, how they are solvated and desolvated at interfaces, and which interphases self‐assemble or can be engineered on electrodes. Because Al^3+^ is small and strongly Lewis‐acidic, the electrolyte design ultimately determines transport, kinetics, and the thermodynamic feasibility of aqueous and non‐aqueous systems [105, 106]. Thus, electrolytes have been extensively studied over the years to enhance the performance and stability of Al‐ion systems (Figure 13). In water, the narrow electrochemical stability window, together with the triad of hydrogen evolution, acid‐induced corrosion, and Al_2_O_3_/Al(OH)_3_ passivation, hinders reversible aluminum plating and stripping and reduces practical voltages for the development of MIHCs that involve Al deposition and stripping [107]. Recent progress targets the reduction of “free water” content and the reshaping of solvation via WISE electrolyte formulations, hydrated eutectic electrolytes, careful co‐solvent and additive selection, and intentional interphase regulation [108, 109]. These strategies widen the electrolyte's electrochemical window, reduce desolvation and charge‐transfer penalties, and stabilize the aluminum interface. Beyond water, Lewis‐acidic chloroaluminate ionic liquids serve as the kinetic benchmark. However, these ionic liquids are moisture‐sensitive, corrosive, and costly, motivating the exploration of chloride‐lean ionic liquids, DES, and interface‐priming approaches. Finally, quasi‐solid gels and ionogels adapt these chemistries into leak‐free, flexible formats, suppressing water‐induced side reactions and extending low‐temperature operation, thereby enabling wearable Al‐ion supercapacitors [110]. The following subsections are organized accordingly: aqueous and non‐aqueous (ionic liquid/DES) electrolytes, in relation to the device classes for which they are most suitable, such as supercapacitors, batteries or hybrid devices, followed by gel and solid‐state media for flexible devices. Across all chemistries, the unifying design rule is to link solvation structure to the desolvation barrier and interfacial kinetics under realistic current densities. To make this discussion more device‐relevant, the electrolyte families were evaluated below in terms of ionic conductivity and electrochemical stability window, as well as chemical/electrochemical stability, corrosion/passivation of Al and non‐Al current collectors, compatibility with binders and separators, moisture tolerance during processing, and safety at cell and end‐of‐life levels. This framework is of particular significance for AlCl_3_‐based chloroaluminate liquids, where exceptional laboratory reversibility coexists with pronounced Lewis acidity, chloride activity, and elevated corrosion risk.

**FIGURE 14:**
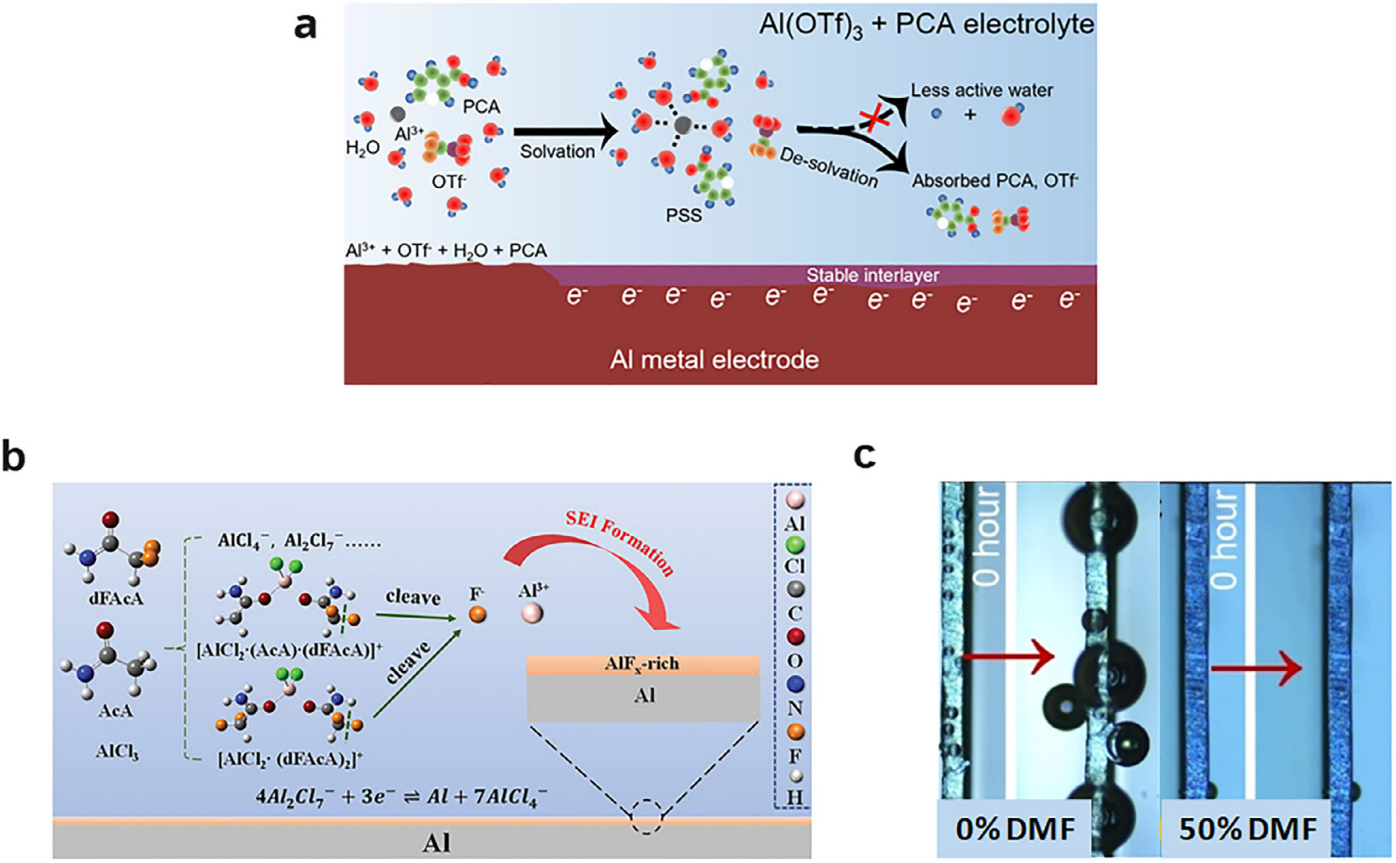
Schematic illustration of the working principles of representative electrolyte families in Al‐ion‐based energy‐storage systems: (a) low‐concentration aqueous electrolytes, (b) DES, and (c) suppressing hydrogen evolution in organic/mixed electrolytes in a five‐hour test. Panels (a–c) are reproduced with permission from Refs. [117, 132, 142], respectively.

A summary of representative examples of the electrolyte families discussed in this review, highlighting their typical compositions and their relevance to different Al‐based energy‐storage architectures is presented in Figure 14.

### 4.1 Aqueous Electrolytes

#### 4.1.1 Hydrolysis Issues, Narrow Potential Window

The 1.23 V ESW of water, defined by the hydrogen‐evolution reaction (HER) and oxygen‐evolution reaction (OER) equilibria, intrinsically limits aqueous electrolytes for Al‐ion storage, restricting the attainable cell voltage and inducing parasitic gas evolution [120]. While the practical voltage window is often broadened by interfacial passivation effects, the standard potential of the Al^3+^/Al couple (E°= −1.66 V vs SHE) lies far below the HER potential. Therefore, aluminum deposition inevitably competes with the HER, while OER may occur at the counter electrode [121]. Consequently, electrochemical reduction of Al^3+^ in water is thermodynamically unfavorable, and the anode is dominated by HER and corrosion rather than reversible metal plating. Beyond electrochemical instability, the strong Lewis acidity of hydrated Al^3+^ drives extensive hydrolysis of the [Al(H_2_O)_6_]^3+^ complex, generating protons and acidic by‐products that further corrode both electrode and current‐collector surfaces. The resulting Al(OH)_3_/Al_2_O_3_ species form a dense, electronically insulating film that blocks ionic transport and gives rise to large interfacial impedance [106, 121]. This spontaneous passivation is chemically stable yet electrochemically inert and prevents uniform Al stripping and plating and causes rapid capacity fading due to an increase in equivalent series resistance (ESR. For practical aqueous AIC cells, corrosion testing should extend beyond the Al metal electrode to include inactive components, such as stainless steel, Ni, Ti, Al foil, graphite/carbon, or Mo current collectors [122]. Parasitic corrosion currents can otherwise be misinterpreted as electrode redox, accelerating self‐discharge and capacity fading. Particular caution is required when using Cl‐containing aqueous electrolytes because Cl^−^ can cause pitting and current collector degradation. However, Cl‐free sulfate, nitrate, triflate, WISE/water‐in‐bisalt (WIBS), and hydrated eutectic solvent (HES) formulations can mitigate this risk but may introduce trade‐offs in cost, conductivity, Al speciation, or reversibility. Mechanistic studies have revealed that the interplay among HER, acid‐driven corrosion, and oxide passivation dictates the electrochemical behavior of aluminum in aqueous systems, leading to low CE and poor cycling reversibility [123, 124]. Therefore, progress in aqueous Al‐ion based energy storage depends on strategies that (i) tailor the solvation environment to suppress hydrolysis, (ii) expand the apparent ESW via high‐concentration or hybrid electrolytes, and (iii) stabilize the metal/electrolyte interface through surface coatings or artificial interphases. Aqueous electrolytes are generally well suited to capacitive and pseudocapacitive Al‐based systems because of their high ionic conductivity, low cost, and improved safety, which favor fast ion adsorption and surface or near‐surface redox processes. However, their narrow electrochemical stability window limits their applicability in hybrid Al‐ion systems.

#### 4.1.2 Strategies to Stabilize Al^3+^ in Water

The following strategies stabilize aqueous Al systems by reducing water activity, replacing water in the Al^3+^ coordination environment, or forming protective interphases. Water‐in‐salt electrolytes and related concentrated electrolytes based on Al(OTf)_3_ have salt content that approaches the dissolution limit, forcing anions to replace water molecules in the primary solvation shell of Al^3+^, widening the apparent ESW from ∼1.23 V to 2.2–2.65 V. Reversible Al stripping and plating was achieved in 5 M Al(OTf)_3_, accompanied by the stabilization of MnO_2_ cathode, which exhibited a voltage plateau between 0.8 and 1.3 V, a capacity of ∼425 mAh g^−1^, and a CE of ∼80% [125]. A PBA cathode in a three‐electrode system showed a plateau in the 0.5–1.0 V range vs Ag|AgCl reference electrode and capacity ∼116 mAh g^−1^. The CE exceeded 105%, indicating the presence of irreversible processes [28]. Lower concentrations of the triflate salt, however, fail to stabilize the Al anode interface [126]. A much broader ESW of 4.0 V was achieved in 15 M Al(ClO_4_)_3_, enabling ClO_4_^−^ anion intercalation into graphite at 1.2–1.6 V vs Ag|AgCl RE, ∼35 mAh g^−1^ capacity and 95% CE [127].

To further broaden the ESW of Al(OTf)_3_ electrolytes, the WIBS concept was introduced. This approach further reduces water activity by surpassing the solubility limit of Al(OTf)_3_ and incorporating concentrated LiTFSI or other salts containing bulky, weakly coordinating anions [118] The combination of 1 M Al(OTf)_3_ with 15 M LiOTf yielded an ESW of 4.35 V. The corresponding Al | Al_x_MnO_2_ full cell delivered a capacity of 364 mAh g^−1^ with a CE of ∼95% [78]. The ESW of 5 M Al(OTf)_3_ was increased from 2.65 V to 3.8 V by altering the solvation structure of Al^3+^ with 0.5 M LiOTf, where Li ions promote a hydrophobic environment that stabilizes the Al interface and suppresses HER, however, the capacity of Al|MnO cell was not stable [128].

Protic additives such as H_3_PO_4_ moderate the Lewis acidity of Al^3+^ and tailor interfacial chemistry. The addition of 1 M H_3_PO4 to 5 M Al(OTf)_3_ yields a thinner, AlPO_4_‐containing SEI that suppresses HER and improves durability [129]. The Al|PANI device showed 225 mAh g^−1^ at 0.5 A g^−1^ upon activation and good cycling stability with CE ∼98%. In another study, perchloric acid was employed as a pH regulator, maintaining the electrolyte at pH < 3.0 to achieve multiple beneficial effects: they prevent Al(OH)_3_ precipitation by stabilizing Al^3+^ ions in solution, enhance electrode reaction kinetics through proton participation in redox processes, and promote interfacial layer formation on the Al surface, thereby improving plating/stripping reversibility and suppressing corrosion [130, 131]. Highly concentrated aqueous electrolytes are particularly important for pseudocapacitors and hybrid Al based devices because they can increase the apparent stability window and moderate interfacial reactivity. Many examples are taken from battery studies and are cited here primarily because these properties are also desirable for hybrid architectures.

Partial substitution of water with organic substances lowers Al^3+^ desolvation energy and enhances ionic mobility. A hybrid electrolyte (1 M Al(OTf)_3_ in N,N‐dimethylformamide (DMF)/dimethyl methylphosphonate (DMMP)/H_2_O = 6:1:4, v:v:v) strengthens water O–H bonds via electron donation from polar DMF and DMMP [132]. It shifts the H_2_ evolution onset by 0.56 V (from −1.35 V to −1.93 V), enhancing anode stability and enabling Al|Al_x_MnO_2_ cells to deliver 400 mAh g^−1^ with ∼99% CE. Although DMF toxicity limits practical use, the concept guides the design of safer analogues with similar solvation control. Adding 0.6 M pyridine‐3‐carboxylic acid to 1 M Al(OTf)_3_ yielded comparable performance to the former system [117]. Furthermore, a chelation‐driven electrolyte design was proposed using bis(2‐methoxyethyl)amine to achieve broad ESW of 2.2 V and stable Al interface, however, CE was ∼94% with an organic cathode material and ∼90% with an inorganic [133].

Hydrated eutectic solvents (HESs) are coordination‐driven, hydrogen‐bonded liquids formed from hydrated metal salts and neutral ligands, in which bound water plays a key structural role. They differ from classical DES (discussed below) by incorporating controlled hydration, yielding a hybrid solvation environment that combines the stability and safety of aqueous systems with the wide electrochemical window of nonaqueous DESs In Al‐ion HESs, the solvation environment of Al^3+^ is modified by coordination with both water and organic ligands (e.g., N,N‐dimethylacetamide, urea, ethylene glycol, methyl carbamate). These ligands interact via hydrogen bonding and ion–dipole coordination, forming hybrid complexes such as [Al(L)_x_(H_2_O)_y_]^3+^, where L represents the ligand. The result is a water‐deficient, weakly solvated, yet highly conductive medium that stabilizes the Al/electrolyte interface and supports reversible metal deposition. The fundamental viability of this approach, including its material‐oriented adaptations, was explored in several early studies [115, 134, 135]. Within a short period, several applied, materials‐oriented studies emerged. The Al(OTf)_3_‐glycerol‐glycerophosphate HES electrolyte for aluminum‐ion aluminum‐ion batteries simultaneously reduces water activity, constructs a protective SEI, and enables wide‐temperature, corrosion‐free, HER‐suppressed Al cycling. It allowed smooth Al deposition in wide temperature range from −20 to 60°C, showed over 1000 h of symmetric‐cell stability and good cycling stability of Prussian white|Al cell [136]. Similar results were achieved using Al(OTf)_3_‐acetonitrile‐triethyl phosphate HES [137] and Al(ClO_4_)_3_ hydrate with methyl carbamate [138].

Furthermore, coupling of such electrolyte systems with other cations established multimetal hybrids. The ternary HES electrolyte was engineered to enable fully reversible deposition and stripping of Al_x_MnO_2_ on a conductive carbon collector, thereby realizing a cathode‐free Al battery architecture alongside reversible Al‐Mn plating and stripping at the anode [139]. For that, Al and Mn hydrous nitrates were mixed with N,N‐dimethylacetamide in the 1:1:20 molar ratio, producing a mixed‐cation solvation environment with reduced water activity. Another approach is focused on Al‐Zn hybrid systems, where Al_2_(SO_4_)_3_‐based HES was combined with a Zn anode [134] or an Al‐Zn alloy anode [135], offering a substantially lower‐cost and more environmentally benign alternative to triflate‐ or perchlorate‐based electrolytes.

Finally, electrolyte‐derived artificial solid‐electrolyte interphases offer a promising route to enhance the stability of Al‐ion anodes, as surface passivation remains a major kinetic limitation. Pre‐formed, ion‐conductive interphases can effectively regulate interfacial reactions and enable reversible Al plating and stripping even in aqueous environments [123].

The first artificial SEI (ASEI) on Al metal was reported for the AlCl_3_‐[EMIm]Cl ionic liquid, where direct contact with the melt simultaneously dissolved the native Al_2_O_3_ layer and generated a new, protective interphase that suppressed further oxide formation [61]. This ASEI remained even after transferring the electrode into aqueous Al(OTf)_3_, demonstrating its chemical robustness across distinct electrolyte environments. In contrast, AlCl_3_‐urea ionic liquids exhibited the opposite behavior: the presence of free chloride ions intensified corrosion of Al surface [140].

Combining high‐concentration or hybrid aqueous electrolytes with additive complexation, co‐solvent structuring, and interphase control has transformed water from a limitation into a design element. Aqueous Al systems now reach ESWs up to ∼2.6 V and 4.35 V in concentrated Al(OTf)_3_, and in selected bisalt or perchlorate electrolytes, respectively, rate capabilities over 10 A g^−1^, and cycling stability extending to hundreds or thousands of hours [108]. These advances position aqueous aluminum electrolytes as practical, safe, and sustainable platforms for multivalent‐ion energy storage. Hydrated eutectic and chelating designs have enabled CEs of 95%–99% and stable cycling over hundreds to thousands of cycles. Through solvation engineering via hydrogen‐bond modulation, Lewis‐base coordination, and controlled hydration, aqueous aluminum systems now approach the reversibility and voltage tolerance required for safe, low‐cost, high‐performance multivalent batteries. However, a high CE value or stable cycling alone should not be considered proof of corrosion‐free operation [141]. Long‐duration open‐circuit storage, symmetric cell tests, post‐mortem inductively coupled plasma analysis/XPS/SEM analysis, and direct corrosion measurements are necessary to distinguish reversible Al plating/stripping from HER, passivation, dissolution, or parasitic charge compensation. At the same time, the widespread use of highly concentrated salts such as LiTFSI or Al(OTf)_3_ presents a serious obstacle to the sustainability of aqueous aluminum‐ion systems. These salts are both costly and environmentally burdensome: Al(OTf)_3_ is more expensive than many common ionic liquids, undermining the economic viability of high‐concentration formulations, while perchlorate‐based electrolytes introduce substantial toxicity concerns, limiting their applicability in safe and scalable technologies [134]. Consequently, future research should prioritize the development of environmentally benign, low‐cost aqueous electrolytes that maintain wide electrochemical stability windows and support reversible Al deposition/stripping without relying on fluorinated or perchlorate‐based chemistries. This shift is critical for advancing aluminum‐based energy storage toward genuinely sustainable and economically competitive realizations. Representative examples of the electrolyte families discussed in this review, including their typical compositions and relevance to different Al‐based energy‐storage architectures, are summarized in Figures 4a, 10a, and 14.

### 4.2 Non‐Aqueous Electrolytes

#### 4.2.1 Ionic Liquids

Ionic liquids (ILs) are salts composed of bulky organic cations and inorganic or organic anions that remain liquid at ambient conditions. Relative to molecular solvents, they offer a distinctive combination of negligible vapor pressure, exceptional thermal and chemical stability, high ionic conductivity, wide electrochemical stability windows, and strong solvation power [143, 144]. Lewis‐acidic chloroaluminate ionic liquids remain the benchmark media for reversible aluminum deposition and graphite anion intercalation. They are typically prepared by mixing AlCl_3_ with an organic chloride ([R]Cl) such as [EMIm]Cl or 1‐butyl‐3‐methylimidazolium chloride ([BMIm]Cl). Strong Lewis acid‐base interactions between AlCl_3_ and chloride anions generate a room‐temperature chloroaluminate melt whose speciation and acidity are dictated by the AlCl_3_:[R]Cl molar ratio. Adjusting this ratio tunes the relative populations of Cl^−^, $AlCl_{4}^{-}$, $Al_{2}Cl_{7}^{-}$, and the organic cation [R]^+^, thereby controlling both ionic structure and Lewis acidity. Increasing the fraction of $Al_{2}Cl_{7}^{-}$ can increase plating capacity and rate capability, but also increases Lewis acidity, chloride activity, moisture sensitivity, viscosity, and corrosion risk. Under acidic conditions (AlCl_3_:[R]Cl > 1), $Al_{2}Cl_{7}^{-}$ becomes responsible for Al plating, while $AlCl_{4}^{-}\;$ intercalates into the cathode, enabling cell operation. The most frequently used composition is ∼1.3:1 and has conductivities of 6–8 mS cm^−1^ at 25°C. The following reactions occur at the anode and cathode:

$$\mathrm{Anode}\;:\to 4Al_{2}Cl_{7}^{-}+3e^{-}⇌7AlCl_{4}^{-}+Al$$

$$\mathrm{Cathode}\;:\to xC+AlCl_{4}^{-}⇌Cx\left(AlCl_{4}^{-}\right)+e^{-}$$

where C is graphite or another cathode active material. Since Al^3+^ does not shuttle as a free cation between the electrodes in these chloroaluminate ionic liquids, the active ionic species are chloroaluminate complexes generated within the electrolyte, while the solid electrodes (Al metal and graphite) serve as hosts for plating/stripping and anion intercalation, respectively. This makes the capacity of the device directly proportional to the concentration of $Al_{2}Cl_{7}^{-}$. Charging terminates once $Al_{2}Cl_{7}^{-}$ is fully consumed during Al plating at the anode. The $Al_{2}Cl_{7}^{-}$ fraction can be increased through ionic liquid design: its concentration sharply increases as the AlCl_3_:[R]Cl ratio rises from about 1.1 to 1.8, which is the practical window for Al‐plating melts. The upper limit of this ratio is bounded by AlCl_3_ solubility in the melt, whereas a ratio of ∼1.3 is widely adopted because it provides the best compromise between Lewis acidity, ionic conductivity, viscosity, and suppression of parasitic reactions [145]. Therefore, the AlCl_3_:[R]Cl ratio should be considered not only a kinetic variable but also a safety and compatibility variable, and the AlCl_3_:[R]Cl ratio, water content, electrolyte excess, separator/current‐collector materials, binder chemistry, and storage/assembly atmosphere should be reported. Tuning the cation from [EMIm]^+^ to [BMIm]^+^ suppresses crystallization in chloroaluminate ILs, allowing the AlCl_3_‐[BMIm]Cl (N = 1.5) electrolyte to operate down to –100°C. This system had conductivity of 3.28 mS cm^−1^ at 0°C and 0.28 mS cm^−1^ at –35.5°C, and reversibly operated at down to ‐30°C with near 100 % CE [146].

Dilution with organic solvents can significantly improve the performance of ionic‐liquid electrolytes [147]. In the case of AlCl_3_/[EMICl, a 1:5 mixture with fluorobenzene weakens the strong cation–anion electrostatic interactions while retaining the active $Al_{2}Cl_{7}^{-}$/ $AlCl_{4}^{-}$ speciation, thereby markedly enhancing ion mobility and reducing viscosity. This modification doubles the limiting current density and yields uniform Al deposition with exceptionally high plating/stripping CE up to 99.9%, while also enabling stable operation with low‐cost polypropylene membranes [148].

Despite excellent reversibility, chloroaluminate ILs suffer from strong hygroscopicity and corrosivity: trace H_2_O induces hydrolysis of $Al_{2}Cl_{7}^{-}$ and evolution of HCl, degrading electrodes and current collectors. Their oxidative stability (∼2.4–2.5 V vs Al^3+^/Al) also restricts the range of compatible high‐voltage cathodes, favoring relatively stable hosts such as graphitic carbons and selected sulfur‐based systems. Chloride‐free ionic liquids were therefore developed to reduce these corrosive pathways, offering greater electrochemical stability, broader electrode compatibility, and safer operation, and thus marking an important advance toward practical aluminum‐based energy‐storage technologies. Nevertheless, they frequently trade these gains for lower conductivity and more complex interphase chemistry. These systems are based on Al(OTf)_3_ or Al(ClO_4_)_3_ with [BMIm]TFSI, [BMPyr]TFSI, or similar cations suppress corrosion and moisture sensitivity. For example, Al(OTf)_3_/[BMIm]TFSI exhibits an ESW of 3.5–3.8 V, viscosity ∼30 mPa s, and supports stable plating/stripping over 1,000 h after forming an ionic liquid‐derived interphase [149].

At the full‐cell level, AlCl_3_‐based chloroaluminate ILs should be considered enabling but not intrinsically benign electrolytes. Trace moisture can alter chloroaluminate equilibria and generate HCl‐containing media that can corrode current collectors, tabs, seals, and electrode interfaces. Current collectors that are stable in conventional supercapacitors, including stainless steel, Ni, Ti, and Al, may not be inert in acidic AlCl_3_/[EMIm]Cl. Therefore, Mo, W, glassy carbon, carbon‐coated, or otherwise protected collectors are often more suitable, but their long‐term stability must still be verified. Binder and separator compatibility is also non‐trivial since Lewis‐basic or fluorinated polymers can alter chloroaluminate speciation or undergo side reactions. Thus, scaling up chloroaluminate‐based AICs requires dry processing, corrosion‐resistant cell hardware, validated binder/separator packages, controlled electrolyte excess, and end‐of‐life neutralization protocols. The broader device‐level consequences of electrolyte corrosion, current‐collector passivation, binder compatibility, electrolyte toxicity, and manufacturability are further discussed as commercialization bottlenecks in Section 7.

Composite electrolytes based on ionic liquids are particularly promising for hybrid Al‐ion based devices, as they offer a combination of broader electrochemical stability, an improved form factor and greater interfacial control. They remain the state‐of‐the‐art battery platform and less suitable for purely non‐redox supercapacitors, which require maximized ion mobility. Challenges of cost, moisture sensitivity, and corrosiveness continue to motivate the transition toward chloride‐free or deep‐eutectic derivatives with improved safety and processing tolerance.

#### 4.2.2 Deep Eutectic Solvents

DES are mixtures of two or more solid components that, at specific ratios, form a room‐temperature liquid through pronounced hydrogen‐bonding, Lewis acid‐base or other interactions. The most widely used aluminum precursors for DESs are AlCl_3_, Al(OTf)_3_, and Al(ClO_4_)_3_, which provide access to chloroaluminate or oxyanion‐coordinated aluminum complexes depending on the chosen formulation. These salts are typically combined with organic hydrogen‐bond donors such as urea [112], acetamide [142], or guanidine [150], whose strong Lewis basicity and extensive hydrogen‐bonding capabilities enable the formation of low‐melting eutectic liquids. DESs’ lower melting points arise from the strong, highly disordered intermolecular network that stabilizes the liquid phase and yields low vapor pressure. DESs often show lower volatility, lower cost, and simpler preparation than imidazolium chloroaluminate ILs, although toxicity, flammability, moisture sensitivity, and corrosivity remain component‐dependent. Owing to the wide range of available hydrogen‐bond donors and acceptors, their properties can be tuned through rational choice of components and present a potential replacement of ILs and Li‐salt mixtures [109]. Anhydrous DESs contain no free water and rely entirely on interactions between the constituent salt or hydrogen bond acceptor and organic hydrogen bond donor. This yields an ionic liquid‐like environment that is simpler and less expensive to prepare. In the prototypical AlCl_3_:urea (1.3:1) system, the transition from $AlCl_{4}^{-}$ to $Al_{2}Cl_{7}^{-}$ species results in an ESW reaching 3.0–3.2 V and enables reversible Al plating and stripping at room temperature [112]. Importantly, AlCl_3_‐based DESs reduce some cost barriers, but they do not automatically eliminate chloride‐driven corrosion. Free chloride content, Lewis acidity, water uptake, and compatibility with metal current collectors should be considered. Modifying Lewis bases is an effective way of tuning coordination structures, ion speciation and interfacial chemistry. This enables substantial improvements to be made on the properties of the resulting electrolytes. Substituting urea with N‐methyl‐ or N‐ethyl‐urea in AlCl_3_‐based ionic‐liquid analogs sharply reduces viscosity and enhances conductivity by shifting speciation toward neutral Al‐urea adducts [151]. Fluorination of acetamide substantially enhances the high‐voltage stability of AlCl_3_/acetamide DESs, lifting their oxidation onset from 2.21 to 2.78 V by modulating coordination chemistry and suppressing over‐oxidation [142]. However, the moderate conductivity (1–3 mS cm^−1^) and high viscosity (100–150 mPa s) remain performance‐limiting challenges, often requiring operation at 40–60°C. Nevertheless, DESs outperform conventional ILs with respect to cost, scalability, and air tolerance. Organic electrolytes are more appropriate for hybrid devices and batteries than for supercapacitors, as their wider voltage window can benefit intercalation‐based charge storage, although cost, flammability, and slower ion transport may reduce their attractiveness for purely capacitive Al‐based systems.

#### 4.2.3 Tunability, Stability, and Conductivity

The performance of ionic liquid and DES electrolytes depends on three tightly coupled factors: (i) speciation control via the aluminum salt to donor ratio, which determines the delicate balance of charge‐carrying species; (ii) transport properties governed by viscosity, ion size, and water content, which control rate capability and ESW; (iii) corrosivity and moisture tolerance, which define practical cell stability and manufacturability [152]. Chloride‐lean ILs and DESs offer improved oxidative stability (≤3.8 V) and minimal corrosion, but have lower conductivity. Nonetheless, classical chloroaluminates still deliver the best kinetics.

### 4.3 Gel and Solid‐State Electrolytes

Gel and solid‐state electrolytes combine the safety of aqueous systems with the mechanical robustness required for flexible or wearable devices. Gel polymer electrolytes (GPEs) and hydrogels, based on matrices such as poly(vinyl alcohol) (PVA), poly(vinylidene fluoride‐co‐hexafluoropropylene) (PVDF‐HFP), poly(ethylene oxide) PEO, or polyurethane, can host aqueous WISE, IL, or DES phases [153]. These materials exhibit room‐temperature ionic conductivities of 10^−4^–10^−2^ S cm^−1^, sufficient for thin‐film and high‐surface‐area supercapacitors. Solid‐state Al‐ion devices remain limited by slow transport of coordinated chloroaluminate species, unstable Al‐electrolyte interfaces, and imperfect solid–solid contact during plating/stripping [154]. These issues are intensified by the narrow speciation balance of $AlCl_{4}^{-}$/$Al_{2}Cl_{7}^{-}$, which readily shifts under confinement, raising overpotentials and accelerating degradation. Although advanced gel and solid‐state electrolytes (SSE) offer progress, achieving fast ion conduction together with interfacial stability, mechanical robustness, and scalable fabrication remains a central unresolved challenge. Polymer matrices, binders, and separators may coordinate AlCl_3_ or consume electroactive chloroaluminate species, therefore, these components should be treated as part of electrolyte design rather than inert cell accessories. To address these issues, binder‐free electrodes, carbon cloth/current collectors, chemically inert binders, polyacrylonitrile‐type separators, and protective coatings should be screened by soak tests and Raman/FTIR/XPS/ICP analysis before electrochemical cycling.

A hypercoordinated AlCl_3_‐rich SSE enabled chain‐assisted chloroaluminate transport, achieving 0.89 mS cm^−1^ ionic conductivity and a ∼2.6 V ESW [155]. Ionic conductivity was further improved to ∼7 mS cm^−1^ through AlF_3_ modification, which promotes $Al_{2}Cl_{7}^{-}$→ $AlCl_{4}^{-}$ conversion and enhances anion transport [156]. A solid polymer electrolyte based on [EMIm]Cl‐AlCl_3_, PEO, and fumed SiO_2_ doubled the ionic conductivity to ∼13 mS cm^−1^ and extended ESW to 2.8 V [157]. Gel electrolytes provide an order‐of‐magnitude enhancement in ionic conductivity to ∼10^−2^ S cm^−1^, enabling ultrafast solid‐state operation of Al‐ion devices. Soft, ionic liquid‐rich GPEs induce high surface area fractal Al deposition morphologies that lower interfacial overpotentials and support charging rates up to 1000 A g^−1^. Full Al/GPE/3D‐graphene cells deliver 122 mAh g^−1^ and maintain stable cycling over 20 000 cycles [158].

Solid, gel, and polymer electrolytes each offer distinct advantages for advancing Al‐ion batteries, and no single platform has yet emerged as a definitive“winner” [119, 159]. They are most suitable for hybrid and pseudocapacitive Al‐based devices, particularly flexible or solid‐state configurations, because they improve safety, mechanical integrity, and interfacial stability, although their lower ionic mobility generally makes them less favorable for purely non‐redox supercapacitors. Together, their complementary development indicates that progress is occurring across all electrolyte classes rather than converging on a single dominant solution.

In summary, electrolytes remain the bottleneck for practical aluminum‐based energy storage. Progress across aqueous, non‐aqueous, gel, polymer and solid‐state systems increasingly depends on controlling Al‐complex speciation, stabilizing interfaces and balancing ionic conductivity with electrochemical stability (Table 2). Aqueous media reached ESWs far beyond the 1.23 V limit through WISE/WIBS, HESs and chelating additives, suppressing HER and enabling reversible Al deposition with CEs of 95%–99%. Yet their dependence on costly salts and limited anodic compatibility underscores the need for low‐cost, non‐fluorinated alternatives. Chloroaluminate ILs and DESs still deliver the fastest kinetics and most mature mechanisms for reversible Al plating and graphite intercalation, while chloride‐lean formulations enhance stability (>3.5 V) and reduce corrosiveness, often with reduced conductivity. Parallel advances in solid, gel and polymer electrolytes now yield conductivities comparable to liquids: hypercoordinated frameworks (∼1 mS cm^−1^), AlF_3_‐modified systems (∼7 mS cm^−1^), polymer–ionic liquid hybrids (∼13 mS cm^−1^), and high‐flux GPEs (∼10^−2^ S cm^−1^) supporting ultrafast cycling. No single class dominates; each resolves different constraints on transport, stability or manufacturability. A coherent path forward lies in unifying three principles: (i) speciation control to stabilize $AlCl_{4}^{-}$/$Al_{2}Cl_{7}^{-}$ equilibria; (ii) robust interphase engineering to mitigate corrosion and deposition heterogeneity; and (iii) hybrid electrolyte architectures that integrate ionic liquid/DES functionality with aqueous, polymeric or solid matrices. Collectively, although not yet fully validated, these advances position aluminum‐ion chemistry as a scalable, reversible and sustainable contender for next‐generation large‐scale energy storage.

**TABLE 2:** Comparative evaluation of major Al‐ion electrolyte families.

Electrolyte	Advantages	Drawbacks	Sustainability	Practical application challenges
Ion	Wide ESW, chemical & thermal stability, high ionic conductivity, non‐volatile, low‐flammable	Costly, highly corrosive, water‐sensitivity, Al dendrites, high viscosity	Low to moderate—cost‐ and handling‐limited	Corrosion and moisture sensitive; requires dry processing, corrosion‐resistant current collectors, validated binder/ separator system and HCl/end‐of‐life management
Aqueous	High ionic conductivity, cheap, low viscosity, environmental benign	High polarization voltage, parasitic reactions, LiTFSI and Al(OTf)_3_ salts are expensive	High sustainability—safe and low‐cost	Low cost and safe, but HER/passivation/corrosion must be controlled; chloride‐containing systems require current‐collector screening.
HES	Electrochemical stability, dendrites inhibition, wide ESW, low viscosity, good ion transport	Narrower ESW compared to DES, residual water reactivity, not always compatible with Al metal anode	Moderate—composition‐dependent	Lower water activity and improved interface stability, but residual‐water reactivity and Al‐metal compatibility remain composition‐dependent.
DES	Electrochemical stability, dendrites inhibition, wide ESW	Water sensitivity, high viscosity; lower ionic conductivity than IL, potentially flammable	Moderate to high—accessible but viscous	Lower cost than ILs, but AlCl_3_‐based DESs can remain chloride‐corrosive and viscous.
Organic	High ionic conductivity, practical	Passivate Al	Low sustainability—flammability‐ and toxicity‐limited	Lower chloride corrosion but possible flammability/toxicity and Al passivation.
Polymer, gel & quasi‐solid	Good stability, dendrites inhibition, wide ESW	Low ionic conductivity, poor rate performance	Moderate sustainability—safe but material‐intensive	Leak‐free and safer form factor, but polymer‐AlCl_3_ interactions and lower conductivity require validation.

## 5 Operando and Computational Insights Guiding Design

Operando characterization techniques are essential for understanding the operating mechanisms of materials and electrolytes in electrochemical energy‐storage systems. In Al‐ion devices, where the chemistries of electrolytes and electrodes are diverse and often complex, these techniques are particularly critical for addressing fundamental questions: (i) which species carry charge in the electrolyte; (ii) which ions or complexes are intercalated into the cathode; (iii) what redox processes occur during cycling; and (iv) how do the SEI and cathode–electrolyte interphase (CEI) form and evolve over time [160, 161, 162]? As direct operando studies on AICs are rare, the current understanding of the mechanisms involved relies largely on evidence from Al‐ion batteries and hybrid Al‐based systems. This is justified because charge storage in many Al‐based devices is not purely capacitive; instead, it reflects a coupled interplay of surface‐controlled adsorption, pseudocapacitive redox and ion intercalation/deintercalation. Some architectures also exhibit conversion or plating/stripping contributions. Accordingly, selected battery‐focused operando studies are discussed here not as direct equivalents of AICs, but as mechanistic references that clarify electrolyte speciation, solvation/desolvation, interfacial evolution and structural dynamics in Al‐based systems, in which capacitive and battery‐type processes often coexist. A summary and critical analysis of operando characterization techniques are provided in Table 3.

**TABLE 3:** Overview of selected operando characterization techniques for electrochemical materials and electrolytes.

Technique	Object	Process studied	Spatial; Temporal resolution	Main limitations
XRD, synchrotron XRD	Crystal structure	Phase transformations, lattice strain, and (de)intercalation pathways during cycling	1–10 µm; s–min	Limited surface sensitivity; difficult operando cell design for liquid electrolytes. Requires synchrotron radiation
X‐ray absorption spectroscopy (XAS)	Local atomic environment	Redox states, local coordination, and structural disorder of electrode elements	∼0.1 Å; s–min	Requires synchrotron radiation; averages bulk signal; possible beam damage
Nuclear magnetic resonance (NMR)	Ion dynamics	Ion mobility, solvation, and electrolyte‐electrode interactions	Bulk; ms–min	Low sensitivity; limited for paramagnetic species; expensive setup
X‐ray / neutron tomography, STXM, TXM	3D structure and composition	Electrode cracking, gas evolution, and ion distribution in full cells	10–100 nm; s–min	Requires high‐flux sources; complex data reconstruction
Ambient‐pressure XPS	Surface chemistry	SEI/CEI composition, oxidation states, and electrolyte decomposition products	Depth: < 10 nm; min	Complex setup; limited pressure range; mainly model systems
Raman spectroscopy	Chemical bonding	Staging, electrolyte decomposition, SEI formation, ion solvation, and adsorbed species	< 1 µm (nm in tip‐enhanced RS); ms–s	Fluorescence and heating effects; low signal on rough electrodes
FTIR	Functional groups, molecular species	Solvent and additive decomposition, interphase growth, and functional group change	µm‐scale; s–min	Weak signals for dilute species; strong absorption by water and CO_2_
TEM / cryo‐TEM / operando TEM	Morphology	Particle cracking, dendrite formation, and nanoscale interphase layers	< 1 nm; ms–s	Beam damage; small sample region; vacuum environment
Electrochemical quartz crystal microbance(EQCM)	Mass change	Ion insertion/extraction and SEI growth kinetics	ms–s; mass sensitivity: ng cm^−2^	Only for thin films; no chemical identity of mass changes

### 5.1 Operando XRD/XAS/NMR for Speciation and Phase Tracking in Al Electrolytes

**FIGURE 15:**
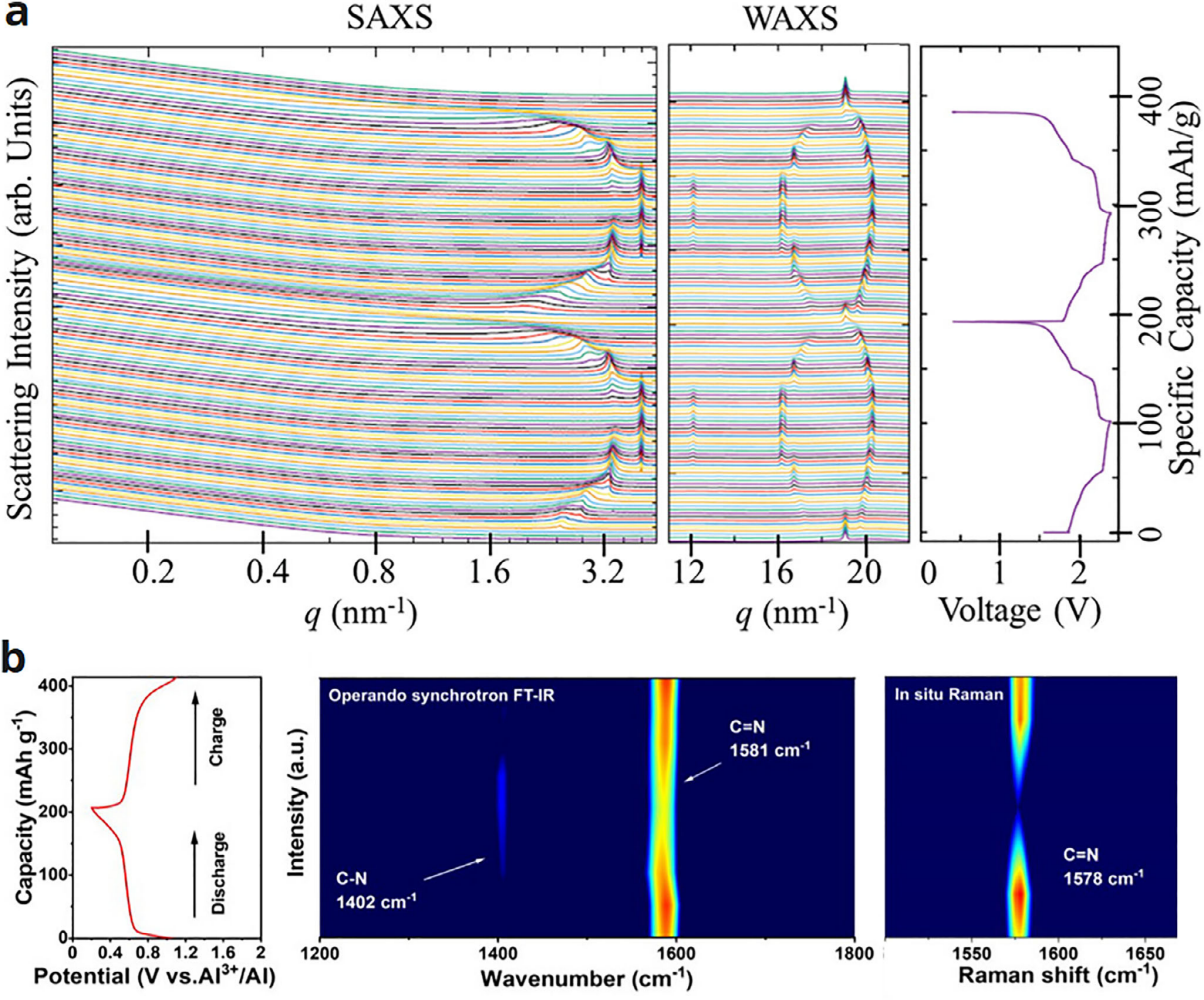
Operando analyses of Al‐based energy‐storage systems: (a) operando SAXS and WAXS monitoring of anion intercalation into graphite in an Al | graphite cell, shown together with the corresponding galvanostatic charge–discharge profiles; (b) operando synchrotron FTIR and Raman contour maps of an organic cathode during charge and discharge. Panels (a,b) are reproduced with permission from Refs. [167, 176].

Operando X‐ray techniques have become indispensable for investigating structural dynamics in aluminum‐ion and other multivalent‐ion storage systems, where strong Coulombic interactions and complex ion solvation frequently cause significant lattice distortions in cathode materials. These methods allow for the real‐time monitoring of interlayer spacing, phase transitions, and strain evolution under operating conditions, providing a direct link between electrochemical behavior and structural responses. Among these techniques, operando XRD is the standard method for monitoring stages of chloroaluminate and other anion intercalation into graphitic carbons [163, 164, 165]. In graphitic carbon intercalation compounds, a “stage” refers to the number of graphene layers that separate successive intercalant layers. For chloroaluminate anions, reversible operation up to stage 4 was observed at room temperature, and up to stage 3 at freezing temperatures [164]. Exfoliation of graphene layers occurs at higher (2 and 1) stages, leading to decomposition of the cathode [166]. By contrast, small angle X‐ray scattering (SAXS) extends this approach to cover a range of structural changes from nm to sub‐µm. The SAXS peaks directly correspond to interlayer expansion and staging (e.g., stage 6 → 4 → 3). The combined SAXS/wide angle X‐ray scattering (WAXS)N‐methylacetamide approach simultaneously reveals microporosity evolution and structural reorganization of graphite during cycling (while WAXS analysis is largely similar to XRD) [167]. Operando XRD can be readily combined with other X‐ray techniques owing to the compatibility of spectro‐electrochemical cell designs. Operando X‐ray tomography, complemented by XRD staging studies, has allowed simultaneous investigation of the structural and morphological evolution of the graphite cathode [168]. The 3D reconstructions revealed that natural graphite expands by 35%–40% upon charging and fully recovers after discharge, whereas highly ordered pyrolytic graphite expands by ∼180% and exhibits irreversible deformation. These results demonstrate that mechanical strain and volume expansion critically govern the reversibility of the intercalation process. Operando X‐ray absorption spectroscopy enables real‐time tracking of local electronic structures and oxidation states, clarifying cationic and anionic redox processes in layered oxides, sulfur‐based cathodes, and other complex materials. Operando X‐ray absorption spectroscopy (XAS) provides atomistic insight into anion coordination and packing within intercalated graphite. This method elucidates the working mechanism of aluminum‐ion batteries by monitoring reversible shifts in the Al and Cl K‐edge spectra during cycling, revealing changes in the local coordination environment consistent with the insertion and extraction of $AlCl_{4}^{-}\;$ anions into graphite layers [165, 169]. XAS techniques also allow tracking of composition changes in the electrolyte and redox states of the cathode active material [170, 171]. While X‐ray methods primarily probe solid‐state processes, nuclear magnetic resonance (NMR) provides complementary information [172]. Technical challenges have so far limited most of such measurements to ex situ studies, which provide information on liquid‐phase speciation and ion dynamics [173], as well as on the intercalation of Al cations [174] or chloroaluminate anions into solid phases [175]. Figure 15 summarizes the most useful operando characterization methods discussed in this review and illustrates how they provide complementary insight into the electrochemical processes of Al‐based energy‐storage systems.

### 5.2 Other Operando Techniques

Operando Raman spectroscopy is popular due to the relative simplicity and affordability of its setups and cells. It may or may not reveal changes in the electrode materials, depending on the cathode chemistry [177, 178]. It captures molecular‐level information with high temporal resolution from electrode surfaces and has been widely applied to monitor the charge–discharge processes in AIBs. For graphitic electrodes, Raman spectra reveal the evolution of intercalation stages, where the stage index (n) can be estimated at various states of charge and discharge. A characteristic feature frequently observed in operando Raman investigations of graphitic cathodes is the pronounced attenuation or complete disappearance of the E_2g2_(i) vibrational mode at elevated potentials, signifying the formation of graphite intercalation compounds with low stage indices [169, 179, 180]. However, operando Raman spectroscopy can be affected by surface effects due to co‐intercalation of cations and should therefore be interpreted carefully [181]. Optical microscopy can serve as a stand‐alone operando technique or complement Raman studies using the same setup, providing visual information on electrochemical processes such as gas evolution, kinetic limitations, or dendrite growth [58, 117, 182, 183, 184]. Operando FTIR is complementary to Raman spectroscopy and reveals changes in functional groups. It is particularly suitable for studying organic active materials and identifying electrolyte decomposition [176, 185]. In situ magnetometry can be used to study mechanisms in materials exhibiting magnetic moments, where it can reveal multicarrier charge‐storage behavior in FeSe_2_ cathodes for AlBs, involving co‐intercalation of Cl^−^ and $AlCl_{4}^{-}\;$ anions [186] during charging, while Al^3+^ cations intercalate during discharge [187]. EQCM measurements, performed simultaneously with electrochemical cycling, provide quantitative insight into mass changes at the electrode. For example, EQCM may reveal that instead of metallic Al plating, Al(OH)_2_^+^ and Al^3+^ ions are adsorbed and desorbed on the anode surface. In Al(OTf)_3_/N‐methylacetamide/urea electrolytes, electrode processes are dominated by ion adsorption and gas evolution rather than true Al plating [183]. In WO_3_ cathode, EQCM revealed that Al^3+^ intercalation is reversible only in dilute electrolytes, whereas high Al(ClO_4_)_3_ concentrations promote chelate‐like complex formation that destabilizes the SEI and leads to irreversible mass accumulation and degraded cycling stability [188].

### 5.3 Molecular Dynamics (MD)/Density Functional Theory (DFT) on Electrolyte Speciation, Transport, and Desolvation at Interfaces

DFT and other computational calculations have been widely used to evaluate the adsorption behavior, diffusion barriers, and charge transfer characteristics of chloroaluminate species such as $AlCl_{4}^{-}\;$ and $Al_{2}Cl_{7}^{-}$ on various electrode materials. While Al‐ion batteries typically involve bulk intercalation processes within host structures, AICs primarily rely on surface adsorption and fast pseudocapacitive reactions. Nevertheless, the fundamental interaction between the electrode surface and chloroaluminate ions remains similar in both systems. Therefore, computational studies performed for battery systems can also provide important insights into the ion–electrode interaction strength and charge storage kinetics in AICs.

In contrast to lithium‐ion systems, where the interplay between electrolyte chemistry and intercalation is comparatively well mapped, aluminum‐ion systems remain at a more fundamental stage of discovery. The identity of the active charge carriers −whether isolated Al^3+^, solvated Al‐bearing species, or multimeric complexes− remains electrolyte‐dependent and in many systems is still not fully settled, as do the details of solvation, desolvation, and interfacial redox. Computational methods have become indispensable for resolving details that are otherwise inaccessible experimentally. Classical molecular dynamics (MD) have been particularly valuable in characterizing bulk electrolyte behavior [189]. By calculating radial distribution functions (RDFs), coordination numbers, and ligand residence times, MD allows direct mapping of aluminum speciation and ion–ion interactions [190, 191]. Ion‐pair lifetimes and spatial correlations further reveal the persistence of strongly bound species [192]. Yet the fidelity of these predictions depends critically on the force fields employed, which can be problematic for highly charged, polarizable ions such as Al^3+^ [193], as classical MD simulations approximate ions as charged van der Waals spheres. In such cases, polarizable force fields, such as atomic multipole optimized energetics for biomolecular applications, can help address this problem. To further address these challenges, ab initio molecular dynamics (AIMD) simulations with density functional theory (DFT) potentials have been increasingly employed [194, 195]. These methods capture polarization, bond breaking and formation, and ligand exchange events in full electronic detail, enabling direct validation of coordination motifs observed in classical simulations. AIMD in particular has proven invaluable in capturing early stages of desolvation or adsorption at electrode surfaces, where electronic redistribution cannot be neglected [196, 197, 198, 199]. However, despite advances in high‐performance computing, such simulations remain computationally very demanding, which restricts them to relatively small models and short simulation timescales, typically on the order of only a few nanoseconds. Beyond equilibrium structure, enhanced‐sampling techniques such as umbrella sampling and metadynamics have enabled reconstruction of free‐energy landscapes for desolvation, ligand exchange, and ion adsorption, while nudged elastic band (NEB) calculations at the DFT level quantify the activation barriers associated with these processes [200, 201, 202]. More recently, reactive force fields and especially machine‐learned interatomic potentials trained on DFT data have begun to bridge the gap between the accuracy of electronic‐structure methods and the statistical sampling capability of MD, offering a new route to simulate reactive events with near‐DFT fidelity over nanosecond timescales [203, 204].

Unlike lithium electrolytes, where transport proceeds largely through solvated cations, aluminum transport in ionic liquids is dominated by multimeric chloroaluminate complexes such as $AlCl_{4}^{-}\;$ and $Al_{2}Cl_{7}^{-}$. Yang and colleagues provided a critical design principle by modifying the imidazolium cation structure [205]. Their experimental studies, supported by simulation, revealed that substituents in 1‐methyl‐3‐propylimidazolium weaken the strong Coulombic binding between the cation and anions, thereby accelerating the intercalation of $AlCl_{4}^{-}$ and improving the efficiency of electrodeposition and stripping. From a computational perspective, this effect manifests in RDFs and ion‐pair lifetime analyses, which show reduced persistence of cation–anion binding motifs. Such studies highlight the active role of organic cations, which are often assumed to be inert, in modulating both solvation and transport. Kosar and co‐workers extended these findings through detailed MD simulations of AlCl_3_− [EMIm]Cl electrolytes, exploring both neat and solvent‐modified systems [190]. They demonstrated that the addition of co‐solvents such as dichloromethane (DCM) and toluene dramatically decreases viscosity, enhances diffusion coefficients, and improves ionic conductivity. Crucially, the simulations showed that these solvents also influence speciation equilibria by facilitating the formation of electroactive $AlCl_{4}^{-}$ and $Al_{2}Cl_{7}^{-}$ species in acidic regimes. DCM, in particular, was shown to reduce viscosities and increase diffusion coefficients to a greater extent than toluene. Importantly, the MD‐derived transport coefficients and densities agreed quantitatively with experimental measurements, underscoring the predictive capability of well‐parameterized force fields. These results illustrate that solvents are not merely inert diluents but active participants in tuning speciation and nanostructure of electrode material, thereby mitigating the kinetic limitations inherent to viscous ionic liquids. The complexity of aluminum speciation extends beyond chloride frameworks. Eiden and colleagues performed one of the first combined computational and crystallographic studies of AlCl_3_ mixtures with [BMP]TFSI and [EMIm]TFSI [206]. Their work revealed that AlCl_3_ engages in extensive ligand exchange with the [TFSI]^−^ anion, generating species such as $AlCl_{4}^{-}$, neutral Al(TFSI)_3_, and transient [AlCl_2_(TFSI)_2_]^−^ complexes. DFT thermodynamic calculations indicated that at low AlCl_3_ concentrations, [TFSI]^−^ coordination dominates, but at higher loadings a biphasic regime emerges with one phase rich in Al(TFSI)_3_ and another in $AlCl_{4}^{-}$. Electrodeposition of aluminum was proposed to proceed via transient [AlCl_2_(TFSI)_2_]^−^ at the electrolyte/electrode interface. The identification of such intermediates was made possible by a combination of DFT reaction profiling and crystallographic confirmation of Al(TFSI)_3_. This study emphasized that reactive, short‐lived species often invisible to bulk measurements, can be the decisive actors in aluminum electrodeposition. Further evidence of the decisive role of solvation comes from molecular solvents. Reed and colleagues examined aluminum triflate in diglyme, using DFT calculations to map coordination energetics [207]. They found that Al^3+^ forms overwhelmingly stable bis‐diglyme chelate complexes, $Al{\left(diglyme\right)}_{2}^{3+}$, stabilized by free energies near −1922 kJ mol^−1^. Triflate counter‐ions interact primarily through coulombic association rather than covalent bonding, reinforcing the dominance of the chelated cation. Electrochemical measurements revealed that the electrochemical window narrowed to ∼5.5 V at intermediate salt concentrations but widened beyond 8 V at higher loadings. These results suggest that the stability of chelation, while enhancing conductivity, may paradoxically hinder dissolution at the electrode surface, adding an additional layer of complexity to the design of molecular electrolytes. Taken together, these computational and experimental investigations paint a picture of aluminum speciation as dynamic and context‐dependent, controlled by cation substitution, solvent identity, anion chemistry, and concentration regime. Increasingly, simulations validated by experiment have shifted from retrospective explanation to predictive design, suggesting that electrolyte engineering for aluminum‐ion batteries may soon be guided by computation at every stage.

A representative case is the computational investigation of aluminum intercalation in vanadium dioxide (VO_2_), long considered a prototype host for so‐called “super‐valent” energy‐storage devices [208]. Using the CASTEP package, researchers modeled the energetics, lattice response, and electronic structure associated with progressive Al insertion. The calculations showed that Al ions preferentially occupy one‐dimensional channels formed by distorted VO_6_ octahedra, with consistently negative formation energies for Al_x_(V_4_O_8_) up to x = 2, confirming the thermodynamic feasibility of initial intercalation. Beyond this threshold, however, insertion induced pronounced lattice distortions. For instance, VO_6_ octahedra elongated, V‐O bonds stretched beyond 3 Å, and effective bond breaking was observed. The introduction of three or more Al ions expanded the unit cell volume by ∼16% along the b and c axes while compressing the a‐axis, a response attributed to intense coulombic interactions and local screening effects. Importantly, substitutional incorporation of Al at V sites was computed to be energetically unfavorable (ΔE > 0), ruling out this pathway. These results indicate that while Al^3+^ intercalation is thermodynamically possible, the high charge density imposes severe lattice strain, destabilizing the host at higher concentrations. The broader implication is that host structures for aluminum‐ion storage must provide not only energetically accessible insertion sites but also frameworks that can accommodate and buffer large strain. The VO_2_ case study highlights how DFT‐based modelling quantifies the interplay between insertion energetics and structural integrity, directly linking to experimental reports of ∼165 mAh g^−1^ initial capacity with retention of 116 mAh g^−1^ after 100 cycles, albeit at a modest energy density of ∼48 Wh kg^−1^. Such computational/experimental synergies point toward rational cathode design principles. Transition metal oxides remain promising candidates because their variable oxidation states can be coupled with open frameworks to sustain charge compensation. Structures with layered arrangements, wide tunnels, or defect‐tolerant backbones are especially attractive, as they can buffer the local strain imposed by trivalent carriers while preserving electronic conductivity. Building on these insights, recent work has extended the computational exploration of aluminum ions beyond oxides to transition‐metal chalcogenides, where the interplay of metal and anion redox introduces new electrochemical possibilities [209]. The diffusion barrier and adsorption of electrolyte species play a critical role in selecting a material. A systematic study of cubic Co‐based oxides, sulfides, and selenides (Co_3_O_4_, Co_3_S_4_, and CoSe_2_) provides a striking example of how DFT supports the selection of materials for capacitor/battery electrodes. DFT slab models were used to calculate adsorption energies of the electroactive $AlCl_{4}^{-}$ carrier on different crystallographic facets, revealing values of –2.21, –2.13, and –3.51 eV for Co_3_O_4_ (311), Co_3_S_4_(220), and CoSe_2_(210), respectively. To quantify transport, climbing‐image nudged elastic band (NEB) methods were applied, yielding diffusion barriers of 0.368 eV for Co_3_O_4_, 1.38 eV for Co_3_S_4_, and only 0.137 eV for CoSe_2_. These results showed that while CoSe_2_ binds $AlCl_{4}^{-}$ most strongly, it also permits facile diffusion across its surface, uniquely combining stability and mobility. The computational predictions were borne out experimentally. Electrochemical testing showed that CoSe_2_ delivers two distinct discharge plateaus at 0.8 and 1.8 V, with an initial capacity of 785.6 mAh g^−1^ far surpassing Co_3_O_4_ (388.3 mAh g^−1^) and Co_3_S_4_ (579.7 mAh g^−1^). However, downsizing these materials to nanoparticles could make the discharge behavior more linear, enabling their use in supercapacitor applications. The study thereby demonstrated how surface adsorption energies and diffusion barriers can help rationalize trends in macroscopic performance when interpreted together with morphology, conductivity, electrolyte chemistry, and side reactions. First‐principles simulations not only capture these competing effects but also provide mechanistic clarity that experimental observables alone cannot resolve. When combined with electrolyte modelling, such insights offer a pathway to rationally design both carrier speciation and host architectures, thereby advancing aluminum‐ion batteries toward practical implementation.

**FIGURE 16:**
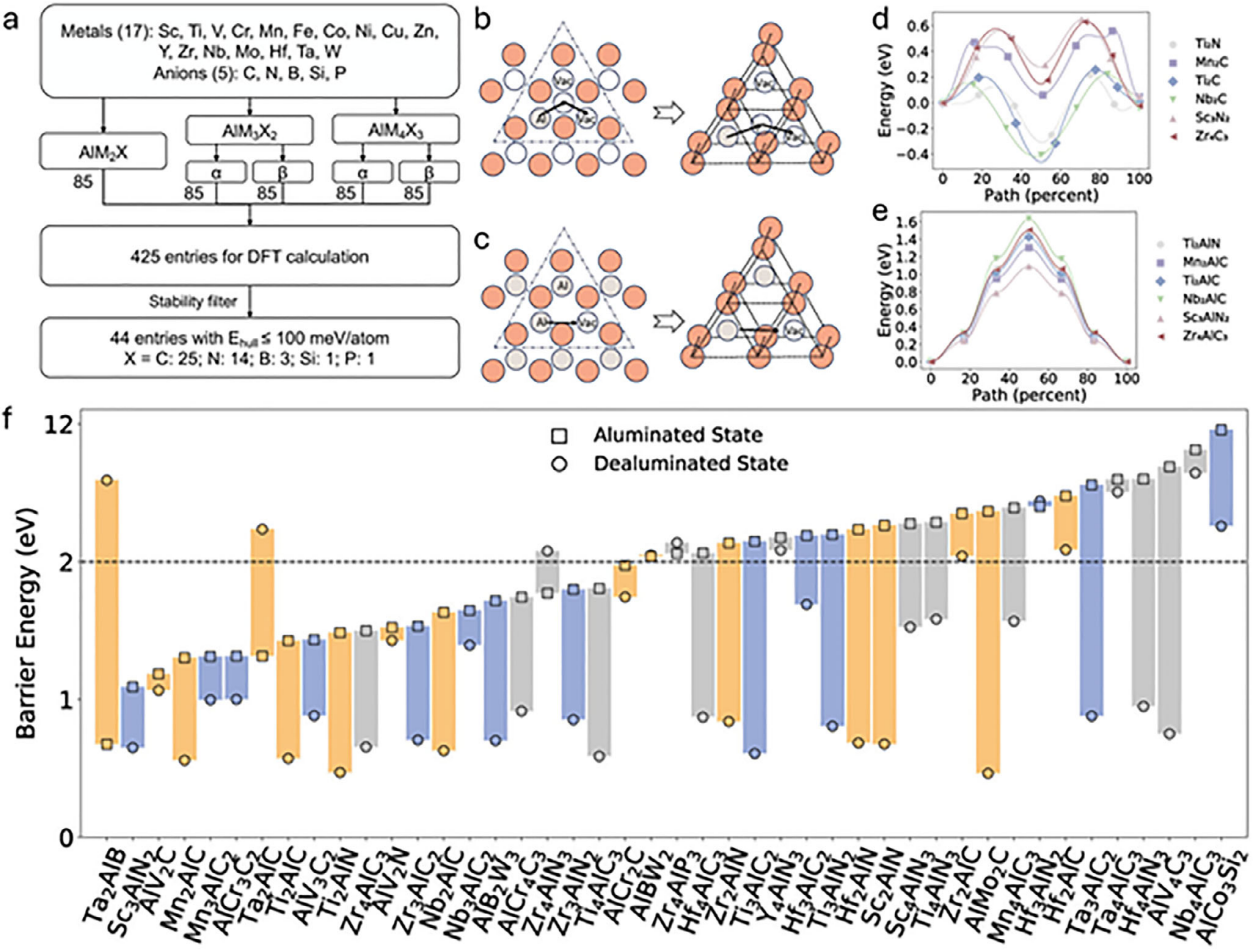
(a) High‐throughput calculations workflow (In total 425 MAX entries were evaluated. 44 entries were found with energy above the hull (*E*_hull_) ≤100 meV atom^−1^), Al migration pathway in MAX through (b) dealuminated state; (c) aluminated state (The left panels show the top view of diffusion pathway while the right panels show a perspective view; Al, Metal, and vacancy are shown as grey, orange, and white spheres). Energy along the migration pathway in different Al‐vacancy environments: (d) dealuminated state; (e) aluminated state, (f) A vertical bar chart showing the activation barrier energy for a single vacancy diffusion (aluminated state) and a single Al diffusion (dealuminated state) for the calculated stable/metastable MAX phases: AlM_2_X, orange; AlM_3_X_2_, blue and AlM_4_X_3_, gray. Square represents the energy for aluminated state and circle shows that of dealuminated state. The y scale above 2 eV is reduced to show better the barriers within 0–2 eV (Reproduced from Ref. [211], with permission from Wiley‐VCH, Copyright 2023).

Unlike conventional layered oxides or sulfides, MAX phases represent a distinctive class of electrode candidates because of their mixed metallic‐ceramic bonding, which endows them with structural robustness, electronic tunability, and a capacity to sustain mechanical resilience under repeated electrochemical cycling [210]. In a recent computational survey, more than 400 possible MAX compositions were enumerated, with 44 identified as thermodynamically stable or metastable within an energy hull (E_hull_) threshold of ≤100 meV atom^−1^ for supercapacitor/battery electrodes (Figure 16a) [211]. This large‐scale enumeration provides a roadmap for future experimental validation and highlights design levers not typically available in conventional host lattices. One particularly compelling insight is the role of vacancy‐assisted aluminum diffusion (Figure 16b). Unlike lithium‐ion systems, where interstitial channels dominate transport, aluminum mobility in MAX phases is strongly facilitated by the creation of Al vacancies during cycling. These vacancies act as local diffusion highways, significantly lowering migration barriers in dealuminated states and providing a potential design knob to tune kinetics through controlled vacancy engineering. Equally striking is the observation that stacking polymorphs exert a critical influence on transport energetics. Most MAX compounds favored α‐stacking, yet a substantial subset stabilized into β‐stacking configurations, where the relative positioning of Al atoms altered migration energetics by several hundred meV. This polymorph‐dependent behavior suggests that synthetic control of stacking order achieved through precursor selection, growth kinetics, or annealing conditions could serve as a powerful strategy to tune ionic conductivity in real systems. Notably, a subset of compositions including V_2_C, Ti_2_C, Ti_2_N, and Mn_2_C displayed calculated energy densities on par with or exceeding state‐of‐the‐art carbonaceous and organic electrodes, underscoring their dual advantage of high theoretical capacity and mechanical resilience.

Nevertheless, the gap between computational predictions and experimental realization remains a central bottleneck. The E_hull_ cut‐off used to define metastability offers a useful metric for computational screening, yet real‐world synthesizability depends strongly on kinetic factors, precursor chemistry, and processing pathways. Importantly, MAX phases provide not only a direct pathway as bulk aluminum host but also an indirect one. They can be chemically etched to produce 2D MXenes, which offer even greater ion accessibility. Prior experimental reports on V_2_CT_x_ MXene cathodes validate these computational predictions, showing enhanced capacity and reversibility when dimensionality is reduced, directly aligning with the role of vacancy‐assisted diffusion highlighted in simulations. The conceptual connection to lithium‐ion transport mechanisms offers further insight. Just as Li diffusion can be facilitated by tri‐vacancy clustering, Al^3+^ migration in MAX phases exploits vacancy‐assisted channels to overcome its otherwise sluggish kinetics. However, the higher charge density of Al^3+^ typically imposes severe electrostatic penalties, which in most electrode families translate into poor mobility. That MAX phases can circumvent part of this challenge through structural and vacancy engineering is significant. Among the hundreds of candidates surveyed, 18 compositions exhibited diffusion barriers below 2 eV atom^−1^, marking them as prime candidates for near‐term synthesis and testing (Figure 16f). In another report, Liu et al. developed a forward prediction and screening framework integrating materials databases, auto‐encoding descriptors, and convolutional neural networks (CNNs) to accelerate discovery of high‐performance AIB cathodes [212]. Using the Materials Project database containing over 4,300 compounds, coulomb matrices were constructed through an auto‐encoding scheme and processed as two‐dimensional inputs for CNN training. The model was optimized to predict five key electrochemical attributes, especially average voltage, gravimetric capacity, gravimetric energy, stability upon charge, and stability upon discharge with mean absolute errors of 0.17 V, 18.95 mAh g^−1^, and 47.34 Wh kg^−1^, respectively, demonstrating near‐DFT accuracy at vastly reduced computational cost. Focusing on trivalent‐ion systems, the authors applied the trained framework to screen potential AIB cathode materials, yielding eight promising candidates (V_2_O_5_, CrO_2_, FeF_3_, MnO_2_, CoF_3_, NiF_2_, TiS_2_, and WO_2_) that satisfy stringent performance thresholds (average voltage ≥ 3 V, capacity ≥ 250 mAh g^−1^, and gravimetric energy ≥ 1,000 Wh kg^−1^). These selections exhibit high theoretical capacities and structural robustness toward Al^3+^ intercalation, aligning closely with DFT results. Notably, WO_2_ was additionally identified by the machine learning model but not by DFT screening, illustrating the method's predictive generalization and its ability to capture subtle structure/property correlations beyond conventional descriptors. The error distribution analysis indicated that deviations in stability predictions arise primarily from incomplete crystallographic information rather than intrinsic model limitations. Overall, the study demonstrates that data‐driven approaches can reliably approximate DFT‐level predictions for multivalent systems, offering a computationally efficient route to identify and optimize cathode frameworks capable of accommodating the high charge density of Al^3+^ ions. In another report, a recent first‐principles investigation by Ahmad et al. elucidates the defect‐mediated electrochemical response of aluminum nitride monolayers for supercapacitor electrodes within a DFT framework. Pristine aluminum nitride nanosheets exhibit a wide indirect bandgap (∼2.93 eV), resulting in negligible density of states density of statesat the Fermi level and consequently suppressed quantum capacitance (CQ). However, introduction of mono‐ and divacancies generates localized impurity states that substantially enhance charge accommodation. In particular, Al‐vacancy engineering yields a maximum quantum capacitance of ∼690 µF cm^−2^ at +0.06 V with a surface charge density of ∼357 µC cm^−2^, indicating strong anodic suitability. Conversely, N‐vacancies induce n‐type characteristics with CQ values up to ∼313 µF cm^−2^ in the negative bias regime, favoring cathodic functionality. The Al–N divacancy configuration demonstrates intermediate yet significant CQ (∼438 µF cm^−2^), reflecting dual donor–acceptor behavior. These findings highlight vacancy‐driven band structure modulation as an effective strategy to optimize Al‐based 2D electrodes for high‐performance Al‐ion supercapacitor architectures [213].

Recent atomistic modeling has revealed that the superior performance of graphene flakes arises not from a single mechanism but from a dual‐storage process in which both intercalation and covalent grafting of chloroaluminate species occur in tandem [214]. Whereas pristine graphite primarily relies on van der Waals‐driven intercalation of $AlCl_{4}^{-}$, expandable graphene introduces defects and functional groups that act as covalent anchoring sites, profoundly modifying its electronic density of states. At optimal grafting concentrations, these graphene derivatives undergo a transition from semimetallic to metallic conduction, enabling the large electron fluxes required for multivalent redox processes. This dual mechanism confers several electrochemical advantages. Covalently grafted species stabilize the electrode by distributing strain heterogeneously, while intercalated species expand the interlayer spacing and induce in‐plane corrugations that, rather than disrupting transport, generate flexible microenvironments conducive to ion migration. The coexistence of intercalated and grafted units produces a heterogeneous potential energy landscape that prevents ion trapping and creates multiple low‐barrier diffusion pathways, in sharp contrast to the rigid environments of crystalline intercalation hosts. The computational prediction of orientation‐dependent binding energetics, where certain intercalation geometries are far more favorable than others, provides a mechanistic explanation for experimental findings that expandable graphene maintains high conductivity even at elevated grafting levels, conditions under which pristine graphite typically suffers from kinetic slowdown and rapid capacity fade. Together, the MAX/MXene and graphene‐flake case studies illustrate two complementary approaches to overcoming the fundamental challenges of multivalent‐ion storage, namely, vacancy‐assisted transport in robust, tunable frameworks and synergistic multi‐mechanism storage in structurally adaptive carbons. Both directions highlight the central role of computational insights in revealing hidden mechanisms, whether vacancy‐controlled diffusion channels or orientation‐dependent grafting energetics that would be difficult to extract experimentally alone. By linking atomistic energetics to macroscopic cycling stability, these studies strengthen the design principles guiding aluminum‐ion batteries: wide channels, mechanically resilient hosts, and flexible frameworks are essential for reconciling the severe charge density of Al^3+^ with the need for high reversibility and capacity retention.

In summary, this integrated multiscale operando analysis, combined with DFT computations, enables precise identification of gallery geometries and intercalant arrangements, correlating structural parameters with operating potential and temperature.

Computational approaches have become indispensable in unraveling the atomic‐scale mechanisms that govern electrolyte speciation, ion transport, and interfacial processes in aluminum‐ion batteries. Classical and ab initio molecular dynamics have revealed the dynamic equilibria of chloroaluminate species, particularly $AlCl_{4}^{-}$ and $Al_{2}Cl_{7}^{-},$ and their dependence on electrolyte acidity, viscosity, and co‐solvent environment. DFT‐based methods have further quantified adsorption energies (–2.2 to –3.5 eV), diffusion barriers (0.1–1.38 eV), and desolvation energetics at metal and carbon interfaces, elucidating the kinetic bottlenecks limiting Al^3+^ mobility. These simulations provide a mechanistic basis for interpreting redox behavior and interfacial stability, complementing experimental trends such as improved diffusion in solvent‐modified ionic liquids and enhanced reversibility at optimized AlCl_3_:ionic liquid ratios. Machine‐learning and data‐driven frameworks trained on large computational databases have extended this predictive capability, identifying a small subset of high‐capacity candidates from over 4000 screened materials. These efforts mark a transition from retrospective explanation to true forward design, where theoretical insights now guide targeted synthesis and performance validation. Building on the atomistic understanding offered by computational modeling, operando techniques extend this perspective into the experimental domain, resolving how these processes unfold dynamically during electrochemical cycling. In AIBs, operando XRD and SAXS/WAXS have captured dynamic structural transitions and lattice expansions during $AlCl_{4}^{-}$ intercalation, while XAS measurements have traced reversible Al–Cl coordination shifts and electronic structure evolution across charge–discharge cycles. Raman spectroscopy provides complementary temporal resolution, identifying phase transitions. Together, these operando findings reveal that the reversibility and rate performance of AIBs are intimately tied to the mechanical strain tolerance of the host and the dynamic solvation–desolvation equilibria in the electrolyte. While computational models provide the predictive foundation for material and electrolyte selection, operando techniques validate and refine these predictions under realistic conditions. Future progress will rely on extending time‐resolved, multi‐modal operando measurements such as XRD, XAS, and Raman in tandem with EQCM and tomography, to map coupled structural, chemical, and mechanical responses, while concurrently developing multiscale simulation frameworks capable of capturing polarization, electron transfer, and solvation dynamics across relevant time and length scales. For aluminum‐ion batteries, such dual advancement will be essential for establishing transferable design principles for high‐capacity, long‐life, and chemically stable multivalent energy systems.

## 6 AICs in the Context of Other Multivalent Ion Hybrid Capacitors

Multivalent‐ion hybrid capacitors have attracted considerable attention owing to their low cost, intrinsic safety, high energy density, and the natural abundance of multivalent ion resources (Table 1). Among multivalent‐ion hybrid capacitors, Al^3+^ ion‐based SCs often demonstrate superior performance, primarily due to aluminum's higher charge density, which results in markedly higher theoretical volumetric and gravimetric capacities compared with their divalent counterparts, such as Mg^2+^, Ca^2+^, and Zn^2+^ (Figure 3). The high charge density of Al^3+^ facilitates stronger electrostatic interactions with surface functional groups and redox‐active sites, enabling pronounced faradaic behavior, thereby enhancing the overall capacitance. For example, a PEDOT:PSS electrode supported on carbon cloth exhibited a significantly higher specific capacitance in an aqueous Al_2_(SO_4_)_3_ electrolyte (269 F g^−1^) compared with MgSO_4_ (90 F g^−1^) at 0.2 A g^−1^ in a three‐electrode system [103]. This enhanced performance is attributed to the shorter first‐shell Al–O distance in [Al(H_2_O)_6_]^3+^ and smaller bare ionic radius (*R*_Al_^3+^ = 0.55 Å) relative to [Mg(H_2_O)_6_]^2+^ (*R*_Mg–O_ = 2.10 Å, *R*_Mg_^2+^ = 0.76 Å), which facilitates faster ion migration through the PEDOT:PSS matrix. The more compact first hydration shell of Al^3+^ may facilitate transport through the active PEDOT:PSS channels, promoting bulk diffusion‐controlled redox processes and yielding high capacitance. In contrast, the larger hydrated Mg^2+^ ions predominantly adsorb onto the electrode surface, resulting in a surface‐controlled capacitive mechanism with comparatively lower charge storage capability. Similarly, when ZIF‐67 MOF [99] was employed as a host cathode material in 0.5 M AlCl_3_ electrolyte, it exhibited a substantially higher charge storage capacity (∼180 mAh g^−1^) compared with MgCl_2_ (∼55 mAh g^−1^) at 0.5 A g^−1^. This improvement is primarily due to the smaller steric radius of Al^3+^ ions compared with Mg^2+^, which enables more facile and reversible ion insertion/extraction of Al^3+^ ions within the ZIF‐67 framework.

In recent studies, various electrode materials including MXenes and transition‐metal oxides have been explored in divalent ion‐based electrolytes such as Ca^2+^ and Zn^2+^ for SC applications [215, 216, 217, 218, 219]. However, full cell devices assembled with these divalent‐ion electrolytes typically deliver relatively low energy densities (15–35 Wh kg^−1^) compared with those employing Al^3+^‐based electrolytes (> 80 Wh kg^−1^) (Table 4). This substantial difference in the energy output has been mainly attributed to the higher charge density and smaller ionic radius of the Al^3+^ ion compared with divalent Ca^2+^ and Zn^2+^ ions listed in Table 1. Certainly, metrics also depend on voltage window, solvation structure, mass loading, active‐material utilization, and electrolyte chemistry. The strong electrostatic interaction between Al^3+^ ions and the host electrode materials enhances charge accumulation and promotes charge storage capability. Moreover, the the ionic radius of Ca^2+^ and Zn^2+^ are larger than that of Al^3+^, which results in sluggish ion diffusion kinetics within the electrode material. Consequently, divalent‐ion systems often exhibit surface‐controlled capacitive behavior with limited charge storage capacity. In contrast, the smaller radius of Al^3+^ facilitates faster ion transport and efficient intercalation within the electrode material, enabling diffusion‐controlled redox processes and multielectron transfer reactions. These properties also depend on the on the strength and size of the hydration shells. Additionally, Al‐based electrolytes generally provide a wider ESW and facilitate reversible multielectron redox reactions (a three‐electron transfer process that enables more charge to be stored per ion compared with divalent cations such as Mg^2+^, Ca^2+^, and Zn^2+^), contributing to enhanced energy density compared with the divalent ions. The comparative electrochemical performance of AICs compared with divalent ions (Zn^2+^, Mg^2+^, and Ca^2+^) capacitors are summarized in Table 4.

**TABLE 4:** State of the art and comparative table for energy‐storage performance of Al‐based systems and other multivalent‐ion supercapacitors/capacitors, and mechanism/device categorization. (C_g_: gravimetric capacitance; I_d_: current density; E_g_: gravimetric energy density; P_g_: gravimteric power density).

Nos.	Electrode material(s)	Mechanism / Device category	Electrolyte; voltage (V)	C_g_ (F g^−1^) @ I_d_ (A g^−1^)	E_g_ (Wh kg^−1^) @ P_g_ (W kg^−1^)	C_g_ retention @ cycles, cycling rate (A g^−1^)	Refs.
1.	Quinone functionalized hexaazatriphenylene‐based COF (HAQ‐COF)	Pseudocapacitive symmetric / organic‐redox COF supercapattery	1 M MgSO_4_; 1.5 V	58.1 mAh g^−1^ @ 0.2 A g^−1^	26.5 @ 3750	82 % @ 1000, 1.0	[220]
2.	K^+^ intercalated MnO_2_ (K‐MnO_2_//AC)	Hybrid: battery‐type MnO_2_ cathode // capacitive AC	1 M MgSO_4_; 1.8	189.4 @ 0.4	85.2 @360	96.7 % @ 20 000, 5.0	[221]
3.	β‐MnO_2_ (β‐MnO_2_//AC)	Hybrid: battery‐type β‐MnO_2_ cathode // capacitive AC	1 M MgSO_4_; 1.8	132.8 @ 0.2	60.8 @180	74.1 % @ 3500, 2.0	[222]
4.	Co‐doped MnO_2_ (Co‐MnO_2_//AC)	Hybrid: battery‐type Co‐MnO_2_ cathode // capacitive AC	1 M MgSO_4_; 1.7	191.8 @ 0.4	79.6 @360	94.8 % @ 15 000, 5.0	[223]
5.	NiCo‐LDH//VS_2_	Pseudocapacitive / faradaic asymmetric device; no metal plating	1 M MgSO_4_; 2.0	94.97 mAh g^−1^ @ 1 A g^−1^	48.44 @ 937.49	40 % @ 500, 5.0	[224]
6.	Ti_3_C_2_T_x_ MXene‐ Perylene Diimide (Ti_3_C_2_T_x_@cPDI//AC)	Hybrid: pseudocapacitive organic‐MXene electrode // capacitive AC	1 M CaCl_2_; 1.6	87 @ 1.0	31.0 @ 700	80% @ >10 000, 20 mV/s	[215]
7.	Vanadium carbide MXene (V_2_CT_x_//V_2_CT_x_)	Pseudocapacitive / intercalation‐type symmetric MXene device	5 M Ca(TFSI)_2_; 1.4	88.0 @ 0.5 (−0.6 – 0.7 V vs. Ag/AgCl	16.0 @ 1054	70% @ 10 000, 20 mV/s	[216]
8.	Sodiated manganese oxide (NMO//AC)	Hybrid: battery‐type NMO electrode // capacitive AC	0.5 M Ca(NO_3_)_2_; 2.0	54.0 @ 0.2	30.6 @ 200	96% @ 10 000, 1.0	[219]
9.	Vanadium carbide—nickel hexacyanoferrate (V_2_CT_x_—NiHCF)	3‐electrode intercalation/pseudocapacitive electrode‐level test	5 M Ca(TFSI)_2_; (‐1.0 to 0.7; 3‐el)	140.0 @ 0.5	—	87% @ 1000, 20 mV/s	[225]
10.	δ‐MnO_2_@carbon cloth //MXene@cotton cloth (δ‐MnO_2_@CAC //MXene@COC)	Hybrid: battery‐type MnO_2_ electrode // MXene/carbon electrode	2 M ZnSO_4_ and 0.1 M MnSO_4_; 1.9	172.0 @ 1 mV/s (CV)	26.8 @ 3838	∼80.7% @ 16 000, 5.05	[217]
11.	ZnxMnO_2_/PPy (ZMOP//PCNTs)	Hybrid: battery‐type ZnxMnO_2_/PPy electrode // capacitive PCNTs	2 M ZnSO_4_ + 0.1 M MnSO_4_; 1.9	109 @ 0.1	20.0 @ 867	86.4% @ 5000, 0.2	[218]
12.	MnO_2_//AC	Hybrid: battery‐type MnO_2_ electrode // capacitive AC	2 M ZnSO_4_ + 0.5 M MnSO_4_; 2.0	83.8 mAh g^−1^ @ 0.1	58.6 @ NA	61.8 % @ 5000, NA	[226]
13.	Zeolite imidazole‐based MOF (ZIF 67//ZIF 67 derived porous carbon)	Hybrid: MOF battery‐type/pseudocapacitive electrode // porous carbon; redox mediator	0.5 M AlCl_3_ + 6 mM KFCN; 1.7	50 mAh g^−1^ @ 1.0	86.0 @ 2000	88% @ 500, 2.0	[99]
14.	Remolded activated highly ordered and compact porous carbon (RHOPC) (RHOPC//RAT‐Ti_3_C_2_T_x_)	Pseudocapacitive rocking‐chair AIC; remolded carbon/MXene electrodes	1 M Al_2_(SO_4_)_3_; 2.0	—	112 Wh/L @ 750 W/L	91.8 % @ 10 000, 5.0	[27]
15.	Graphene oxide‐based carbon electrode (GO‐K‐P‐W//GO‐K‐P‐W)	Mixed EDLC/pseudocapacitive Al‐complex‐ion symmetric carbon device	0.3 M AlCl_3_ in EMIMBF_4_; 3.5	549 @ 0.5	233 @ 17500	77% @ 10 000, 2.0	[34]
16.	nitrogen‐doped micro‐mesoporous carbon sphere (NCS)// Al	Hybrid: Al metal deposition/stripping // N‐doped carbon capacitive/pseudocapacitive cathode	1.3:1 ratio of AlCl_3_:[EMIm]Cl; 2.2	224 mAh g^−1^ @0.3	—	100% @35 000, 10.0	[55]
17.	PPy@MoO_3_//AC	Hybrid: battery‐type PPy@MoO_3_ intercalation electrode // capacitive AC	Al_2_(SO_4_)_3_: 1.5	693 @ 1	30 @2840	93% @1800, 2	[67]
18.	SWCNT/W_18_O_49_// SWCNT/PANI	Pseudocapacitive/intercalation asymmetric oxide‐polymer device; no metal plating	1.0 M AlCl_3_: 1.8	0.586 F cm^−2^ @ 26 mA cm^−2^	19.0 mWh cm^−3^ @ 295 mW cm^−3^	95.9% @6000, 14 mA cm^−2^	[66]
19.	Mesoporous Carbon V_2_O_5_// Mesoporous Carbon V_2_O_5_	Pseudocapacitive symmetric V_2_O_5_/carbon composite device	1 M Al_2_(SO_4_)_3_; 1.6	290 @0.5	18.0 @147	88% @10 000, —	[71]
20.	Nb_2_CT_x_ on CC //CNT on CC	Hybrid/pseudocapacitive MXene electrode // capacitive CNT electrode	0.5 M Al_2_(SO_4_)_3_: 1.7	61.0 @1.0	24.7 @3.400	90% @4000, 2.0	[32]
21.	electrodeposited aluminum//graphene	Hybrid: Al metal deposition/stripping // graphene capacitive cathode	1:2 [EMIm]Cl‐AlCl_3_; 2.3	221.0 @3.0	151.0 @3390	87% @5000, 3.0	[98]
22.	N‐F co‐doped nanoporous carbon (NPC)//Al	Hybrid: Al metal deposition/stripping // mixed EDLC‐pseudocapacitive N/F‐doped carbon cathode	0.5 M AlCl_3_ in EMImCl; 1.7	∼197 mAh g^−1^ @ 0.5	—	Capacity fading within 25–50 cycles; exact retention not stated	[69]
23.	MWCNTs//Al	Hybrid: Al metal deposition/stripping // EDLC‐dominant MWCNT cathode with limited chloroaluminate intercalation	AlCl_3_‐[EMIm]Cl 1.3:1; 2	55 @ 0.15	—	82.4% @ 3500, 0.4	[94]
24.	Pore‐size‐controlled activated carbon (AC)//Al	Hybrid: Al metal deposition/stripping // EDLC‐dominant AC cathode	AlCl_3_‐[EMIm]Cl1.8:1; 2	79.6 @ 0.1	51 Wh kg^−1^ @ 91 W kg^−1^; 28 mWh cm^−3^ @ 50 mW cm^−3^	97.9% @ 10 000 cycles	[95]
25.	Porous carbon spheres (PCSs)//Al	Hybrid: Al metal deposition/stripping // porous‐carbon capacitive/pseudocapacitive cathode	1.3:1 AlCl_3_/[EMIm]Cl; 2	∼200 mAh g^−1^; 150 mAh g^−1^ @2000 mA g^−1^	220 Wh kg^−1^ @ 9000 W kg^−1^	Stable 150 mAh g^−1^ over 1500 cycles; retention % not stated	[33]
26.	h‐MoO_3_ film	Pseudocapacitive electrode‐level test; no full device	Al_2_(SO_4_)_3_; 2.1	∼300 F cm^−3^ @ 5 mV s^−1^	—	—	[80]
27.	Porous TiO_2_ microrods//AC (P‐TiO_2_‐MRs//AC)	Hybrid: battery‐type TiO_2_ intercalation anode // capacitive AC	Aqueous Al‐ion electrolyte;	P‐TiO_2_‐MRs electrode: 111.1 mAh g^−1^ @ 1 49.8 mAh g^−1^ @ 5	26.3 Wh kg^−1^ @ 627 W kg^−1^; 10.9 Wh kg^−1^ @ 5450 W kg^−1^	63% @ 1000 cycles	[70]
28.	α‐MoO_3_@PPy//CuHCF	Pseudocapacitive full cell with faradaic oxide/PBA electrodes	1 M AlCl_3_;	0.50 mAh cm^−2^ @ 1 mA cm^−2^; 0.34 mAh cm^−2^ @ 10 mA cm^−2^	0.33 mWh cm^−2^ @ 1 mA cm^−2^; 0.20 mWh cm^−2^ @ 10 mA cm^−2^	70% @ 1800 cycles; electrode: 88% @ 1000 cycles	[31]
29.	CuFe‐PBA//AC	Hybrid: battery‐type PBA cathode // capacitive AC	1 M Al(NO_3_)_3_;	55 mAh g^−1^ @ ∼1C for CuFe‐PBA cathode	13 Wh kg^−1^	90% @ 1000 cycles; CE 99.1%	[64]
30.	ZIF‐67_rGO//AC	Hybrid: MOF pseudocapacitive/battery‐type electrode // AC; redox mediator	6 mM KFCN + 0.5 M AlCl_3_; 1.2	ZIF‐67_rGO electrode: 349 @ 1	18 Wh kg^−1^ @ 530 W kg^−1^	ZIF‐67_rGO electrode: 93% @ 1000 cycles; full‐device retention not stated	[100]
31.	Mo_15_S_19_//Mo_15_S_19_ symmetric Al‐ion SC	Pseudocapacitive/intercalation symmetric device	1 M AlCl_3_; 0.8	5.2 @ 2	10.4 Wh kg^−1^ @ 736 W kg^−1^	90.1% @ 10,500 cycles	[101]
32.	5% Cu‐doped SnS_2_ nanoflakes, 3‐electrode	Pseudocapacitive/intercalation electrode‐level test	1 M AlCl_3_; −0.2 to 1.0 V vs. reference	140 @ 1	—	∼80% @ 500 cycles	[102]
33.	PEDOT:PSS//AC	Hybrid: pseudocapacitive/intercalation polymer cathode // capacitive AC	1.2 M Al_2_(SO_4_)_3_; 1.6 V	Device: 117 @ 0.3; PEDOT:PSS electrode: 269/ 78 mAh g^−1^ @ 0.2	41.6 Wh kg^−1^ @ 240 W kg^−1^	90% @ 3000 cycles, 0.4 A g^−1^	[103]
34.	PANI‐ES//PANI‐ES symmetric full cell	Pseudocapacitive conducting‐polymer symmetric device	Aqueous Al(NO_3_)_3_; 0.8 V	Full cell: 269 @ 10; PANI‐ES electrode: 317 @ 1	—	88% @ 1000 cycles	[104]

Despite their superior energy densities, AICs often suffer from limited rate capability and poor cycling stability. These limitations primarily stem from the high polarization and strong electrostatic interactions of Al^3+^ ions with the host materials, as well as their large desolvation energy barriers (∼450–500 kJ mol^−1^) as explained in Section 2. In contrast, divalent ions such as Ca^2+^, Mg^2+^ and Zn^2+^ exhibit weaker electrostatic interactions and lower desolvation energies, resulting in improved cycling stability, albeit with lower charge storage capacities (Table 4). To overcome these challenges, future strategies should focus on rational design of electrode materials that can effectively accommodate Al^3+^ ions. This includes developing 2D layered structures with expanded interlayer spacing to facilitate easier diffusion of Al^3+^ within the electrode material and mitigate volume expansion during cycling. Additionally, incorporating functionalized graphene derivatives or conductive carbon scaffolds can enhance electronic conductivity and structural stability. Surface functionalization and the creation of favorable interfacial interactions are also critical for reducing hydration barriers and improving ion accessibility, thereby enhancing overall electrochemical performance of AICs.

Beyond electrode optimization, electrolyte engineering is also a promising route to reduce the Al^3+^ solvation energy and polarization, thereby accelerating desolvation at the electrode/electrolyte interface, enabling faster kinetics and more reversible charge transfer. Collectively, these strategies can effectively mitigate the intrinsic drawbacks of Al^3+^ ions while leveraging their high charge density, paving the way for high‐performance, more stable, and more efficient AICs.

Overall, Al^3+^‐based electrolytes can enable multivalent ion hybrid capacitors because Al^3+^ can transfer three charges per ion and has a high charge density, which promotes strong interactions with redox‐active sites and diffusion‐controlled charge storage (intercalation/pseudocapacitance), thereby increasing capacitance and overall energy density output. The trade‐off is that the same high charge density leads to stronger polarization and high desolvation barriers, often translating to slower interfacial kinetics, so power density and rate capability can be limited, which requires electrode and electrolyte engineering to reduce transport/desolvation limitations. Moreover, the strong electrostatic interactions of Al^3+^ ions with the host materials can lead to lower cycling stability than divalent systems in many cases (Table 4); thus Al^3+^ offers a clear energy density advantage, but typically requires materials/electrolyte optimization (expanded channels/interlayers, conductive scaffolds, and interfacial tuning) to simultaneously achieve high power density and long cycling stability.

## 7 Challenges, Critical Assessment, and Future Perspectives of AICs

Despite the remarkable progress achieved in aluminum‐ion capacitors (AICs), their translation from laboratory‐scale demonstrations to commercially viable energy‐storage technologies remains at an early stage. While many studies report promising electrochemical performance, including high energy density, long cycle life, and rapid charge–discharge capability, several challenges related to scalability, manufacturing, cost, safety, sustainability, and practical device integration must be addressed before large‐scale deployment becomes feasible.

### 7.1 Scalable Electrode Manufacturing

A major challenge for commercialization is the scalable production of high‐performance electrode materials. Many of the most promising AIC electrodes, including MXenes, engineered graphene derivatives, hierarchical porous carbons, metal‐organic frameworks (MOFs), and transition‐metal‐based nanostructures, rely on synthesis routes involving hydrothermal processing, template‐assisted fabrication, selective etching, freeze drying, or multiple post‐treatment steps. Although these approaches provide excellent control over pore structure and electrochemical activity, they often suffer from low throughput, high energy consumption, and limited industrial scalability. Future efforts should prioritize synthesis strategies compatible with roll‐to‐roll processing, slurry coating, continuous carbonization, and large‐scale electrode manufacturing. Particularly attractive are biomass‐derived carbons, solvent‐free porous carbon synthesis routes, and low‐cost chemical activation methods, which offer a pathway toward sustainable and economically viable production.

### 7.2 Electrolyte Limitations and Interfacial Instability

Electrolyte chemistry remains one of the most significant barriers to practical deployment. Chloroaluminate ionic liquids, which enable reversible Al plating/stripping and excellent electrochemical performance, often exhibit moisture sensitivity, corrosive behavior, and relatively high cost. Corrosion of current collectors, packaging materials, and metallic cell components may increase manufacturing complexity and reduce long‐term reliability. Although aqueous electrolytes offer advantages in terms of safety and low cost, their narrow electrochemical stability window and the strong hydration of Al^3+^ introduce challenges associated with sluggish desolvation kinetics and parasitic side reactions. DES, highly concentrated electrolytes, gel electrolytes, and quasi‐solid‐state systems represent promising alternatives, but their long‐term chemical stability, environmental impact, recyclability, and large‐scale manufacturability remain insufficiently understood. Future research should emphasize the development of non‐corrosive, environmentally benign, and low‐cost electrolyte systems that can simultaneously support fast ion transport and stable interfacial chemistry.

### 7.3 Kinetics and Accessibility of Al‐Based Charge Carriers

The high charge density of Al^3+^ and its strong interaction with coordinating ligands or solvent molecules hinder rapid ion transport and insertion into narrow pores or dense host lattices. Many reported electrode materials show promising performance only at modest current densities or low mass loadings, and the contributions of EDLC, pseudocapacitance, and insertion processes are often difficult to deconvolute. A more quantitative understanding of Al‐species speciation, transport, and desolvation at realistic concentrations and operating conditions is needed to guide rational materials design.

### 7.4 Cycling Stability and Mechanistic Ambiguity

Although thousands of cycles are sometimes reported, stable cycling is rarely demonstrated at practically relevant areal loadings, in full devices, and over wide temperature ranges. Side reactions such as electrolyte decomposition, dissolution of redox‐active species, structural degradation of host materials, and passivation of current collectors remain major causes of capacity fading. In addition, the dominant charge‐storage mechanism (anion intercalation, cation insertion, conversion, or surface redox) is not always rigorously established, which complicates meaningful comparison between systems and can lead to over‐optimistic claims about “battery‐like” or “capacitor‐like” behavior.

Although aluminum‐ion capacitors and aluminum‐ion asymmetric supercapacitors are frequently reported to exhibit high cycling stability, their long‐term durability should not be inferred only from capacitance retention, because several coupled degradation pathways can operate during repeated Al^3+^ or chloroaluminate‐ion storage. In aqueous Al‐ion capacitors, the first major failure route is the structural degradation of the redox‐active electrode during repeated multivalent‐ion insertion/extraction. Unlike monovalent Li^+^ or Na^+^, Al^3+^ has a high charge density and strong electrostatic interaction with host lattices, which can slow diffusion, increase local polarization, and generate mechanical/electrochemical stress during cycling. Therefore, even when capacity retention appears acceptable, the electrode may gradually lose accessible redox sites or electrical continuity. This issue is evident from the work on PPy@MoO_3_ for an Al‐ion capacitor reported by Wang et al., where MoO_3_ nanotubes were coated with conductive polypyrrole and used as the anode for reversible Al^3+^ intercalation/deintercalation in aqueous solution [67]. The authors achieved 93% capacitance retention after 1,800 cycles, but the need for a conductive polymer coating itself indicates that bare oxide electrodes require structural/electronic stabilization to withstand repeated Al^3+^ storage. In such MoO_3_‐based systems, possible degradation includes expansion/contraction of the layered oxide framework, weakening of the Mo–O network, partial loss of crystallinity, blocked ion‐transport pathways, and detachment of active material from the conductive network. Although open frameworks can reduce expansion/contraction stresses, defects and dissolution remain major sources of performance degradation and failure. For instance, the open framework of Prussian blue analogs is beneficial because it can accommodate hydrated or partially hydrated multivalent ions more easily than dense oxides [64]. However, long‐term failure may still arise from framework vacancy defects, transition‐metal dissolution, trapped Al species, and progressive distortion of the cyanometallate framework. Thus, even when Al insertion is described as reversible, degradation analysis should include post‐cycling XRD/Raman/FTIR, elemental mapping, and electrolyte analysis to determine whether the framework remains intact or whether active species gradually dissolve into the aqueous electrolyte. Apart from the oxide and inorganic materials, conducting‐polymer electrodes suffer from swelling/shrinkage, overoxidation, mechanical softening of the film, loss of adhesion to carbon cloth, and changes in ion‐accessible free volume. For example, Ai et al. reported reversible Al‐ion intercalation in PEDOT:PSS electrodes for aqueous electrochemical capacitors [103]. Since Al^3+^ interacts strongly with sulfonate or oxygen‐containing groups in polymeric matrices, repeated insertion can also produce kinetically trapped Al species, reducing the number of reversible redox sites and increasing interfacial resistance. Therefore, Al‐ion polymer capacitors should be evaluated not only by capacitance retention but also by changes in redox peak separation, CE, ESR, charge‐transfer resistance, and polymer chemical state after cycling. In carbon‐based Al‐ion hybrid supercapacitors using chloroaluminate ionic liquids, passivation and interfacial instability have a different origin. Jiao et al. assembled an aluminum‐ion asymmetric supercapacitor using MWCNTs as the cathode and AlCl_3_[EMIm]Cl ionic liquid as the electrolyte, and the reported storage mechanism involved both ${\mathrm{AlCl}}_{4}^{-}$ adsorption/desorption on the carbon surface and limited ${\mathrm{AlCl}}_{4}^{-}$ intercalation/deintercalation into graphitic layers at low current density [94]. In this system, degradation may arise from irreversible trapping of chloroaluminate anions in graphitic domains, expansion or exfoliation of graphitic layers during anion intercalation, corrosion or side reactions at the Al negative electrode, and changes in ionic‐liquid speciation. The cycle retention of 82.4% after 3,500 cycles indicates reasonable stability, but the remaining loss may be associated with gradual consumption or imbalance of ${\mathrm{AlCl}}_{4}^{-}$/$Al_{2}{\mathrm{Cl}}_{7}^{-}$ species, increased viscosity or transport limitations, and interfacial film formation. These mechanisms are also relevant to newer aluminum‐based hybrid supercapacitors that attempt to avoid intercalation‐induced damage by using high‐surface‐area porous carbon electrodes. Similarly, Sun et al. designed an Al‐ion hybrid device using nitrogen‐doped micro–mesoporous carbon spheres as the positive electrode and an Al negative electrode [55]. The authors explicitly argue that porous carbon promotes anion adsorption/desorption and relieves intercalation stress compared with graphite‐type intercalation electrodes. However, when charge storage occurs mainly through surface adsorption of ${\mathrm{AlCl}}_{4}^{-}$ rather than deep intercalation, structural pulverization is minimized, but failure can still occur through pore blockage, loss of wettability, surface functional‐group degradation, electrolyte decomposition, and unstable Al plating/stripping at the negative electrode. Another issue that must be addressed in supercapacitors is electrolyte decomposition and self‐discharge, especially in aqueous electrolytes. The aqueous Al^3+^ electrolyte is attractive because of safety and cost, but the practical voltage window is limited by water decomposition, hydrogen evolution at negative potentials, oxygen evolution at positive potentials, pH variation, and formation of hydrolyzed aluminum species. For instance, the work by Rahman et al. showed the importance of electrolyte optimization to reduce the failure modes in supercapacitors [227]. The authors used aqueous 1 M AlCl_3_ and AlCl_3_–PVA gel electrolytes. The gel electrolyte allowed a wider potential window of about 2 V compared with about 1.5 V in the pristine aqueous electrolyte and significantly suppressed self‐discharge. This result suggests that one failure mode in aqueous Al‐ion capacitors is not simply electrode degradation but instability of the electrode/electrolyte interface and continuous internal charge leakage. Gel or quasi‐solid electrolytes can mitigate this by reducing free‐water activity, limiting ion crossover, slowing parasitic reactions, and improving interfacial contact. However, one must be cautious as they may also introduce their own degradation through polymer drying, reduced ionic conductivity, or accumulation of immobile Al complexes.

Overall, the available literature indicates that performance degradation in AICs originates from multiple interconnected failure mechanisms involving the electrodes, electrolytes, and their interfaces. Depending on the device chemistry and charge‐storage mechanism, these degradation pathways may include structural deterioration of host materials during repeated Al‐species insertion/extraction, lattice distortion, crack formation, particle pulverization caused by volume expansion and contraction, dissolution of redox‐active components, swelling and chemical degradation of polymer electrodes, irreversible trapping of chloroaluminate ions, pore blockage in porous carbons, unstable Al plating/stripping, electrolyte decomposition, and self‐discharge processes. Importantly, high capacitance retention alone should not be considered definitive evidence of long‐term electrochemical stability, as gradual loss of active sites, loss of electrical contact, interfacial deterioration, and changes in electrolyte composition can occur without immediately causing severe capacity fading. Therefore, future studies should combine electrochemical testing with comprehensive post‐mortem and operando characterization techniques, including structural, chemical, morphological, and interfacial analyses, to identify the dominant degradation pathways under realistic operating conditions. Establishing clear correlations between charge‐storage mechanisms and failure modes will be essential for the rational design of durable, safe, and commercially viable Al‐ion supercapacitor technologies.

### 7.5 Device‐Level Metrics and Scalability

Many reported AICs are evaluated using laboratory‐scale coin cells or low‐mass‐loading electrodes under highly optimized conditions. Practical implementation, however, requires demonstration of high areal capacity, thick electrodes, industrially relevant binders and current collectors, and scalable cell configurations such as pouch or cylindrical cells. At present, many Al‐based devices are assembled in symmetric or asymmetric configurations using carefully optimized laboratory electrodes, making it difficult to assess their performance under commercially relevant conditions. Furthermore, system‐level metrics are rarely reported. Parameters such as gravimetric and volumetric energy and power density at the packaged‐cell level, cycle life under realistic duty cycles, self‐discharge behavior, calendar life, thermal stability, shelf life, and performance across a wide temperature range are essential for evaluating practical viability. Without such information, meaningful comparisons with state‐of‐the‐art carbon‐based supercapacitors, Li/Na/K‐ion capacitors, and batteries remain challenging. Future studies should therefore move beyond material‐level benchmarking and emphasize device‐level validation under realistic operating conditions. Standardized testing protocols, reporting practices, and scale‐up demonstrations will be essential for establishing the true commercial competitiveness of AIC technologies.

### 7.6 Sustainability and Safety Trade‐Offs

Although aluminum metal is abundant, inexpensive, and highly recyclable, the overall sustainability of AIC technology depends on more than the choice of charge carrier. The environmental footprint of electrode synthesis, electrolyte production, dopants, conductive additives, and complex nanostructured architectures must also be considered. Some ionic liquids, organic solvents, fluorinated compounds, and redox‐active additives may introduce environmental, health, or safety concerns that could offset the intrinsic sustainability advantages associated with aluminum. Consequently, future research should adopt a holistic cradle‐to‐grave perspective when evaluating new AIC chemistries. Life‐cycle assessment, material criticality analysis, toxicity evaluation, recyclability studies, and end‐of‐life management strategies should be incorporated alongside electrochemical performance metrics. Such assessments will be crucial for identifying truly sustainable materials systems and ensuring that future AIC technologies meet increasingly stringent environmental and regulatory requirements.

### 7.7 Flexible and Wearable Energy Storage Systems

The growing demand for wearable electronics, portable sensors, and flexible energy‐storage devices creates new opportunities for AIC technology. Flexible carbon materials, conducting polymers, MXenes, and carbon‐cloth‐supported electrodes have already demonstrated promising electrochemical performance in laboratory‐scale devices. Furthermore, gel and quasi‐solid‐state electrolytes offer additional advantages by improving mechanical flexibility, suppressing electrolyte leakage, and enhancing operational safety. Nevertheless, significant challenges remain in maintaining stable electrochemical performance under repeated bending, stretching, and deformation. Future efforts should focus on the development of mechanically robust electrode architectures, flexible current collectors, and durable solid‐state electrolyte systems capable of sustaining long‐term operation under realistic wearable conditions.

In summary, the commercialization of AICs will require a holistic approach that integrates materials innovation with scalable manufacturing, safe and sustainable electrolyte design, and practical device engineering. While substantial progress has been achieved in understanding charge‐storage mechanisms and improving electrochemical performance, future advances must increasingly focus on manufacturability, cost reduction, long‐term reliability, environmental sustainability, and industrial scalability. Equally important is the establishment of standardized device‐level evaluation protocols that include realistic mass loadings, packaged‐cell performance metrics, temperature‐dependent operation, and life‐cycle considerations. Combining mechanistic understanding with techno‐economic assessment, sustainability analysis, and rigorous device validation will be crucial for accelerating the transition of AICs from promising laboratory concepts to commercially competitive energy‐storage technologies for grid storage, portable electronics, and flexible wearable systems.

## 8 Conclusion and Outlook

In this review, we have highlighted the unique opportunities offered by aluminum‐ion supercapacitors, including the high theoretical charge storage associated with the trivalent Al^3+^ cation, the abundance and recyclability of Al metal, and the rich chemistry of aqueous, non‐aqueous, and hybrid electrolytes. Significant progress has been achieved in electrode design from hierarchical porous carbons and transition‐metal oxides to emerging 2D materials such as graphene and MXenes enabling improved ion accessibility, electrical conductivity, and structural stability. Conductive polymers, MOFs/COFs, and organic redox‐active materials represent promising yet relatively underexplored electrode platforms for aluminum‐ion supercapacitors. Their tunable chemistry, structural versatility, and potential for tailored ion‐transport pathways offer significant opportunities for future development. Further advances will require improvements in electrical conductivity, electrolyte compatibility, cycling durability, and mechanistic understanding, supported by advanced characterization and computational studies. Continued progress in these emerging material classes may enable next‐generation AICs with enhanced energy density, rate capability, and device flexibility. Parallel advancements in electrolyte chemistry, particularly in aqueous WISE/HEE systems, ionic liquids, and hybrid DES formulations, have expanded the ESW and facilitated reversible Al plating/stripping behavior.

Despite these achievements, several fundamental and practical challenges still prevent AICs from competing with mature Li‐ion batteries and commercial carbon‐based supercapacitors. On the electrolyte side, many of the best‐performing Al‐based systems rely on corrosive, moisture‐sensitive ionic liquids or DES whose long‐term stability, cost, and environmental impact remain insufficiently assessed. In aqueous and highly concentrated electrolytes, the strong hydration and large desolvation penalty of Al^3+^ lead to sluggish interfacial charge transfer and incomplete reversibility, while side reactions can degrade both electrodes and electrolytes. At the electrode level, incompatibility between host structures and multivalent charge carriers, limited ion accessibility in narrow pores, and structural degradation during cycling often constrain performance to modest mass loadings and narrow operating conditions. Mechanistic ambiguities, such as the relative contributions of anion intercalation, cation insertion, conversion reactions, and surface redox, further complicate the comparison of reported systems and can obscure realistic performance limits. Finally, device‐level metrics for thick electrodes and practical cell formats are still scarce, making it difficult to predict the true scalability and competitiveness of AICs, including from sustainability and safety perspectives.

Addressing these challenges will require coordinated advances in materials, electrolytes, characterization, and device engineering. Synergistic strategies that combine interphase engineering, defect and pore‐structure modulation, and hybrid electrolyte optimization are needed to alleviate desolvation barriers, suppress parasitic reactions, and stabilize Al/electrolyte interfaces. Future research should prioritize mechanistically informed design, integrating operando and in situ diagnostics with multiscale computational modeling to quantify Al‐species speciation, transport, and reaction pathways under realistic operating conditions. However, data standardization and quality control are necessary to establish a clear feedback loop in between artificial inteligence‐driven design and experiments [228]. Equally important is the translation of promising chemistries into flexible, quasi‐solid, and scalable device architectures, accompanied by rigorous benchmarking of energy/power density, cycling stability, self‐discharge, and safety at the device level.

Overall, the convergence of innovative materials engineering, rational electrolyte design, and deeper mechanistic insight can position AICs as a promising pathway toward sustainable, high‐performance, and recyclable energy‐storage systems beyond conventional lithium‐ion technology. If the key bottlenecks identified here, especially electrolyte stability, interfacial kinetics, mechanistic clarity, and device‐level scalability, can be addressed in a holistic manner, AICs may evolve from an intriguing laboratory concept into a viable technology for future grid, mobility, and wearable applications.

## Author Contributions

**Smita Talande**: conceptualization, visualization, writing – original draft, Writing – review & editing; **Ievgen Obraztsov**: writing – original draft, visualization; **Vishal Shrivastav**: writing – original draft, visualization, funding acquisition; **Mahima Khandelwal**: writing – original draft, visualization, funding acquisition; **Mansi**: writing; **Michal Otyepka**: funding acquisition, resources, writing – review & editing; **Radek Zbořil**: funding acquisition, resources, writing – review & editing; **Aristides Bakandritsos**: funding acquisition, project administration, resources, supervision, conceptualization, writing – review & editing.

## Conflicts of Interest

The authors declare no conflicts of interest.

## Data Availability

Data sharing not applicable to this article as no datasets were generated or analysed during the current study.
